# Inventorization and Consensus Analysis of Ethnoveterinary Medicinal Knowledge Among the Local People in Eastern India: Perception, Cultural Significance, and Resilience

**DOI:** 10.3389/fphar.2022.861577

**Published:** 2022-04-29

**Authors:** Suman Kalyan Mandal, Chowdhury Habibur Rahaman

**Affiliations:** Ethnopharmacology Laboratory, Department of Botany (DST-FIST and UGC-DRS SAP-II), Visva-Bharati University, Santiniketan, India

**Keywords:** ethnoveterinary medicine, livestock disease, quantitative ethnobotany, cultural value, Eastern India

## Abstract

Livestock is the main backbone of the rural economy of an agriculture-based country like India. To mitigate the economic loss due to livestock’s poor performance and illness, folk phytotherapy for livestock healthcare is still actively practiced in India. Literature survey revealed that the laterite region of eastern India, characterized by its cultural, ethnic, and biological diversities, as well as topographical uniqueness, lacks comprehensive information on ethnoveterinary medicinal knowledge. The objective of the present study includes documentation of traditional knowledge of ethnoveterinary medicine (EVM) from the northern laterite region in eastern India. Ethnoveterinary medicinal data were collected using a semi-structured questionnaire, free listing, and focus group discussions. The factor for informants’ consensus (Fic), fidelity level (FL), and cultural value (CV) index have been employed for quantitative analyses. Jaccard index (JI) was used to check the knowledge similarity. Altogether, 1,234 citations were made by 132 participants. In total, 232 recorded ethnomedicinal species are used for preparing 306 remedies to treat 79 health disorders of livestock. Recorded species are distributed in 92 families, and *Fabaceae* is identified as the most medicinally diversified. Uses of 24 angiospermic taxa, one pteridophyte, and two fungal species were exclusively new to the existing inventory of Indian traditional ethnoveterinary medicine. In 20 disease categories, the informant consensus (Fic) value ranges from 0.4 to 0.83. According to the FL value and use-mention factor, 23 EVM plants have been identified as the most important species in the respective disease categories. Value of CV index highlighted nine species as culturally most significant (CV ≥ 0.0025 and frequency of citation ≥20) in the laterite region of eastern India. A large extent of recorded data are quite worthy for the Indian folk veterinary medicinal repository. A handful of new data reported here and statistically justified culturally most significant species will provide the golden opportunity for bioprospecting research.

## 1 Introduction

From the beginning of human civilization, the need for animal domestication was realized by the ancient people in every step of their shifted livelihood from hunting to farming. Over the past 11,000 years, varieties of animals have been domesticated by humans for food, secondary products, labor, and companionship (Ahmad et al., 2020; Cucchi and Arbuckle, 2021). Simultaneous developments of traditional healthcare management systems for domesticated animals have been shaped according to the continuous evolution of knowledge, culture, and local biodiversity. Traditional knowledge associated with the healthcare management of livestock is the basis of ethnoveterinary medicine (EVM). It includes people’s understanding, expertise, approach, way of application, and faith. Documentation of this age-old non-codified traditional knowledge for its larger prospects and academic interest has revolved around the wheel of research in ethnobotany that deals with the multidisciplinary approach of people–plant interactions (Gomez-Beloz, 2002). In the last three decades, many scientific articles on ethnoveterinary medicine have been published from different parts of the world (Yineger et al., 2007; Shen et al., 2010; Zia-ud-Din et al., 2010; Aziz et al., 2018; Erarslan and Kültür, 2019; Chakale et al., 2021), indicating the growing interest of the researchers in this field of ethnomedicine.

In India, the tradition of livestock rearing is prehistoric and sacrosanct. To date, livestock plays a vital role in shaping the rural economy by providing livelihood to two-thirds of rural communities, mainly the landless daily laborers, marginal and small-scale farmers, and women (Mutua et al., 2020).

Societal acceptance, emotional attachment, and economic benefit of the domesticated animals lay the foundation of livestock healthcare management in India long before the Vedic age (Somvanshi, 2006). From the very beginning, local biodiversity contributes immensely to India’s folk veterinary medicinal practices. Preparation of databases on ethnoveterinary medicinal knowledge and documentation of related medicinal plants in India are getting priority for its better scientific exploitation, resulting in a vast repository of research and review articles, thesis, and books on this subject (Jain, 1999; Pande et al., 2007; Dey and De, 2010; Galav et al., 2013; Bharali et al., 2015; Khandelwal, 2017). For ethnoveterinary data collection, focuses have been made on a particular ethnic group (Gaur et al., 2010; Kumar et al., 2012; Rajkumari et al., 2014), specific geographical area (Bharati and Sharma, 2010; Lakshminarayana and Rao, 2013; Manoranjotham and Kamaraj, 2016), particular animal group (Das, 2011; Sharma et al., 2012; Shrivastava et al., 2012; Jayakumar et al., 2017), and specific disease or ailment conditions (Mishra, 2013; Chouhan and Ray, 2015).

In West Bengal, a state of eastern India, scientific documentation of medicinal plants and related traditional knowledge has primarily been focused on ethnomedicine of human importance (Rahaman and Saha, 2011; Mondal and Rahaman, 2012; Banerjee et al., 2013; Das and Rahaman, 2014; Chaudhury et al., 2017). Phytotherapeutic knowledge of veterinary importance, traditional practitioners of veterinary medicine, and their role in primary healthcare for veterinary diseases and ailments have been overlooked initially. The documentation of ethnoveterinary medicines in West Bengal started much later, resulting in sporadic and scanty knowledge documentation (Pal, 1980; Mandal and Chauhan, 2000; Ghosh, 2003; Bandyopadhyay and Mukherjee, 2005; Mandal and Rahaman, 2014; Saha et al., 2014). Specifically, few reports on ethnoveterinary medicine have been published from districts such as Bankura, Midnapore, Purulia, Birbhum, and Burdwan, which comprise the laterite region of West Bengal (Mukherjee and Namahata, 1988; Rahaman et al., 2009; Dey and De, 2010; Mandal and Rahaman, 2016). A perusal of literature indicates a potential lack of ethnoveterinary medicinal information in this area.

In the laterite region of West Bengal, a large section of the local people mostly depends on mixed crop cultivation and livestock rearing. In this socio-economic spectrum, livestock plays a definite role in balancing the core economy of this area. Livestock keepers are concerned about the healthcare of their mute animals and mobilize themselves for innovating their ways of keeping these animals healthy.

Government-supported livestock healthcare facilities are provided in the livestock sector but remain inadequate in substantial numbers of field veterinarians, supporting staff, and health centers (NAVS, 2014). This healthcare system mostly focuses on artificial insemination of cattle, vaccination against infectious diseases, their control, and investigation programs (Ahuja et al., 2008). So, in most cases, state government-supported livestock healthcare facilities meagerly fulfill the needs of livestock owners in this region. For alleviating common health issues, folk therapies for treating animals have become obligatory and are actively practiced to date. However, ethnoveterinary medicines (EVM) have limitations in rapidly controlling epidemic infectious diseases and acute life-threatening bacterial infections. Like other folk therapeutic systems, traditional veterinary medicinal knowledge is also persisting as a non-codified system transmitted orally from generation to generation in the laterite region of West Bengal. However, the modernization of the traditional societies with rapid socio-economic, environmental, and technological changes can inevitably cause erosion of this knowledge. This ancient therapeutic knowledge remains mostly unexplored, which needs a thorough scientific study before being lost forever.

In order to add more objectivity to the ethnobiological research, the application of statistical indices for quantifying ethnobotanical data is gradually increased among ethnobotanists worldwide (Andrade-Cetto and Heinrich, 2011; Medeiros et al., 2011). Scientists now prefer ethnobotanical information measured by suitable statistical indices for bioprospecting of natural products as the ethno-guided information or leads provide more success rate than the taxonomy-guided and randomly selected leads (Rahaman, 2017). There are a few research articles on ethnoveterinary medicine published from India, where data have been analyzed using some popular statistical indices such as factor for informants’ consensus (Fic), use value (UV), relative frequency of citation (RFC), and fidelity level (FL) (Kumar et al., 2012; Kumar and Bharati, 2013; Yabesh et al., 2014; Mandal and Rahaman, 2016; Prakash et al., 2021). The formulas of FL, UV, and RFC are mainly based on its use reports and are simple percentage calculations. Nevertheless, how far are these indices relevant to effectively quantify the usefulness of a plant for a specific purpose? Rather they can be considered “statistically insufficient” to assess the true reflection of the cultural importance of a species (Leonti, 2022). In order to evaluate the actual degree of cultural acceptance of a species and its importance as a whole, a much-dedicated quantitative index based on cultural consensus should be included.

In this context, the present work has been opted for the following goals:

• To document the existing vast ethnoveterinary medicinal knowledge from the northern laterite zone in West Bengal,
• To explore the perception and depth of the knowledge among the local people of this area,
• To quantify the ethnobotanical data using suitable statistical indices.

## 2 Materials and Methods

### 2.1 Study Area

The state of West Bengal is located in the far most eastern part of India (22° 59′ 12.325″ N and 87° 51′ 17.914″ E). The laterite zone of West Bengal is characterized by its cultural, ethnic, and biological diversities and topographical uniqueness. It is the extended part of the eastern fringes of the Chota Nagpur plateau, which includes the western and central parts of Bankura district; western parts of Medinipur and Burdwan districts; and western, south-central, and northern parts of Birbhum district (Das, 2014). Laterite region is spanning across the latitude 22°00′ to 24°30′ N and longitude 86°45′ to 87°50′ E, and the altitude varies between 115 and 45 m. It covers an area of approximately 7,700 km^2^, representing 22.3% of the total geographical area of the state (Hunday and Banerjee, 1967). Soil type is red and lateritic. The climate is “dry sub-humid mega thermal.” The dry deciduous forests in this region represent nearly 15.2% of the total state geographical area. According to the Census of 2011, the percentage of Scheduled Tribes in this zone is 11.85%, and Scheduled cast is 26% (Census of India, 2011).

**FIGURE 1:**
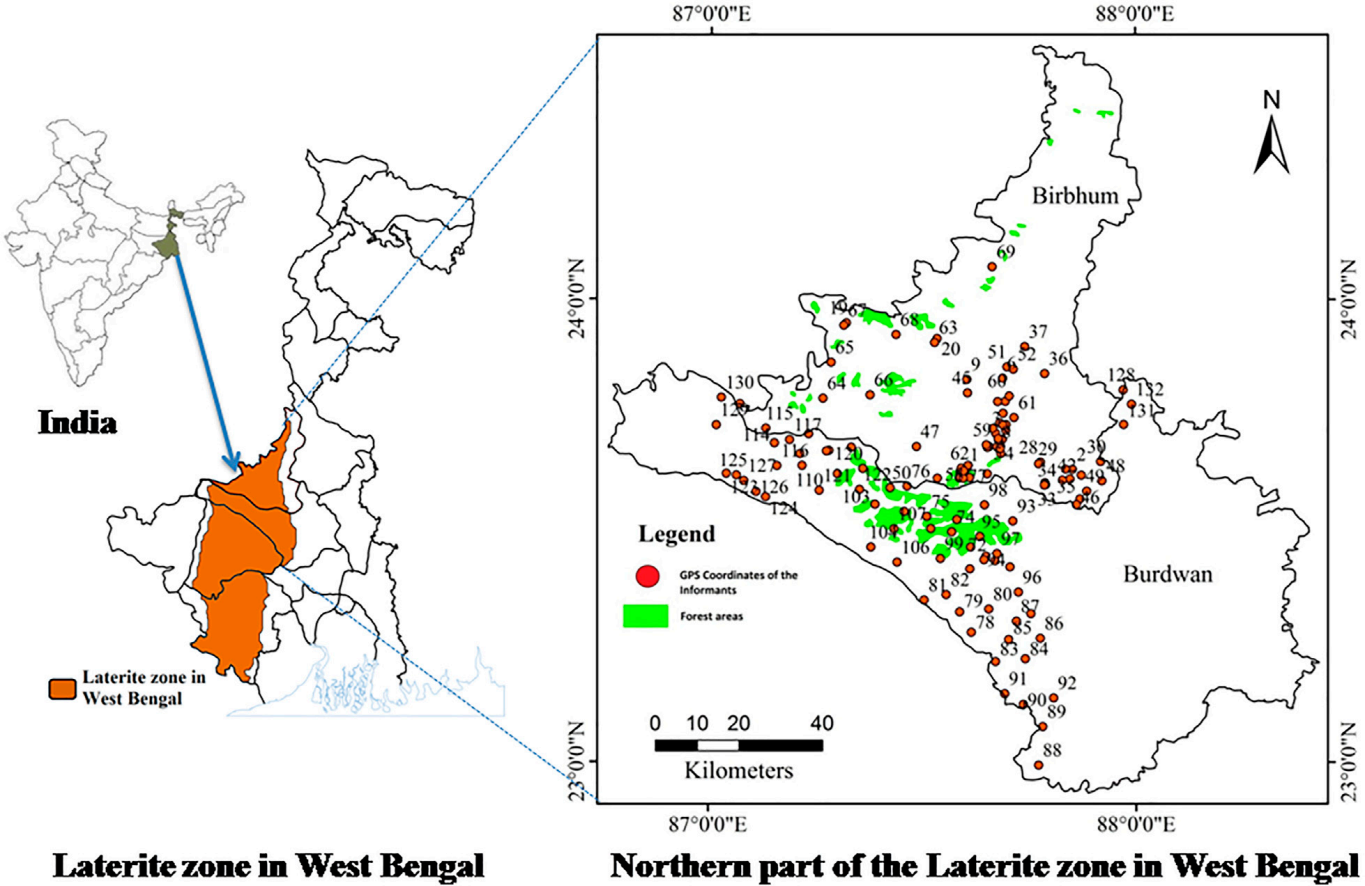
Map of the study area and GPS-guided locations of the participants’ residence in the northern laterite region of West Bengal, India.

The study area of the present work is restricted to the northern part of the laterite zone of West Bengal, which includes mainly the western part of Burdwan district and western, south-central, and northern parts of Birbhum district. This part of the laterite region covers an area of approximately 2,290 km^2^, which represents 29.74% of the total laterite cover of West Bengal. Altogether, 21 blocks have been selected for the present study, 11 from Burdwan district and 10 from Birbhum district, which fall within the northern laterite region of West Bengal (Figure 1). Block is one of many small divisions of a district representing a compact area consisting of several villages.

### 2.2 Data Collection

**FIGURE 2:**
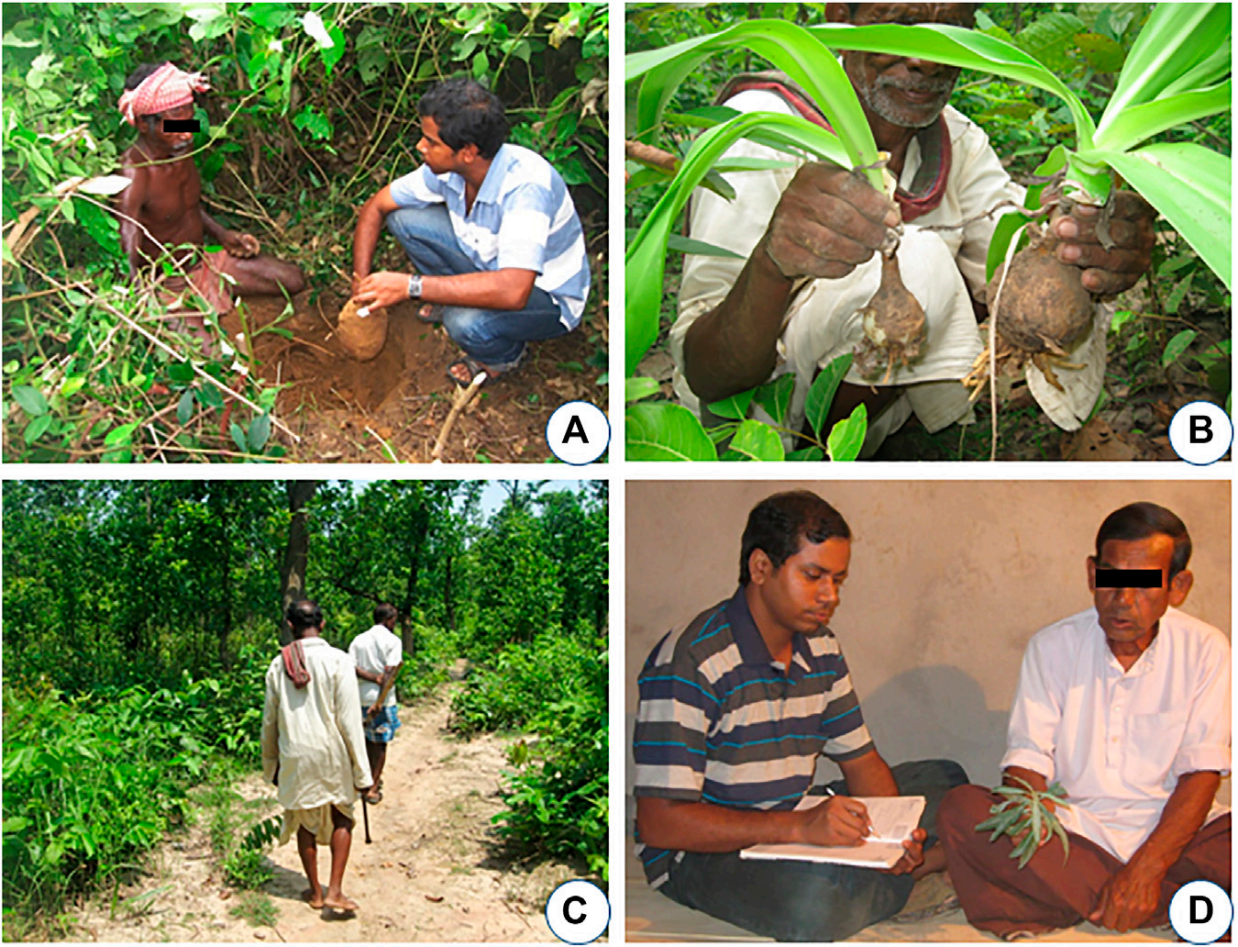
Ethnoveterinary data collection and identification of EVMPs: *In loco* identification of **(A)** *Pueraria tuberosa* (Roxb. ex Willd.) DC. and **(B)** *Crinum asiaticum* L. **(C)** Guided tour in a “dry-deciduous forest” of the northern laterite region, West Bengal, India. **(D)** Use of a twig of *Solanum glaucophyllum* Desf. as visual stimuli during interaction with one of the aged participants.

Systematic field surveys were conducted in 21 blocks of the Birbhum and Burdwan districts in different seasons of a year from 2011 to 2018. A total of 132 participants were interviewed with the help of a semi-structured questionnaire, free listing, and focus group discussion after clearly presenting the purpose of the study and its outcome before the participants as per the stipulations of Nagoya Protocol 2014. Prior informed consent (PIC) was taken from each informant verbally before collecting the data on herbal knowledge. For the collection of data, during the field survey here, an attempt has been made to follow the best field practice as critically described earlier (Heinrich and Verpoorte, 2014; Heinrich et al., 2018; Weckerle et al., 2018), as per the code of ethics mentioned by the International Society of Ethnobiology (2006). Various visual stimuli were employed for plant identification and related data collection from the aged, and individuals with restricted movement, and female participants. For this, the fresh and/or dried plants and their parts, herbarium specimens, and photographs of the plants were exhibited to the participants to identify those plants and collection of associated ethnobotanical information (Figure 2). The authenticity of the information has always been confirmed by cross-checking other participants of the same and the other localities with the same set of questions and visual clues (Martin, 1995; Vogl et al., 2004; Thomas et al., 2007). Information on the local name of the plants, their parts use, collection, preservation, mode of preparation of remedy, its administration, and dosages were recorded in detail. The geographic location of the participants’ permanent residence was noted in the form of global positioning system (GPS) coordinates. Their photographs and socio-demographic information were also recorded.

### 2.3 Collection of Plant Specimen and Preparation of Herbarium

Plants having ethnoveterinary medicinal uses were collected following the guideline set by the National Medicinal Plants Board, India (NMPB, 2015). Herbarium specimens have been prepared with the collected plant samples having specific field numbers following the techniques suggested by Jain and Rao (1977). For future reference, all the herbarium specimens have been kept in the departmental Herbarium (Visva-Bharati Herbarium, Department of Botany, Visva-Bharati, Santiniketan, India).

### 2.4 Identification of the Plant Specimen

The collected angiosperms were identified with the help of different Floras of West Bengal and its adjoining states (Guha Bakshi, 1984; Sanyal, 1994.; Saxena and Brahmam, 1994-1996; BSI, 1997; Paul et al., 2015; Ranjan et al., 2016). The following literature has been consulted to identify the collected species of fungi (Purkayastha and Chandra, 1985; Singer, 1986; Bilgrami et al., 1991) and species of pteridophytes (Dixit, 1984; Fraser-Jenkins, 2008). Besides, herbarium specimens housed at Central National Herbarium (CAL), BSI, Howrah, India, have also been consulted.

Two specialists finally confirmed the identification of the collected plant species after critically examining the voucher specimens.

### 2.5 Nomenclature Update

The nomenclature of all the collected plant species has been updated following the standard websites such as World Flora Online^1^, The Plant List^2^, Tropicos^3^, and Germplasm Resources Information Network^4^.

### 2.6 Data Analysis

#### 2.6.1 Qualitative Analysis of Ethnobotanical Data

Recorded information on the local name of the plant, updated scientific name, family, voucher specimen number, parts used, collection source, mode of preparation of the remedies, and administration with dosages were tabulated systematically.

#### 2.6.2 Quantitative Analysis of Ethnobotanical Data

The following indices are included in this study.

##### 2.6.2.1 Factor for Informants’ Consensus

One of the most widely used indices is the factor for informants’ consensus (Fic), proposed by Heinrich et al. (1998) based on the equation of Informant Agreement Ratio introduced by Trotter and Logan (1986).

For Fic analysis, it is necessary to classify health conditions/illnesses into broad disease categories. The formula of the Fic is

$$\text{Fic }=\;\frac{\text{Nur }-\text{ Nt }}{\text{Nur }-\;1},$$

where Nur refers to the number of use-reports for a particular use category/disease category and Nt refers to the number of taxa used for a particular use category/disease category by all participants.

##### 2.6.2.2 Fidelity Level

In order to measure the reliability of the information provided by the participants, the fidelity level (FL) index is used. The value of FL is calculated following the formula:

$$\text{FL}\left(\text{\%}\right)=\frac{\text{Np }}{\text{N}}\times 100,$$

N_P_ is the number of respondents that claim the use of a plant species to treat a particular disease, and N is the number of respondents that use the plant as a medicine to treat any given disease (Friedman et al., 1986).

A high FL value (100%) is obtained for a plant when all the participants refer to it for the same purposes.

##### 2.6.2.3 Cultural Value Index

The index is employed to understand the overall importance of a plant species in a particular culture (Reyes-García et al., 2006), and it is determined by the following formula:

CV=[NUsNC]×[FCsN]×[∑u=u1uNC ∑i=i1iNURuiN],

where *s* indicates the ethno-species for determining cultural value. The value of the first factor of the index is obtained by dividing the total number of uses reported for the ethno-species *s* (NUs) by the total number of use categories considered in the study (NC). The second factor of this index does mean the relative frequency of citation (RFC), and it is obtained by dividing the frequency of citation of that particular species (FCs) by the total number of participants interviewed (N). Here, the third factor indicates the cultural importance (CI) of the species *s* and is calculated based on the sum of all the use reports (UR) for that particular species. Finally, the CV value is obtained by multiplying the values of these three factors.

##### 2.6.2.4 Preference Ranking Exercise

Preference ranking exercise is carried out among the selective key participants to find preferable species out of all the plant species cited by the participants for a specific purpose (Martin, 1995). It is based on a scoring system where points ranging from 0 to 10 are given by each of the selective key participants according to their preference. The highest scoring point (i.e., 10) is given to the most preferred species, but the lowest point is 0, which is assigned to the least preferred species. Based on the total score, all the species are then ranked.

##### 2.6.2.5 Jaccard Index

The similarity of knowledge among the participants is assessed with the help of the Jaccard index (JI) using the following formula:

$$\text{JI}=\frac{\text{c}\times 100}{\left(\text{a }+\text{b}\right)\;-\text{ c}},$$

where a and b are the number of plants known to the participants of areas A and B, respectively, and c is common to both A and B (Hamers, 1989).

##### 2.6.2.6 Spearman Rank-Order Correlation

The Spearman rank-order correlation analysis is performed using R Studio 1.1.442 software to measure the strength and direction of correlation between the variables (Michelson and Schofield, 2002).

## 3 Results

### 3.1 Socio-Demography of the Participants

**FIGURE 3:**
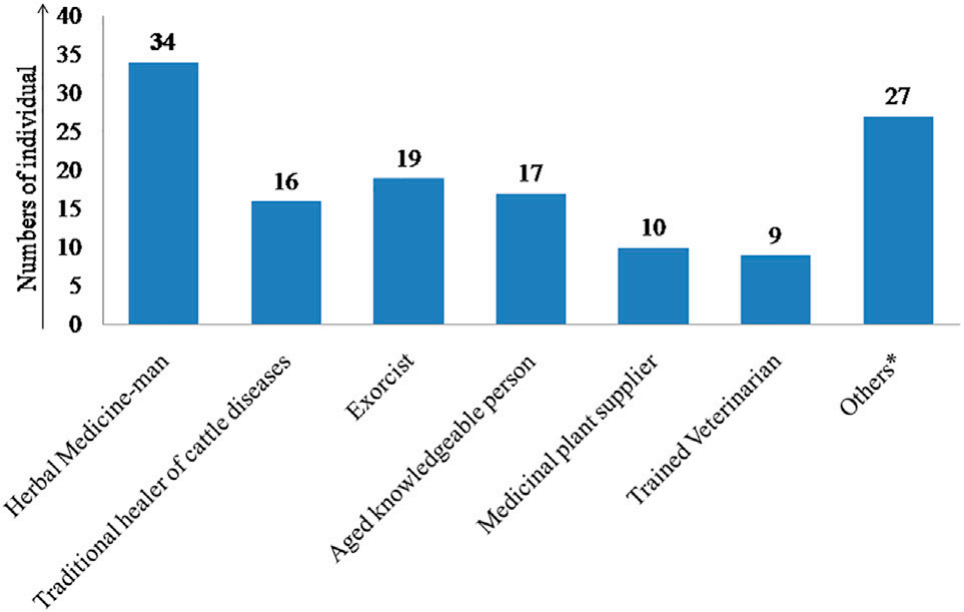
Categories of participants and numbers of individuals interviewed in each category (*Others include farmer, herdsman, shepherd, and milkman).

Altogether 132 participants were interviewed, of which 109 persons are male (82.58%) and the remaining 23 participants are female (17.42%). Participants’ socio-demographic information is presented in Supplementary Table S1. All the participants belong to seven categories according to their social designations (Figure 3). GPS coordinates of the participants’ residential location have been indicated in the study area map, which will help future researchers working in the related fields. Besides, it will strengthen the authenticity and intellectual property rights (IPR) of the knowledge providers.

It was observed in the present investigation that, among all the knowledge transmission pathways, “vertical transmission” of knowledge is predominant as in most of the cases (56.06%), the traditional wisdom is conveyed from the parents to their descendants.

**FIGURE 4:**
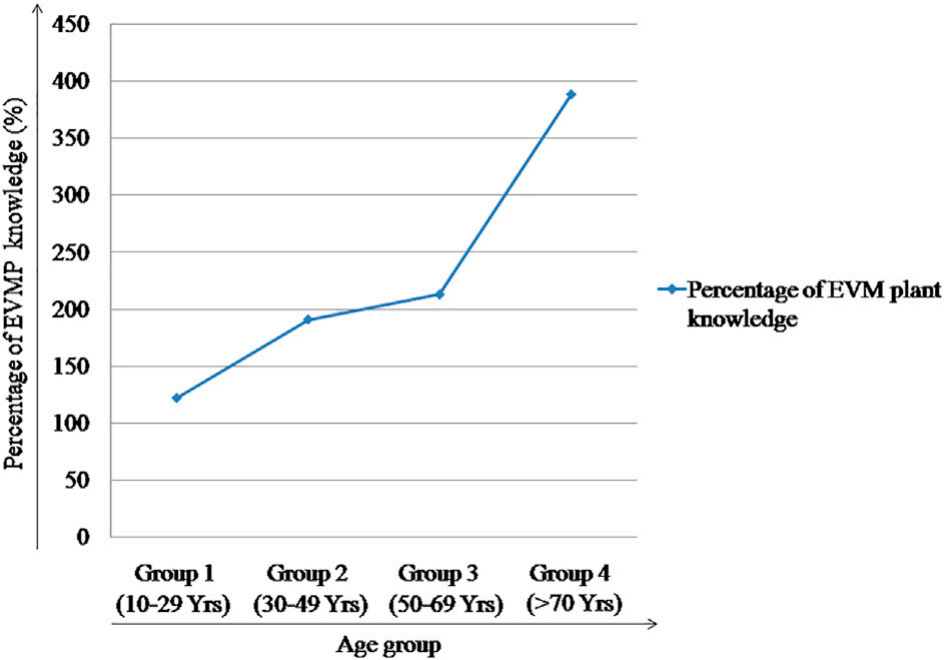
Percentage of EVM knowledge distributed in different age groups of the participants.

With the increase in age, the knowledge domain of the knowledge holder is gradually widened. Here, the knowledge about ethnoveterinary medicinal plants has been compared between the participants belonging to four different age groups employing the percentage of EVM plant knowledge possessed by them (Figure 4). The results show that the participants aged 70 years and above have extensive knowledge as they reported the highest numbers of EVM plants along with their names and uses.

Altogether five barriers or constraints have been identified that hamper the knowledge transfer among the participants in the area. The most vital barrier is the modernization of the traditional society, which makes the younger generation less interested in their age-old folk therapeutic practices. The second barrier is the unavailability of forest resources. The third one is the cultural and linguistic differences between two persons of different communities. The fourth one is faith in modern medicine. In many cases, the quick healing potentiality of modern medicine attracts local people, and they become accustomed to it. The last constraint here in knowledge transmission is the “secret medicine,” which is kept secret by the knowledge holder. Before his death, the knowledge is carried forward to a reliable family member only.

### 3.2 Ethnoveterinary Medicinal Plants

#### 3.2.1 Taxonomical Information

A total of 232 EVMPs have been recorded from the northern laterite part of West Bengal (Supplementary Table S2). All these EVMPs belong to 201 genera and 92 families. Among the recorded plant species, 194 species are of dicotyledonous plants, 33 species belong to monocotyledons, two species, namely, *Adiantum philippense* subsp. *philippense* and *Lygodium flexuosum* (L.) Sw. are of the pteridophyte group, and three species, namely, *Amanita vaginata* var. *alba* Gillet, *Lycoperdon perlatum* Pers., and *Termitomyces heimii* Natarajan belong to the group of fungi. Among the reported 92 plant families, *Fabaceae* is represented by the highest number of plant species (21 species). Two families, *Malvaceae* and *Lamiaceae*, are represented by ten species each; three families, *Apocynaceae*, *Asteraceae,* and *Euphorbiaceae*, are represented by nine plant species each, and eight species were recorded from the *Solanaceae*. Each of families *Acanthaceae* and *Convolvulaceae* was represented by seven species; family *Rubiaceae* was represented by six species; and four families (*Amaranthaceae*, *Apiaceae*, *Moraceae,* and *Poaceae*) were represented by five species each. Three families, *Rhamnaceae*, *Vitaceae*, and *Zingiberaceae*, were represented by four species each, and each of the six families (*Araceae*, *Asparagaceae*, *Cucurbitaceae*, *Meliaceae*, *Menispermaceae*, and *Piperaceae*) was represented by three species. The seventeen families (*Anacardiaceae*, *Combretaceae*, *Myrtaceae*, *Phyllanthaceae*, *Rutaceae*, *Verbenaceae*, etc.) were represented by two species each, whereas only one species represented the remaining 52 families.

#### 3.2.2 Habits

Based on their habits, the recorded 232 plant species have been categorized into four groups, among which herbs dominated the list with 99 species (43%) followed by trees with 51 species (22%), shrubs with 43 species (19%), climbers with 36 species (15%), and fruit body with 3 fungal species (1%).

#### 3.2.3 Collection Sources of the Ethnoveterinary Medicinal Plants

Among the 232 documented plant species, 199 species have been collected by the local people from wild sources, which indicates the richness of medicinal flora in the wild and confirms local people’s dependence on wild plant resources. Apart from it, 19 species are procured from commercial sources, and 14 species are grown in the cultivated field in the study area.

#### 3.2.4 Plant Parts Used in Ethnoveterinary Medicine

**FIGURE 5:**
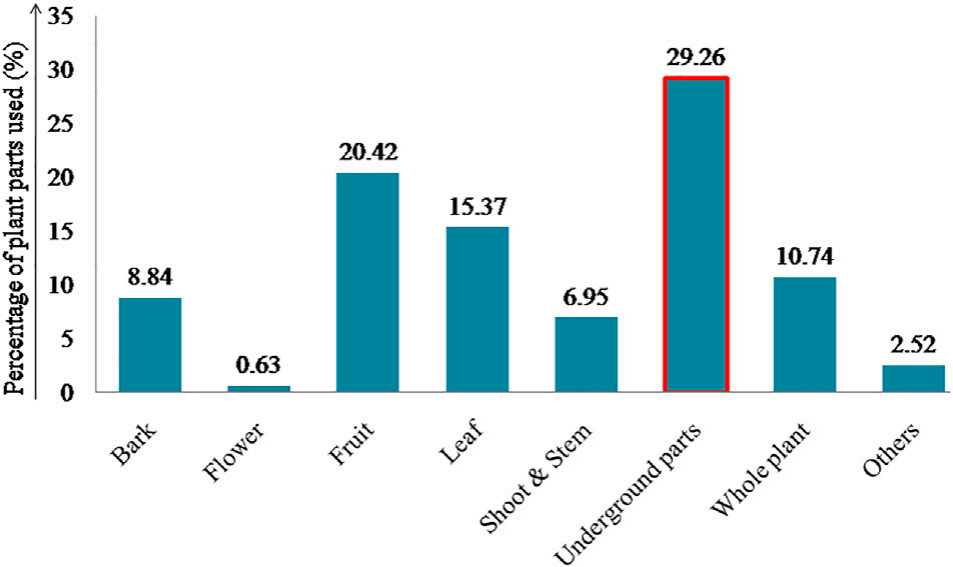
Percentage composition of plant parts used in ethnoveterinary medicine.

For the preparation of ethnoveterinary medicine, plant parts are generally used in their fresh and dried form. Mainly used plant parts recorded here are underground parts (29.26%) such as root, rhizome, bulb, and tuber (Figure 5).

#### 3.2.5 Use of Animal Parts, Earth, Minerals, and Other Substances

Different animal parts, organic and inorganic materials, have also been recorded from the study area. Those substances are used to prepare various remedies. Animal body parts and their products, such as bone, tooth, feather, scale, horn, body fats, honey, and fecal matter, are used along with the plant species in preparation for ethnoveterinary medicine. Earth or soil is used in many folk medicines as one of the ingredients. Soils collected from the mouth of crab hole and termite hill are used by the indigenous people here as an additional ingredient in the preparation of many folk veterinary remedies. Minerals have also been recorded here as important ingredients: rock salt, common salt, vermilion, potassium nitrate, iron sulfate, magnesium sulfate, naphthalene, and “*Sankhachurna*” (powder of Conch shell, a rich source of calcium carbonate). Some organic materials are used here in the form of mustard cake, molasses, jaggery, coconut oil, mustard oil, curd or whey, camphor, sunned rice (*Aatop chal*), particulate rice (*Khud*), and “*Topchini/Chobchini*” (an Ayurvedic product prepared from the dried roots of Chinese Smilax, *Smilax china* L.).

#### 3.2.6 Forms of Remedies Prepared

**FIGURE 6:**
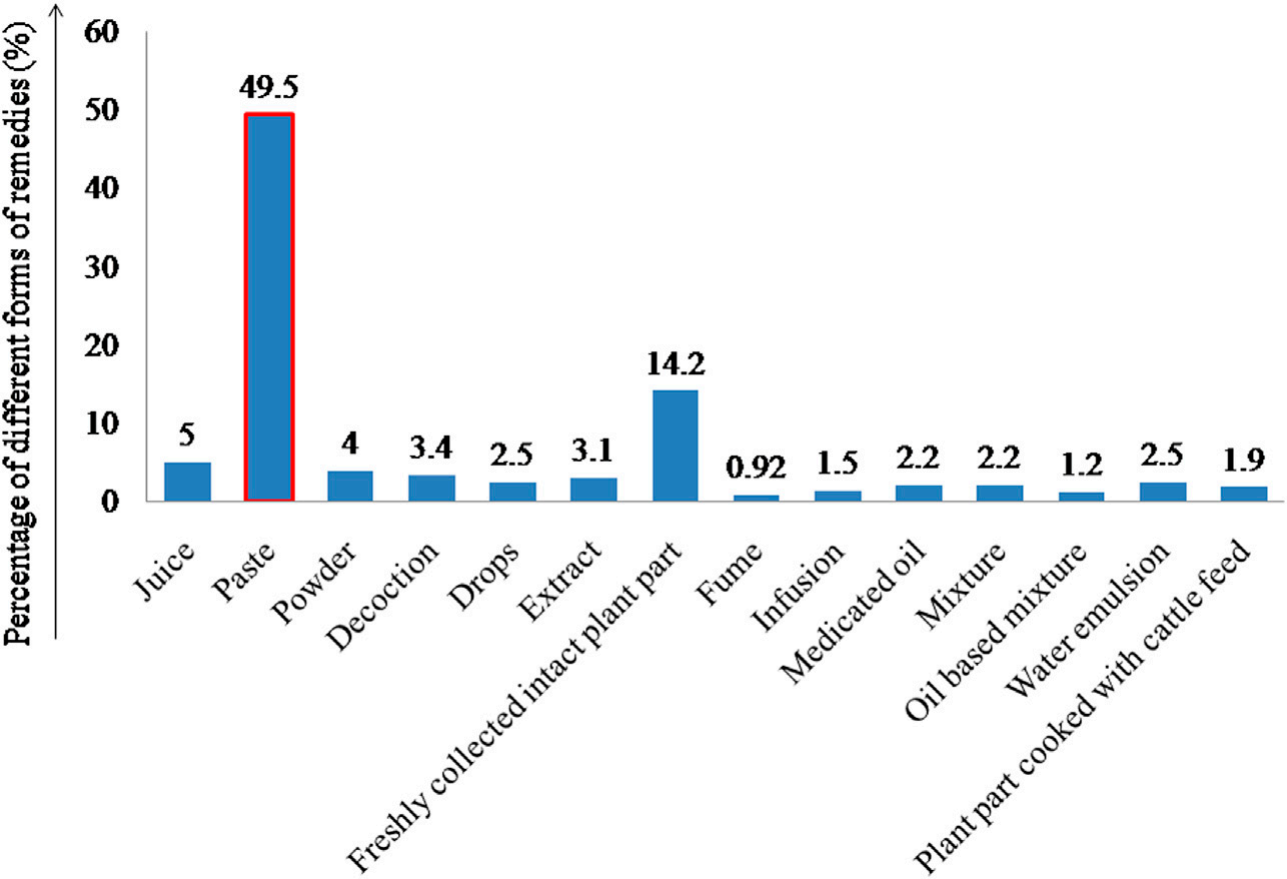
Percentage of the different forms of ethno-remedies.

Folk herbal remedies used for curing veterinary diseases are prepared and administered in various forms to treat several livestock diseases. Fourteen different forms of remedies have been recorded based on their preparation mode. The most predominant form of remedies prepared is paste (Figure 6). The preparation of paste is a widespread form of remedy preparation in different folk and traditional systems of medicine throughout the world.

**FIGURE 7:**
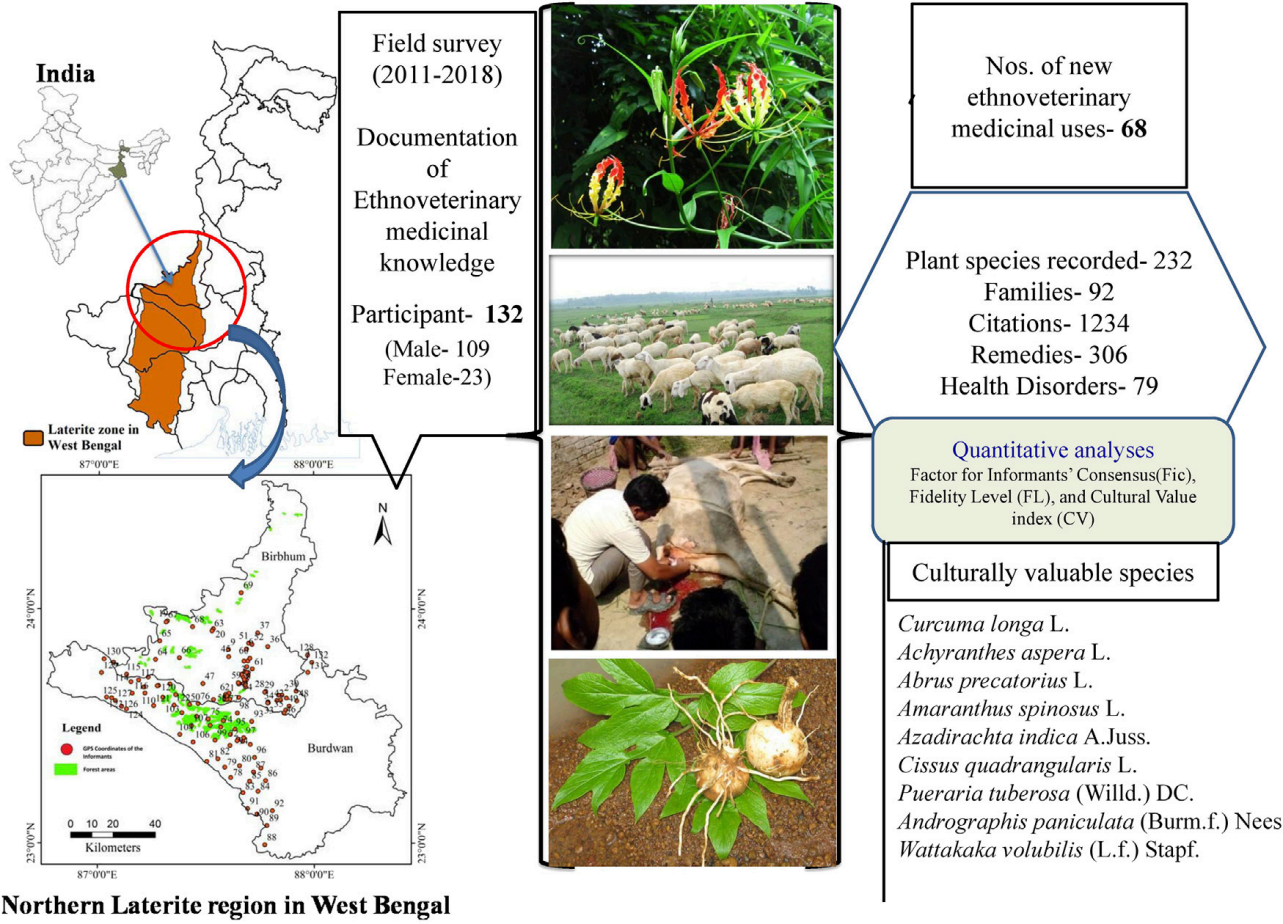
The graphical abstract presents a summary of the current work highlighting its major facts and findings.

#### 3.2.7 Mode of Administration of a Remedy

Two distinct modes of remedy administration have been recorded. The most common route of remedy administration is oral (62%), and remedies are applied in the forms of paste, juice, powder, decoction, and so on. In 32% of cases, folk preparations were administered externally as a poultice, massage, eye drops, fresh intact part of a plant, and so forth. In very minimum cases (2%), the same remedy was administered internally and externally.

Magico-religious belief in the healing of diseases is a deep-rooted integral part of the ethnic cultures. Herein, parts of 11 plants and seven animal species are used in various ways in performing the 19 cases of magico-religious practices to treat 14 diseased conditions of the domesticated animals.

#### 3.2.8 Livestock Diseases and Diagnostic Symptoms

Totally, 79 types of health disorders that prevailed among the veterinary animals were recorded. It has been noticed that animals in this region suffer mostly from the diseases such as gastrointestinal problems, dysentery, diarrhea, fever, and illness due to poisonous effects. Knowledgeable persons of the studied area, especially a “*Go-Vaidya*”—traditional healer of livestock diseases—can easily identify the diseased condition by observing the general appearance and behavior of the mute animals. A list of recorded health conditions of the livestock has been provided along with local names of the diseases and their visible or diagnostic symptoms in Supplementary Table S3.

#### 3.2.9 Enumeration of Folk Veterinary Remedies

Altogether, 232 plant species have been recorded to prepare 306 folk remedies to treat 79 types of livestock diseases in the northern laterite region of West Bengal. Out of 306 recorded folk remedies, 184 remedies are of monoherbal types where only one herbal ingredient is used. The number of polyherbal remedies recorded is 110, and it is prepared using more than one herbal ingredient. The indigenous people administer the remaining 12 remedies out of their magico-religious belief in curing certain livestock diseases.

Among the 232 recorded plant species, ten species have frequently been used as one of the ingredients in a minimal amount, along with the principal ingredient in 110 different polyherbal preparations. Those 10 species are *Piper nigrum* L., *Curcuma longa* L., *Zingiber officinale* Roscoe, *Piper longum* L., *Nigella sativa* L., *Trachyspermum ammi* (L.) Sprague, *Cuminum cyminum* L., *Piper cubeba* L.f., *Ferula assa-foetida* L., and *Allium sativum* L. All the recorded EVMs have been enumerated in a table providing the botanical names of the EVMPs, family, voucher specimen number, common names, their parts used, diseases or health conditions treated, mode of remedy preparation, route of administration, dosages, affected animal, and the number of citations (Table 1).

**TABLE 1:** Enumeration of the ethnoveterinary medicinal formulations recorded from the northern part of the laterite zone in West Bengal (*n* = 306). Symbols denote new reports about EVMPs (♠, new EVMPs reported from India; ♥, new in respect of the diseases cured; ▲, new in respect of remedy preparation modes; ♣, new in respect of the plant parts used).

Sl. No. of Formulations	Scientific name and voucher specimen number	Common name	Family	Parts used	Health conditions treated, mode of remedy preparation, and its administration	Number of citations of each formulation	Animal treated
**A**	**Monoherbal formulations (*n* = 184)**	—	—	—	—	—	—
1	*Abelmoschus moschatus* Medik. SKM12	Musk okra	*Malvaceae*	Seed	Poor health: powdered seeds (100 g) are given with rice bran once a day in the morning for 1 month	3	Cow
2	*Abrus precatorius* L. SKM53	Rosary pea	*Fabaceae*	Seed	1) Loose motion: 3-4 seeds are given orally once a day for 2 days	9	Cow (>2 years)
				Root	2) Mastitis: freshly collected roots of the plant are made into a paste and mixed with the mud of crab hole; applied as a poultice on the affected nipple of mammary gland once a day for 7 days	4	Milch cow
3	*Abutilon hirtum* (Lam.) Sweet ♠ SKM34	Indian mallow	*Malvaceae*	Leaf	Suppurating wound: a handful of leaves are grounded to paste and applied as a poultice on the affected area twice a day for 3 days	2	Pig
4	*Senegalia catechu* (L.f.) P.J.H.Hurter and Mabb. SKM89	Black Catechu	*Fabaceae*	Leaf	Diarrhea: fresh leaves (2–2.5 kg) are fed separately or with fodder twice a day for 3 days	7	Cow and buffalo (>2 years)
5	*Vachellia nilotica* subsp. *indica* (Benth.) Kyal. and Boatwr.SKM02	Prickly acacia	*Fabaceae*	Fruits	1) Opacity of cornea: juice is extracted from unripe fruits (250 g), mixed in 1 L of water, and given orally with a pinch of rock salt twice a day for consecutive 3 days	4	Bullock
				Bark	2) Diarrhea: bark (500 g) extract is given twice a day for 3 days along with a pinch of rock salt	13	Cow and buffalo (>2 years)
6	*Achyranthes aspera* L. SKM85	Prickly chaff flower	*Amaranthaceae*	Root	1) Liver trouble: one mature root (5 cm) is made into a paste with holly water and administered orally once a day for 3 days	5	Cow
				Root	2) Maggot infested wound: root of one mature plant is made into a paste and heated for some time, lukewarm paste applied topically twice a day for 7 days	7	Bullock
7	*Acorus calamus* L. SKM819	Sweet flag	*Acoraceae*	Rhizome	1) Body lice: juice is extracted from fresh rhizome and applied on the whole body once a day for consecutive 3 days	2	Cow
				Rhizome	2) Dyspepsia: dried rhizome powder is mixed with a fine dust of mustard cake. It is fed twice a day for 5–7 days	2	Calf
8	*Aerva javanica* (Burm. f.) Juss. ex Schult. ♠ SKM21	Pillow-weed	*Amaranthaceae*	Leaf	Fractured bone: fresh leaves are made into a paste applied as a poultice on the fractured site; then, the fractured leg is tightly bound with bamboo cheeps and clothes	3	Goat, sheep, dog
9	*Agave americana* L. ♥ SKM23	Century plant	*Asparagaceae*	Leaf	1) Inflammatory swelling of shoulder: the leaves are heated slightly and squeezed to extract out the juice and then administered on the affected area of the shoulder thrice a day for 2-3 days	5	Bullock, buffalo
				Leaf	2) Broken horn: leaf paste along with the common salt is applied as a poultice over broken horn thrice a day for 7 days for quick healing	3	Cow
				Leaf	3) Body ache: one mature leaf made into a paste, applied as a poultice on the affected body parts	5	Cow
10	*Alangium salviifolium* (L.f.) Wangerin SKM145	Sage-leaved alangium	*Cornaceae*	Leaf	1) Liver trouble: juice is taken out from a handful of leaves and given orally once in the morning for consecutive 3 days	2	Donkey
				Bark	2) Opacity of cornea: fresh bark juice is first filtered with the help of fine cloth and given as an eye drop twice a day till the cure	2	Donkey, pony
11	*Albizia procera* (Roxb.) Benth. ♠ SKM512	White Siris	*Fabaceae*	Bark	Diarrhea: decoction is prepared from 250 g bark and given orally once a day for 5 days	2	Cow
12	*Amanita vaginata* var. *alba* Gillet ♠ SKM520	Grisette amanita	*Amanitaceae*	Fruit body	Bloody dysentery: small pieces of 2-3 plants are boiled with particulate rice (*Khud*) and given once in the night for consecutive 3 days	2	Cow, bullock, buffalo
13	*Amaranthus spinosus* L. SKM43	Spiny amaranth	*Amaranthaceae*	Whole plant	1) Delay in parturition: one mature plant is made into paste and given with fodder once a day till the date of delivery	5	Gravid cow
				Root	2) Hemorrhagic septicemia (HS): root of one mature plant is made into paste and fed once a day for 3 days	5	Cow, buffalo
				Whole plant	3) Retention of milk (*Dudh-thunko*): one plant is chopped into small pieces, cooked with particulate rice (*Khud*), and fed once a day for 15–20 days	11	Milch cow
14	*Ampelocissus latifolia* (Roxb.) Planch. SKM213	Wild Grape	*Vitaceae*	Root	1) Snake bite: one piece of mature root (about 10 cm) is made into paste and given orally twice a day for 3 days	2	Cow, bullock, buffalo
				Root	2) Poor lactation: root (2-3 cm long) paste given orally once a day	2	Milch cow
15	*Andrographis paniculata* (Burm.f.) Nees SKM25	Creat	*Acanthaceae*	Aerial part	Foot and mouth disease (FM): dried aerial part made into a paste with honey and given once a day for 7 days	2	Cow
16	*Coleus strobilifer* (Roxb.) A.J.Paton ♠ SKM826	Thick-Leaf Lavender	*Lamiaceae*	Leaf	Cough: a handful of leaves are made into a paste and given orally with old molasses	2	Sheep, goat, cattle
17	*Annona squamosa* L. SKM64	Custard Apple	*Annonaceae*	Leaf	Body lice: leaf juice is applied topically all over the body once a day till the cure	11	Cow, bullock, buffalo
18	*Argemone mexicana* L. SKM28	Mexican prickly poppy	*Papaveraceae*	Leaf	Tick: leaf juice is applied thoroughly all over the body once a day for 3 days to eradicate all the ticks from the body	3	Dog
19	*Argyreia nervosa* (Burm. f.) Bojer SKM832	Elephant Climber	*Convolvulaceae*	Leaf	Miscarriage: freshly collected 9–11 mature leaves are fed daily, starting from 15 days before the expected time of parturition	2	Gravid cow
20	*Asparagus racemosus* Willd. SKM134	Shatavari	*Asparagaceae*	Root	Poor lactation: freshly collected 3–5 pieces of root are made into a paste and given orally on an empty stomach	6	Milch cow
21	*Alstonia scholaris* (L.) R. Br. ♥ SKM918	Devil’s tree	*Apocynaceae*	Leaf	Inflammatory swelling of shoulder: freshly collected seven leaves are made into a paste with common salt and applied as a poultice on the affected shoulder	3	Bullock, buffalo
22	*Allium sativum* L. SKM17	Garlic	*Amaryllidaceae*	Bulb	Maggot infested wound (between hooves and in genital opening): nine cloves of garlic are crushed and boiled in coconut oil; this medicated oil then applied topically onto the infested area	9	Cow, bullock
23	*Artocarpus heterophyllus* Lam. SKM103	Jackfruit	*Moraceae*	Leaf	1) Diarrhea: freshly collected 10–15 leaves are fed once daily till the cure	11	Cow
				Leaf	2) Swelling of dewlap: leaves (about 1 kg) are made into a paste with common salt and applied as poultice throughout the dewlap once in the morning till the cure	2	Bullock
24	*Cajanus goensis* Dalzell. ♠ SKM924	—	*Fabaceae*	Root	Fever: root (about 5 g) is made into a paste and fed with saline water thrice a day for 3 days	2	Sheep
25	*Azadirachta indica* A. Juss. SKM32	Neem	*Meliaceae*	Leaf	1) Body lice: leaf juice applied on the whole body thrice a week	3	Cow
				Seed oil	2) Foot and mouth disease (FM): seed oil applied twice a day onto the affected area till the cure	3	Cow
26	*Bambusa bambos* (L.) Voss. SKM09	Indian thorny bamboo	*Poaceae*	Leaf	Loose motion: fresh leaves (2–2.5 kg) are fed exclusively or along with the cattle feed twice a day for 2-3 days	9	Buffalo, cow
27	*Barleria prionitis* L. SKM418	Dog bush	*Acanthaceae*	Leaf	Wound due to castration: a handful of leaves are crushed into a paste and mixed with mustard oil, applied as a poultice on the wound to stop bleeding and quick healing	5	All types of ruminants
28	*Bauhinia acuminata* L. SKM739	White Bauhinia	*Fabaceae*	Bark	Inflammatory swelling of shoulder: lukewarm bark paste applied as a poultice on the affected area twice a day till the cure	2	Bullock, buffalo
29	*Bombax ceiba* L. SKM44	Silk cotton tree	*Malvaceae*	Bark	1) Diarrhea: dried powder of stem bark is soaked in water overnight and given orally once in the morning	2	Buffalo
				Bark	2) Diarrhea (during pregnancy): about 50 g bark is made into a paste along with cow milk and given orally twice a day for 3 days	2	Gravid cattle
30	*Kalanchoe pinnata* (Lam.) Pers. SKM55	Leaf of life	*Crassulaceae*	Leaf	Retention of urine: leaf paste applied as a poultice on the lower abdomen once a day in the evening for 3 days	2	Goat, sheep
31	*Blumea lacera* (Burm.f.) DC. SKM141	Blumea	*Asteraceae*	Leaf	Retention of the placenta: juice obtained from mature leaves (11 pieces) are given orally twice a day for 3 days	3	Cow
32	*Cajanus scarabaeoides* (L.) Thouars SKM584	Showy pigeonpea	*Fabaceae*	Whole plant	Diarrhea: freshly collected whole plant is chopped finely and fed once in the morning for 5–7 days	7	Goat
33	*Caladium bicolor* (Aiton) Vent. ♠ SKM94	Heart of Jesus	*Araceae*	Corm	Swelling wart: corm made into paste and applied as poultice on the affected area twice a day till the cure	2	Goat
34	*Calotropis gigantea* (L.) W.T.Aiton ▲ SKM73	Giant Milkweed	*Apocynaceae*	Leaf	Rheumatoid arthritis (*Shimola rog*): 14–15 pieces of mature leaves are made into a paste and mixed with 5 g powder of Ammonium chloride (*Nishadal*), fecal matter of a heifer (500 g), and the required amount of soil around the mouth of crab hole. All the ingredients are taken into an earthen pot, heated for a few minutes, and applied all over the paralyzed leg twice a day till the cure	7	Cow, buffalo
35	*Carica papaya* L. SKM19	Papaya	*Caricaceae*	Leaf	Loose motion: fresh leaves (1–1.5 kg) are fed exclusively once a day for 2-3 days	11	Calf
36	*Causonis trifolia* (L.) Mabb. and J.Wen SKM95	Three-Leaved Wild Vine	*Vitaceae*	Whole plant	Swelling of the body due to poisoning (*Sapurey*): the required amount of the whole plant is made into a paste with holly water and applied topically on the whole body only once	11	Cow, bullock, buffalo
37	*Centipeda minima* (L.) A.Braun and Asch. ♠ SKM236	Spreading Sneeze Weed	*Asteraceae*	Whole plant	Rhinorrhoea: freshly prepared juice is slightly heated, and lukewarm juice (two tablespoons) is applied as the nasal drop in each of the nasal openings once in the evening daily till the cure	5	Goat, sheep
38	*Hellenia speciosa* (J.Koenig) S.R.Dutta SKM547	Crepe Ginger	*Costaceae*	Rhizome	Rheumatoid arthritis: rhizome (10–15 g) is made into a paste and fed with molasses once a day for 15 days	3	Cow
39	*Chenopodium album* L. SKM477	-h-b¡-Bl¡ Goosefoot	*Amaranthaceae*	Whole plant	Poor lactation: whole plant (about 500 g) is given once in the morning for 1 month	8	Milch cow
40	*Cissus quadrangularis* L. SKM37	Devil’s backbone	*Vitaceae*	Stem	Fractured bone: stem paste used as a poultice on the affected area and tightly wrapped with clothes remains as it is for at least 1 month	19	All types of small ruminant
41	*Cuscuta reflexa* Roxb. ♥ SKM919	Giant dodder	*Convolvulaceae*	Whole plant	Food poisoning: about 250 g of plant paste is mixed well in 2 L of water and fed immediately	3	Cow, bullock, buffalo
42	*Guilandina bonduc* L. SKM174	Grey nicker	*Fabaceae*	Seed	High fever: powder of 50–150 g of seeds (according to body weight) is given orally once in the morning for 3 days	3	Cow, bullock
43	*Capparis zeylanica* L. SKM39	Ceylon Caper	*Capparaceae*	Stem bark	Bloat: decoction is prepared from 50 g of stem bark and given orally twice a day for 7 days	2	Buffalo (<2 years)
44	*Capparis sepiaria* L. SKM215	Hedge caper	*Capparaceae*	Root	Muscle pain: root extract is given orally once a day for 3 days	2	Bullock
45	*Cardiospermum halicacabum* L. SKM456	Balloon Vine	*Sapindaceae*	Root	Dysentery of a small ruminant: little amount of root is made into a paste and mixed with 500 ml of water, administered orally once a day for 3 days	5	Young goat and sheep (2-3 months of age)
46	*Careya arborea* Roxb. ♥ SKM656	Wild guava	*Lecythidaceae*	Leaf	1) Opacity of cornea: 2-3 drops of leaf juice are applied on the affected eye thrice a day till the cure	2	Bullock
				Bark	2) Dysentery: decoction made from dried bark is given orally twice a day till the cure	2	Cow
				Ripe fruit	3) Constipation: fruit pulp is given orally once a day for 3 days	2	Cow
47	*Carissa spinarum* L. SKM873	Bush plum	*Apocynaceae*	Root	Maggot infested wound: paste prepared from 100 g of dried root is applied as a poultice on the affected area twice a day for 7 days	2	Cow
48	*Casearia tomentosa* Roxb. ♥ SKM693	Toothed Leaf Chilla	*Salicaceae*	Bark	1) Diarrhea: bark extract is given orally twice a day for 3 days	3	Sheep, goat
				Bark or ripen leaf	2) Wound due to castration: bark extract or leaf juice is applied thrice a day till the cure	2	Goat
49	*Baccharoides anthelmintica* (L.) Moench SKM36	Ironweed	*Asteraceae*	Seed	Fever: seed powder is given with molasses after mixing in lukewarm water once a day for consecutive three mornings	3	Bull
50	*Cissampelos pareira* L. SKM54	Velvet Leaf	*Menispermaceae*	Root	Dog or snake bite: root (3–5 cm) extract is given orally as soon as possible	7	Cow
51	*Cocculus hirsutus* (L.) W.Theob. SKM458	Broom creeper	*Menispermaceae*	Leaf	Body lice: leaf juice is applied on the whole body thrice a week	2	Goat
52	*Coix lacryma-jobi* L. SKM163	Job’s tears	*Poaceae*	Root	Diarrhea: root decoction is given orally twice a day for 3 days	2	Cow
53	*Coriandrum sativum* L. SKM78	Coriander	*Apiaceae*	Seed	1) Haematuria: an infusion made from seed dust is given in the early morning once a day for consecutive 10–15 days	5	Cow
				Whole plant	2) Mastitis: whole plant made into a paste with holly water and applied on the stiff and painful tits twice a day till the cure	2	Milch cow and buffalo
54	*Crinum asiaticum* L. SKM179	Seashore Lily	*Amaryllidaceae*	Bulb	Swelling warts: poultice of tuber paste is applied on the swollen area twice a day for consecutive 7 days	5	Buffalo
55	Croton persimilis Müll. Arg. ♠ SKM189	Croton Tree	*Euphorbiaceae*	Root	Poor lactation: paste prepared from mature root (10 cm) is given orally once in the evening for 15–20 days	2	Milch cow
56	*Coccinia grandis* (L.) Voigt SKM191	Ivy gourd	*Cucurbitaceae*	Leaf	1) Whitening and watering of eyes: 2-3 drops of leaf juice is applied on the affected eye once a day for 7–10 days	5	Cow, buffalo
				Fruiting shoot	2) Poor health: finely chopped plant parts are fed once a day for 7 days	3	Buffalo, bullock
57	*Curculigo orchioides* Gaertn. SKM62	Golden eye-grass	*Hypoxidaceae*	Root	1) Foot and mouth disease (FM): dried root powder is mixed with rice bran in 1:10 ratio and given orally once a day at first morning for 5–7 days	2	Bullock
				Root	(ii) Poisonous bite: root (5–7 cm) made into a paste with holly water and fed quickly	2	Cow
58	*Curcuma longa* L. SKM20	Turmeric	*Zingiberaceae*	Rhizome	Loosening of teeth (*Kalasashru*): freshly collected rhizome is made into a paste and mixed with scale-ash of the Chital fish (*Chitala chitala*) and rock salt in 2:1:2 ratio; the entire mixture is then stirred well in mustard oil and applied at the base of the loosened teeth, wrapped with a piece of cotton and then a red hot ion rod is put on it. This practice is done once a day for successive 3 days	2	Bullock, buffalo
59	*Volkameria inermis* L. SKM564	The glory bower	*Lamiaceae*	Leaf	Body lice: leaf juice is applied all over the body on alternative days for a week	3	Cow, bullock, goat
60	*Clerodendrum infortunatum* L. SKM08	Hill glory bower	*Lamiaceae*	Leaf	Body lice: leaf juice is administered throughout the body once a day for 2-3 days followed by a thorough bath	7	Cow, bullock, goat
61	*Cleome gynandra* L. SKM991	Cat’s whiskers	*Cleomaceae*	Whole plant	Rheumatoid arthritis (*Shimola-rog*): the whole plant is made into a paste with common salt and fecal matter of black goat, applied topically on the affected area once a day for 9-10 days	2	Bullock
62	*Colocasia esculenta* (L.) Schott SKM76	Taro	*Araceae*	Corm	Tumor: freshly collected corm is made into a paste along with common salt and applied as poultice once a day for 5–7 days	3	Sheep
63	*Cotula anthemoides* L. ♠ SKM266	Buttonweed	*Asteraceae*	Whole plant	Watering of eyes: infusion is used to wash the infected eyes	2	Cow
64	*Crotalaria quinquefolia* L. SKM353	Five Leaf Rattlepod	Fabaceae	Whole plant	A sudden decrease in milk production: the whole plant is used as cattle feed once a day for 15 days	3	Milch cow
65	*Cyanotis tuberosa* (Roxb.) Schult. and Schult.f. SKM11	Sahyadri Dew-Grass	*Commelinaceae*	Root	Fever: root paste is given orally along with rice bran once a day for consecutive 3–5 days	4	Heifer
66	*Datura stramonium* L. SKM114	Thorn apple	*Solanaceae*	Leaf and fruit	Food poisoning: leaves (8–10 pieces) or fruits (1 or 2) are made into a paste and administered orally once a day for at least 3 days	4	Cow
67	*Dendrophthoe falcata* (L.f.) Ettingsh. SKM92	Long-leaved Mistletoe	*Loranthaceae*	Leaf	Prolapsed uterus (*Bhnaral berono*): leaf extract is used as a surface disinfectant to immediately wash the oozed out uterus and then replacement in its original position is done	4	Cow
68	*Dendrolobium triangulare* (Retz.) Schindl. SKM678	Triangular Horse Bush	*Fabaceae*	Leaf	Whitening of eyes: one tablespoon of leaf juice is applied dropwise on the affected eye	2	Calf
69	*Dioscorea bulbifera* L. SKM268	Air yam	*Dioscoreaceae*	Tuber	Mastitis: 250–100 gm tuber is sliced and given with cattle feed once a day for 7 days	3	Milch cow
70	*Dillenia pentagyna* Roxb. SKM265	Dog Teak	*Dilleniaceae*	Bark	Helminthiasis: bark powder is given orally once a day at nighttime for 7 days	2	Sheep
71	*Wattakaka volubilis* (L.f.) Stapf. SKM182	Green Milkweed Climber	*Apocynaceae*	Leaf	1) Swelling of throat: 21 leaves are made into a paste along with common salt and applied as a poultice on the affected area for consecutive 3 days	3	Bullock
				Stem	2) Discontinuity in urination/urinary incontinence: freshly collected stems are boiled with particulate rice (*Khud*) and given orally once a day till the cure	9	Cow
72	*Echinops echinatus* Roxb. ♣ SKM421	Indian globe thistle	*Asteraceae*	Tender shoot	1) Infertility: freshly collected 2-3 tender twigs are made into a paste and fed along with paddy straw after the onset of the normal heat period	2	Cow
				Whole plant	2) Sore on shoulder: 250 g of freshly collected plants is made into a paste and applied as a poultice on the affected shoulder twice a day till the cure	2	Bullock, buffalo
73	*Enydra fluctuans* DC. SKM422	Buffalo Spinach	*Asteraceae*	Whole plant	Constipation: freshly collected plants (1.5–2 kg) are fed twice a day to get relief from constipation	7	Goat
74	*Euphorbia antiquorum* L. ♥ SKM302	Triangular Spurge	*Euphorbiaceae*	Latex	Opacity of cornea: two drops of fresh latex is given as eye drop on the affected eye once a day for 3 consecutive mornings	3	Bullock
75	*Euphorbia fusiformis* Buch.-Ham. ex D. Don ♥ SKM93	Pillpod spurge	*Euphorbiaceae*	Tuberous root	Poor lactation: fresh tuber (100–150 g) is sliced and given with rice gruel or 50 g tuber powder is fed along with rice bran once in the evening for at least 15 days	4	Milch cow
76	*Euphorbia hirta* L. SKM147	Hairy Spurge	*Euphorbiaceae*	Latex	Mastitis: freshly collected latex is applied topically thrice a day till the cure	2	Milch cow
77	*Euphorbia neriifolia* L. SKM229	Indian Spurge Tree	*Euphorbiaceae*	Latex and stem bark	1) Broken horn: latex is applied on the base of the broken horn, and a poultice is made with bark, which is applied uniformly over it and then tied with cloth and left as it is for 21 days	2	Bullock
				Leaves	2) Mastitis: leaves are made into a paste along with sunned rice (*Atop-chal*) and applied as a poultice on the affected tits	3	Milch cow
78	*Ficus benghalensis* L. SKM06	Indian banyan	*Moraceae*	Prop root	Dysentery: the soft, red, apical parts of prop roots (12–15 cm long) are given exclusively or along with the cattle feed, twice a day for 2-3 days. Dose: cow and buffalo—6 to 7 pieces, goat and sheep—2 to 3 pieces	9	Cow, buffalo, goat, sheep
79	*Ficus religiosa* L. SKM82	Sacred Fig Tree	*Moraceae*	Leaf	Foot and mouth disease (FM) (*Pankui*): 5 or 6 fresh leaves are made sacred by some religious ritual (i.e., name of the lord “*Arjuna*” is written 12 times on each leaf) and fed twice a day for 3 days	3	Cow
80	*Ficus racemosa* L. SKM109	Cluster fig	*Moraceae*	Fruit	Constipation: sliced mature fruit is given orally	3	Bullock
81	*Gardenia latifolia* Aiton SKM1113	Indian Boxwood	*Rubiaceae*	Bark	Wound: bark decoction applied on the wound twice a day till cured	2	Sheep, goat, cow
82	*Gloriosa superba* L. SKM908	Flame lily	*Colchicaceae*	Tuber	1) Prolapsed uterus: extract of the tuber is applied externally to wash the oozed out uterus	7	Cow
			—	Tuber	2) Abscess in liver: tuber paste is given with molasses once a day for at least 1 month	2	Bullock
83	*Adina cordifolia* (Roxb.) Brandis SKM912	Heart-leaf adina	*Rubiaceae*	Tender leaf	Opacity of cornea: leaf juice is applied as eye drop once in the morning till cured	2	Sheep
84	*Helicteres isora* L. SKM817	Indian screw tree	*Malvaceae*	Fruit	1) Bloody dysentery: dried fruits (3–5 pieces) are made into a paste with a pinch of black salt and given twice a day for 3 days	3	Cow
				Leaf	2) Poisonous bite: leaf paste is used as a poultice on the biting site	7	Goat
85	*Strobilanthes hirta* (Vahl) Blume SKM791	Red Ivy	*Acanthaceae*	Whole plant	Haematuria: freshly collected plants (250 g) are made into a paste, mixed in 1 L of water, and fed the entire preparation with the help of a bottle	7	Cow
86	*Hibiscus cannabinus* L. SKM1133	Deccan hemp	*Malvaceae*	Leaf	Constipation: 250–300 g of fresh leaves are given orally once a day for 3 days	2	Bullock
87	*Hydrolea zeylanica* (L.) Vahl ♠ SKM549	Ceylon Hydrolea	*Hydroleaceae*	Whole plant	Wound: a bunch of whole plant is made into a paste and applied topically once daily till cured	3	Bullock
88	*Mesosphaerum suaveolens* (L.) Kuntze ♥ SKM128	Mint Bush	*Lamiaceae*	Leaf	Fresh cuts and wounds: leaf juice is applied as a hemostatic agent	4	Bullock, buffalo
89	*Ipomoea cairica* (L.) Sweet ♠ SKM239	Messina Creeper	*Convolvulaceae*	Tuber	Helminthosis (with stomachache): tuber (100–150 g) made into a paste with holly water and given with fodder once a day for 3 days	2	Cow
90	*Ipomoea carnea* Jacq. SKM137	Bush Morning Glory	*Convolvulaceae*	Soft stem	Poor lactation: small pieces are boiled with particulate rice and given at night once a day for 5–7 days	5	Milch cow
91	*Ipomoea obscura* (Linn.) Ker.-Gowl. ♠ SKM457	Obscure Morning Glory	*Convolvulaceae*	Leaf	Broken horn: leaf paste is mixed with coconut oil and heated slightly. This lukewarm paste is then applied as a poultice at the base of the broken area and tightly wrapped with a cloth	2	Goat
92	*Jatropha nana* Dalzell and A.Gibson ♠ SKM151	Dwarf Jatropha	*Euphorbiaceae*	Tuberous root	Retention of milk: dried root powder is given orally once a day for 10–15 days (sometimes fed with finely pounded mustard cake)	3	Milch cow
93	*Justicia adhatoda* L. SKM184	Malabar nut	*Acanthaceae*	Leaf	Body swelling due to cold (*Jol-sannipat*): leaf paste is heated slightly and applied on the whole body surface twice a day for 3 days	3	Cow
94	*Justicia gendarussa* Burm.f. ♥ SKM339	Willow-leaved Justicia	*Acanthaceae*	Root	Helminthosis: root (5–7 g) paste is given with lukewarm water once at night for 7 days	3	Cow
95	*Jatropha gossypiifolia* L. SKM192	Bellyache bush	*Euphorbiaceae*	Seed	Constipation: seed paste is given orally to treat constipation	2	Goat
96	*Lawsonia inermis* L. SKM657	Henna tree	*Lythraceae*	Leaf	Foot and mouth disease (FM): leaf paste is applied topically on the affected area twice a day till the cure	5	Cow
97	*Leonotis nepetifolia* (L.) R.Br. SKM638	Christmas candlestick	*Lamiaceae*	Root	Mastitis: root paste is applied as a poultice on the whole udder and tits twice a day for 3 days	2	Milch cow
98	*Lippia javanica* (Burm.f.) Spreng. SKM676	Lemon bush	*Verbenaceae*	Whole plant	Tick: freshly collected plant is made into a paste and applied topically all over the body once a day for 3 days	2	Cow
99	*Linum usitatissimum* L	jp-e Linseed	*Linaceae*	Seed oil	Constipation: oil is extracted from 750 g to 1 kg of seeds and given half of it at a time orally twice a day for 3 days	7	Bullock, buffalo
100	*Litsea glutinosa* (Lour.) C. B. Rob. ♥ SKM722	Bolly beech	*Lauraceae*	Bark	1) Loose motion: 1 L of water emulsion is prepared from 200 g of freshly made bark paste and given twice a day for 3 days	4	Cow, buffalo
				Bark	2) Dislocation of joints: bark is made into a paste and heated slightly. After application of this lukewarm paste on the affected area, a tight bandage of bamboo stick is provided	6	Cow
101	*Ludwigia adscendens* (L.) H.Hara ♠ SKM834	Water Primrose	*Onagraceae*	Whole plant	Infertility: freshly collected plants (size must be 1/3 the body length of the cattle treated) are chopped into small pieces and fed along with paddy straw once a day for a month, starting from the onset of the normal heat period	2	Heifer
102	*Luffa acutangula* (L.) Roxb	Ridged Gourd	*Cucurbitaceae*	Dried fruit fiber	Cold, cough, and watering of nose: affected animals are separately kept in a room of cowshed; dried fruit fibers are kept inside it and fired to produce smoke as a remedy	11	Bullock
103	*Luffa aegyptiaca* Mill. SKM161	Sponge gourd	*Cucurbitaceae*	Dried fruit fiber	Cold, cough, and watering of the nose: smoke is produced by burning the dried fruit fibers in a separate room of a cowshed, and affected animals are kept there for some time for quick healing	7	Bullock
104	*Lycoperdon perlatum* Pers. SKM91	Puffball	*Agaricaceae*	Spore	Ulcerated wound: spore dust is applied topically on the affected part once a day for 5 days	5	Sheep
105	*Manilkara hexandra* (Roxb.) Dubard SKM341	Ceylon wood	*Sapotaceae*	Stem bark	Tonsillitis: bark is made into a paste along with the mud of crab hole and warmed slightly; poultice: twice a day till the cure (applied on the outer side of lower jaws)	2	Bullock
106	*Martynia annua* L. SKM441	Tiger’s claw	*Martyniaceae*	Leaf	Wound: leaf decoction is applied topically to disinfect the wound	2	Goat
107	*Mimosa pudica* L. SKM641	Touch-me-not	*Fabaceae*	Root	Maggot infested wound: root paste is applied topically twice a day for 5 days	7	Cow
108	*Mitragyna parvifolia* (Roxb.) Korth. SKM761	Kaim	*Rubiaceae*	Bark	Septic wound: bark juice applied topically to wash the wound	2	Sheep, goat, pig
109	*Moringa oleifera* Lam. SKM846	Drumstick tree	*Moringaceae*	Root	Food poisoning: juice is made from 250 g of root and mixed well in 2 L of water and given immediately	5	Cow
110	*Musa paradisiaca* L. ♥ SKM31	Banana	*Musaceae*	Corm	1) Haematuria: the corm of a post-fruiting plant is collected and sliced into several small pieces and then kept in cold water overnight. The pieces along with that water is administered twice a day for 2-3 days (for immature —once a day).	4	Cow
				Leaf	2) Loose motion: 1 or 2 leaves are fed solely twice a day for 2-3 days	8	Cow
111	*Nerium oleander* L. SKM67	Oleander rose-bay	*Apocynaceae*	Leaf	Mastitis: leaf paste is used as a poultice on the affected tits twice a day till cured	2	Milch cow
112	*Nicotiana rustica* L. ▲♥	Aztec tobacco	*Solanaceae*	Leaf	Maggot infested wound: 10 g of leaf powder of tobacco and 100 g powder of “*Sankhachurna*” are mixed with mustard oil and administered on the wound many times a day for a few days	3	Bullock, buffalo
113	*Nigella sativa* L	Black cumin	*Ranunculaceae*	Seed	Mastitis: seeds are boiled with particulate rice (“*Khud*”) grain and fed daily once in a day	7	Milch cow
114	*Nymphaea nouchali* Burm.f. SKM752	Red Water Lily	*Nymphaeaceae*	Rhizome	Stop mastication: rhizome paste is given orally	2	Bullock
115	*Oroxylum indicum* (L.) Kurz ♣ SKM519	Indian trumpet tree	*Bignoniaceae*	Flower	Mastitis: flower paste is applied as a poultice on cracked nipples twice a day for 7 days	2	Milch cow and buffalo
116	*Papaver somniferum* L. ♥	Opium poppy	*Papaveraceae*	Fruit coat	Bloody dysentery: fruit coat (200 g) is soaked in 2 L of water overnight and then boiled and condensed to 500 ml. The boiled fruit coats are squashed and strained. Whole soup is then administered of a dose of 25–30 ml, 3-4 times a day for a few days	5	Cow
117	*Phoenix acaulis* Roxb. ♠ SKM205	Dwarf date palm	*Arecaceae*	Tender Leaf	1) Retention of milk (post-parturition): freshly collected tender leaves are chopped finely and fed once a day for 10–15 days	4	Cow
				Soft root	2) Dystocia (difficulty in parturition): soft roots (50 g) are made into a paste and fed along with rice gruel once a day for 10 days till the expected date of parturition to avoid any difficulty in it	2	Cow
118	*Phyla nodiflora* (L.) Greene SKM362	Frog fruit	*Verbenaceae*	Whole plant	Dyspepsia of calf: freshly collected plants are chopped finely and given orally once a day for 15–20 days	2	Heifer
119	*Polygala arvensis* Willd. ♥ SKM108	Field Milkwort	*Polygalaceae*	Whole plant	Nervine disease/listeriosis (*Gai-ghuro*): a bunch of whole plants is chopped finely and given with paddy straw once in the morning for 9 days	2	Cow
120	*Portulaca oleracea* L. SKM720	Common purslane	*Portulacaceae*	Whole plant	Mastitis: plant paste is given orally once a day for 7 days	2	Goat
121	*Pueraria tuberosa* (Willd.) DC. ♥ SKM188	Indian kudzu	*Fabaceae*	Tuber	1) Helminthosis: sliced pieces of tuber are fed along with paddy straw or with rice gruel once in the morning for three consecutive days	17	Cow, buffalo
				Tuber	2) Poor lactation: sliced pieces of tuber (fresh or dried form) are given orally once a day for 15 days	3	Cow
				Tuber	3) General weakness: sliced pieces are soaked overnight in rice gruel and fed the whole thing in the next morning	7	Milch cow and buffalo
122	*Psidium guajava* L. SKM370	Guava	*Myrtaceae*	Bark	Cuts and wounds: bark decoction is applied on the affected area as a disinfectant	3	Cow, bullock, buffalo, goat, sheep
123	*Rivea hypocrateriformis* Choisy SKM77	Common Night Glory	*Convolvulaceae*	Soft stem	Fractured bone: plant paste is applied as a poultice on the affected area and wrapped tightly with bamboo sticks and left as it is for 1 month	4	Bullock
124	*Santalum album* L. SKM311	Sandalwood	*Santalaceae*	Bark	Fever: paste of bark (100–150 g) is given orally once a day for 2 days	3	Cow
125	*Scoparia dulcis* L. ♥ SKM10	Sweet-broom	*Plantaginaceae*	Whole plant	Retention of urine: water emulsion is prepared in 1 L of water with 100 g of plant paste and drench once a day for 7 days	3	Sheep, goat
126	*Schoenoplectiella articulata* (L.) Lye ♥ SKM313	Jointed Sedge	*Cyperaceae*	Seed	Wound due to castration: seed dust is boiled in coconut oil and applied topically on the affected area thrice a day for 3 days	2	Goat
127	*Semecarpus anacardium* L.f. SKM513	Marking nut	*Anacardiaceae*	Seed	Liver trouble: 2 or 3 seeds are fed once a week to strengthen liver function	3	Cow
128	*Senna occidentalis* (L.) Link SKM105	Antbush	*Fabaceae*	Root	Diarrhea: root (5–7 g) paste is given orally twice a day for 5 days	5	Sheep
129	*Dracaena angolensis* (Welw. ex Carrière) Byng & Christenh. SKM110	Spear Sansevieria	*Asparagaceae*	Root	Swelling wart: root paste is applied twice a day on the affected area till the cure	2	Goat
130	*Sesamum indicum* L.	Sesame	*Pedaliaceae*	Seed	Retention of milk: seed (200 g) paste is soaked in water along with mustard cake for 6–8 h and given orally once a day on alternate days for a week	6	Milch buffalo
131	*Seseli diffusum* (Roxb. ex Sm.) Santapau & Wagh ♠ SKM788	Indian celery	*Apiaceae*	Whole plant	Urinary incontinence: a bunch of freshly collected plants in its fruiting stage is given orally once a day till cured	2	Cow
132	*Smilax ovalifolia* Roxb. ex D.Don SKM201	Kumarika	*Smilacaceae*	Root	1) Body ache: root paste is mixed with mud of termite hill and applied on the affected area	3	Bullock
				Leaf	2) Indigestion: fresh leaves are chopped and fed once in the morning for 15 days	2	Sheep
133	*Solanum violaceum* Ortega SKM119	Indian Nightshade	*Solanaceae*	Whole plant	Poisoning of grazing animal: one plant is slightly toasted first and then made into small pieces and fed immediately along with the cattle feed	5	Cow, bullock, buffalo, horse
134	*Solanum sisymbriifolium* Lam. SKM29	Sticky nightshade	*Solanaceae*	Whole plant	Infectious disease: one mature plant in its fruiting stage is collected, ground finely, and given thrice a week	6	Cow
135	*Solanum torvum* Sw. ♥ SKM601	Turkey Berry	*Solanaceae*	Fruit	Bloat: 9–11 pieces of ripe fruits are given once a day for 3 days	2	Sheep, goat
136	*Soymida febrifuga* (Roxb.) A. Juss. SKM99	Indian Redwood	*Meliaceae*	Bark	1) Dysentery: juice is prepared from freshly collected bark and given twice a day for 3 days	7	Cow
				Leaf	2) Retention of placenta: nine leaves are fed in the first morning for 2 days	3	Cow
137	*Sphaeranthus indicus* L. SKM901	East Indian Globe Thistle	*Asteraceae*	Whole plant	Wound: plant paste is applied as a poultice on the affected area	5	Bullock
138	*Strychnos nux-vomica* L. SKM402	Nux vomica	*Loganiaceae*	Bark	Bloody dysentery: 5–10 g stem bark is made into a paste with common salt and fed along with cattle fed once a day till cured	4	Cow
139	*Swertia chirayita* (Roxb.) H.Karst	Chirayata	*Gentianaceae*	Whole plant	Constipation: certain amount of dried plant along with rock salt is made into powder and given twice a day for several days	6	Cow, buffalo
140	*Tamarindus indica* L. SKM211	Tamarind	*Fabaceae*	Fruit pulp	Food poisoning: fruit pulp (250 g) is mixed with 2 L of water and given immediately	5	Cow
141	*Tamilnadia uliginosa* (Retz.) Tirveng & Sastre SKM1138	Divine Jasmine	*Rubiaceae*	Fruit	Dysentery: one teaspoon full of dust of mature fruit is given once in the morning for 3 days	2	Goat
142	*Termitomyces heimii* Natarajan ♠ SKM124	Termite mound mushroom	*Lyophyllaceae*	Whole fruit body	General weakness: dust is given with rice bran once a day thrice a week for 1 month	2	Bullock, buffalo
143	*Tinospora sinensis* (Lour.) Merr. SKM251	Heart-leaved moonseed	*Menispermaceae*	Stem	Poor lactation: mature stem (500 g) is boiled with particulate rice and fed daily for 10–15 days	5	Milch cow
144	*Trianthema portulacastrum* L. SKM909	Desert horsepurslane	*Aizoaceae*	Leaf	Opacity of cornea: leaf juice is applied on the affected eye till cured	2	Goat, sheep
145	*Tribulus terrestris* L. ♥ SKM710	Puncture Vine	*Zygophyllaceae*	Leaf	Bloat: fresh leaves are fed with a pinch of rock salt twice a day for 3 days	3	Sheep
146	*Typhonium trilobatum* (L.) Schott ♥ SKM811	Bengal Arum	*Araceae*	Tuber	Swelling wart: tuber paste applied on the affected area twice a day for 5 days	4	Goat
147	*Urena lobata* L. SKM304	Caesarweed	*Malvaceae*	Root	Body lice: juice made from fresh root is applied on the whole body once a day for consecutive 3 days	3	Cow
148	*Uraria lagopoides* (L.) DC. ♠ SKM612	Hare Foot Uraria	*Fabaceae*	Whole plant	Diarrhea of small ruminant: whole plant is chopped finely and fed once in the evening for 3–5 days	2	Goat
149	*Vanda tessellata* (Roxb.) Hook. ex G.Don SKM451	Grey orchid	*Orchidaceae*	Aerial root	Swelling wart: root made into a paste and applied topically on the affected area once a day for 7 days	2	Goat
150	Holarrhena pubescens Wall. ex G.Don SKM03	Bitter Oleander	*Apocynaceae*	Seed	Fever with stomach problems and pulmonary congestion: seeds (*Indrajob*) are made into a paste along with potassium nitrate (4:1) and administered twice a day for 5 days	2	Cow
151	*Xanthium strumarium* L. SKM301	Rough cocklebur	*Asteraceae*	Leaf	Retention of urine: leaves are made into a paste along with common salt and applied as a poultice on the lower abdomen once a day for 5–7 days	3	Cow
152	*Ziziphus nummularia* (Burm.f.) Wight and Arn. ♥ SKM48	Wild jujube	*Rhamnaceae*	Leaf	Rhinorrhoea: dried leaves are fired to get smoke in the cowshed where the affected cow is tied for a few hours	9	Cow
153	*Ziziphus oenoplia* (L.) Mill. SKM57	Jackal jujube	*Rhamnaceae*	Bark	Suppurating wound: freshly collected bark (250 g) is soaked in water overnight, and the next morning, the wound is washed with this water. This practice is continued daily till cured	2	Bullock
**B**	**Polyherbal formulations (*n* = 110)**	—	—	—	—	—	—
154	*Abrus precatorius* L.	—	—	Seed	Loose motion: 4-5 seeds of *A. precatorius* are given with bamboo leaves once a day for 2-3 days	7	Goat
	*Bambusa bambos* (L.) Voss.	—	—	Leaf			
155	*Abrus precatorius* L.▲	—	—	Seed	Diarrhea and dysentery: 4-5 *Abrus* seeds are made into paste along with 100 g bamboo leaves and little amount of feather of *Coracias benghalensis* L. (Indian roller, “Nilkantha”), applied orally once a day for 3 days	16	Cow
	*Bambusa bambos* (L.) Voss.	—	—	Leaf			
156	*Leonotis nepetifolia* (L.) R.Br. ▲	—	—	Root	Mastitis: roots of *L. nepetifolia* and *A. precatorius* (2:1) is made into a paste and applied as a poultice on the mammary gland twice a day for 3 days	2	Milch cow
	*Abrus precatorius* L.	—	—	Root			
157	*Abrus precatorius* L.	—	—	Root	Cold and cough: 5 g root of *A. precatorius* and 21 grains of black pepper are made into a paste and given once a day in empty stomach in the morning for consecutive 3 days	2	Goat, sheep
	*Piper nigrum* L.	Black pepper	*Piperaceae*	Fruit			
158	*Senegalia catechu* (L.f.) P.J.H.Hurter and Mabb.	—	—	Latex	Loose motion: dried latex of catechu (200 g), chalk (1 kg), and iron sulfate (200 g) are ground into powder separately and then mixed with root powder of *Piper cubeba* (200 g) and administered twice a day for 2-3 days. Simultaneously with this administration, the paste of sunned rice is given once a day. Dose: for goat—5 to 10 g, for cow—50 g or more and buffalo-100 g or more	2	Bullock, buffalo
	*Piper cubeba* L.f.	Java pepper	*Piperaceae*	Root			
159	*Aegle marmelos* (L.) Corrêa SKM101	Stone apple	*Rutaceae*	Bark	Poor health: a decoction is prepared from bark of *A. marmelos* (250 g), bark of *V. nilotica* subsp. *indica* (250 g), bark of *A. salvifolium* (150 g), bark of *A. nilotica* (150 g), rhizome of *N.* (100 g), and and whole plant of *T. natans* (100 g) or tuber of *P. tuberosa* (200 g). This preparation is used as a tonic and given once a day for 7–10 days	2	Cow, bullock
	*Vachellia nilotica* subsp. *indica* (Benth.) Kyal. and Boatwr.	—	—	Bark			
	*Alangium salviifolium* (L.f.) Wangerin ♥ SKM145	—	—	Bark			
	*Azadirachta indica* A. Juss.	—	—	Bark			
	*Nelumbo nucifera* Gaertn. SKM152	Indian lotus	*Nelumbonaceae*	Rhizome			
	*Trapa natans* L. SKM306	Water nut	*Lythraceae*	Whole plant			
	*Pueraria tuberosa* (Willd.) DC.	—	—	Tuber			
160	*Achyranthes aspera* L. ▲	—	—	Root	Fever of gravid cow: roots of *Achyranthes*—2 to 3 pieces (5–7 cm long), 2 spoonfuls of seeds of *Nigella sativa* are ground together into a paste and fed along with mucilaginous extract of *Aloe vera* leaf (locally known as “*Musabbar*”) twice a day for 2-3 days	11	Gravid cow
	*Nigella sativa* L.	—	—	Seed			
	*Aloe vera* (L.) Burm.f. SKM40	Aloe	*Asphodelaceae*	Leaf			
161	*Achyranthes aspera* L.	—	—	Root	Fever: root of one mature *A. aspera* plant and 9 grains of black pepper are made into a paste and mixed with water to be fed twice a day for 5–7 days	13	Bullock
	*Piper nigrum* L.	—	—	Fruit			
162	*Solanum glaucophyllum* Desf. SKM193	Waxyleaf Nightshade	*Solanaceae*	Stem	Fever: three pieces of dried stem of *S. glaucophyllum* (5 cm), whole plant of *S. dulcis* (100 g), and root of *A. aspera* (50 g) are made into a paste and given orally twice a day for 7 days	3	Cow
	*Scoparia dulcis* L.	—	—	Whole plant			
	*Achyranthes aspera* L.	—	—	Root			
163	*Achyranthes aspera* L.	—	—	Root	Fever of heifer (immature cow): roots of *Achyranthes* and *S. surattense* (3:1) are made into a paste and given orally once a day for 3 days	5	Heifer
	*Solanum virginianum* L. SKM115	Surattense nightshade	*Solanaceae*	Root			
164	*Pleurolobus gangeticus* (L.) J.St.-Hil. ex H.Ohashi and K.Ohashi ♥ SKM171	Sal Leaved Desmodium	*Fabaceae*	Whole plant	Hemorrhagic septicemia (HS): one entire plant of *Pleurolobus*, 5–7 g of *Achyranthes* roots, and 21 grains of black pepper are made into a paste together, fed along with old molasses once a day till the cure	9	Cow
	*Achyranthes aspera* L.	—	—	Root			
	*Piper nigrum* L.	—	—	Fruit			
165	*Adiantum philippense* subsp. *philippense* ♠ SKM90	Maidenhair fern	*Pteridaceae*	Whole plant	Fever: a handful of freshly collected whole plant of *A. philippense* subsp. *philippense* is given orally along with black pepper once a day till the cure	3	Goat, sheep
	*Piper nigrum* L.	—	—	Fruit			
166	*Aerva javanica* (Burm. f.) Juss. ex Schult	—	—	Shoot	Lameness in the hind leg due to stiffness of medial collateral ligament (“*Shir-taan*”): bulbous root of *E. explanata*, tender shoot of *A. javanica*, aerial part of *A. paniculata*, soil of crab hole, and little amount of camphor are ground together and mixed well, heated slightly, and applied as a poultice on the affected leg twice a day for 5–7 days	3	Bullock
	*Eulophia explanata* Lindl. ♠ SKM200	Flattened Eulophia	*Orchidaceae*	Bulb			
	*Andrographis paniculata* (Burm.f.) Nees	—	—	Shoot			
167	*Ouret lanata* (L.) Kuntze SKM123	Aerva	*Amaranthaceae*	Whole plant	Wound in the large intestine: whole plant of *A. lanata* (100 g) is made into a paste along with 10 g of freshly collected turmeric (*Curcuma longa*) and 10 g of black cumin (*N. sativa*) or ajwain (*T. ammi*) and administered orally twice a day for 7 days	3	Bullock
	*Curcuma longa* L.	—	—	Rhizome			
	*Nigella sativa* L.	—	—	Seed			
	*Trachyspermum ammi* (L.) Sprague	Caraway	*Apiaceae*	Fruit			
168	*Ouret lanata* (L.) Kuntze ♥	—	—	Whole plant	Foot and mouth disease (FM): 200 g of *A. lanata* plant is made into a paste along with freshly collected turmeric (50 g) and black cumin seed (5 g), given orally as well as applied topically on the affected area once a day till cured	3	Cow, bullock, buffalo
	*Curcuma longa* L.	—	—	Rhizome			
	*Nigella sativa* L.	—	—	Seed			
169	*Alangium salviifolium* (L.f.) Wangerin	—	—	Bark	Retention of the placenta: powder of dried flowers of *B. ceiba* (10 g) is mixed with the powdered stem bark (5 g) of *A. salviifolium* and given to the animal for quick expulsion of the placenta after vaginal delivery of the newborn	2	Cow
	*Bombax ceiba* L.	—	—	Flower			
170	*Myristica fragrans* Houtt.	Nutmeg	*Myristicaceae*	Fruit	Body ache: five fruits of *Myristica* and 250 g of *Amaranthus* stem are made into a paste and applied as a poultice on the affected body part. Before this practice, the affected area is compressed with hot saline water twice a day, and simultaneously an Ayurvedic product is known as “*Chobchini*/*Topchini*” is fed along with sugar in the quantity of 5 g once in a day	2	Bullock, buffalo
	*Amaranthus spinosus* L.	—	—	Stem			
171	*Amaranthus spinosus* L.	—	—	Root	Fever: root of *Amaranthus*—2 pieces (6-7 cm long), mustard seed (50 g), and 21 grains of black pepper are ground together and mixed with holy water. The mixture is then administered once a day for at least 3 days	2	Cow
	*Piper nigrum* L.	—	—	Fruit			
	*Brassica nigra* (L.) W.D.J.Koch SKM26	Black mustard	*Brassicaceae*	Seed			
172	*Lysimachia arvensis* (L.) U.Manns and Anderb. SKM721	Scarlet pimpernel	*Primulaceae*	Whole plant	Throat sore: whole plant of *L. arvensis* (500 g) made into a paste with 100 g of fresh *Curcuma* and applied topically on the affected area twice a day for 3–5 days followed by hot compress	2	Bullock
	*Curcuma longa* L.	—	—	Rhizome			
173	*Ananas comosus* (L.) Merr. SKM323	Pineapple	*Bromeliaceae*	Leaf	Helminthiasis: 250 g of leaf is made into a paste and given with 9–21 grains of black pepper once at night for consecutive 5 days	5	Bullock
	*Piper nigrum* L.	—	—	Fruit			
174	*Solanum virginianum* L.	—	—	Root	High fever: an entire mature plant of *A. paniculata* and one-piece of root (5 cm) of *S. surattense* are made into a paste along with 10 g of black pepper and 10 g of Ajwain. To mature one, this whole mixture of plant paste is given once a day for 3 days	4	Cow
	*Andrographis paniculata* (Burm.f.) Nees	—	—	Shoot			
	*Piper nigrum* L.	—	—	Fruit			
	*Trachyspermum ammi* (L.) Sprague	—	—	Fruit			
175	*Andrographis paniculata* (Burm.f.) Nees	—	—	Whole plant	Snakebite: one mature plant and 21 grains of black pepper are made into a paste and mixed with water to make a water emulsion; orally	9	Bullock
	*Piper nigrum* L.	—	—	Fruit			
176	*Andrographis paniculata* (Burm.f.) Nees	—	—	Shoot	Foot and mouth disease (FM): dried aerial part of one mature plant is made into a paste along with fresh turmeric (5 g) and given orally mixed with old molasses once a day for 7 days	4	Cow, bullock
	*Curcuma longa* L.	—	—	Rhizome			
177	*Aristolochia indica* L. SKM63	Indian Birthwort	*Aristolochiaceae*	Root	Dyspepsia of the calf: 15–20 g of *Aristolochia* root, 5 g of clove, and 5 g of black pepper are crushed and fed along with the cattle feed twice a day for a few days	3	Heifer
	*Syzygium aromaticum* (L.) Merr. and L. M. Perry	Clove	*Myrtaceae*	Flower bud			
	*Piper nigrum* L.	—	—	Fruit			
178	*Aristolochia indica* L.	—	—	Root	Snakebite: three pieces of the root of *Aristolochia* (5–6 cm in size) are made into a paste along with 21 grains of black pepper and given orally twice a day after an interval of 8 h	17	Bullock
	*Piper nigrum* L.	—	—	Fruit			
179	*Asparagus racemosus* Willd.	—	—	Root	Haematuria: fresh root is given with black pepper	7	Gravid cow
	*Piper nigrum* L.			Fruit			
180	*Alstonia scholaris* (L.) R. Br	—	—	Bark	Loose motion: decoction is prepared from 100 g of *Alstonia* bark and 9 grains of black pepper, given orally twice a day for 3 days	4	Bullock
	*Piper nigrum* L.	—	—	Fruit			
181	*Alstonia scholaris* (L.) R. Br	—	—	Bark	Helminthosis: 250 g of *Alstonia* bark, 21 grains of black pepper, and a pinch of rock salt are made into a paste, given orally once at night for 7 days	3	Goat, sheep
	*Piper nigrum* L.	—	—	Fruit			
182	*Azadirachta indica* A. Juss	—	—	Leaf	Dysentery: leaves of *Azadirachta* and rhizome of *Curcuma* (4:1) are made into a paste by maintaining some holly rituals and given orally twice a day for 3 days	9	Cattle
	*Curcuma longa* L.	—	—	Rhizome			
183	*Azanza lampas* (Cav.) Alef. SKM435	Common mallow	*Malvaceae*	Root	Poor lactation: 3-4 pieces of *Azanza* root (5 cm) and 11 grains of black pepper are made into a paste and given once a day for 15 days	3	Milch cow
	*Piper nigrum* L.	—	—	Fruit			
184	*Barleria prionitis* L.	—	—	Shoot	Post-partum debility: the plant parts like 250 g shoot of *B. prionitis* and 21 grains of black pepper are ground together to prepare a paste and administered orally twice a day for 1 month	3	Cow
	*Piper nigrum* L.	—	—	Fruit			
185	*Biophytum sensitivum* (L.) DC. ♥ SKM640	—	—	Root	Stop mastication: 3-4 roots (5 cm) are fed with 21 grains of black pepper once a day for at least 7 days to regularize the digestion process	2	Bullock
	*Piper nigrum* L.	—	—	Fruit			
186	*Boerhavia diffusa* L. SKM165	Red Spiderling	*Nyctaginaceae*	Stem	Post-parturition bleeding: 100 g of the freshly collected stem of *B. diffusa* is made into a paste along with 9–11 black pepper and fed once a day for 3 days	4	Buffalo
	*Piper nigrum* L.	—	—	Fruit			
187	*Vitex negundo* L. SKM208	Chinese chaste tree	*Lamiaceae*	Leaf	Body swelling due to cold (*Jol-sannipat*): leaf of *Vitex*, leaf of *Datura*, and shoot of *B. vitis idaea* (2:2:1) are made into a paste and mixed with the soil of crab hole or sunned rice (*Atop-chal*), heated for some time, and lukewarm paste is applied as poultice throughout the body twice a day	6	Bullock
	*Datura stramonium* L.	—	—	Leaf			
	*Breynia vitis-idaea* (Burm.f.) C.E.C.Fisch. ♠ SKM27	Indian snowberry	*Phyllanthaceae*	Shoot			
188	*Kalanchoe pinnata* (Lam.) Pers. SKM55	Cathedral bells	*Crassulaceae*	Leaf	Dysuria (retention of urine and difficulty in micturition): leaves of *B. pinnatum* (9–12 pieces) and 9–21 grains of black pepper are made into a paste and given orally twice a day for 7–10 days	6	Cow
	*Piper nigrum* L.	—	—	Fruit			
189	*Blumea lacera* (Burm.f.) DC.	—	—	Leaf	Cough: 5–6 leaves of *B. lacera*, 10 g of *T. ammi*, 10 g of *C. longa*, or 25 g of *Z. officinale* are made into a paste and given orally in the morning and evening of a day for 7 days	2	Heifer
	*Trachyspermum ammi* (L.) Sprague	—	—	Fruit			
	*Curcuma longa* L.	—	—	Rhizome			
	*Zingiber officinale* Roscoe	Ginger	*Zingiberaceae*	Rhizome			
190	*Calotropis gigantea* (L.) W.T.Aiton	—	—	Leaf	Fever: 2-3 leaves are made into a paste along with 21 grains of black pepper and given orally twice a day for 3–5 days	3	Cattle
	*Piper nigrum* L.	—	—	Fruit			
191	*Calotropis gigantea* (L.) W.T.Aiton	—	—	Root	Poisoning (*Sapure*): roots of *Calotropis* and black pepper (3:1) are made into a paste and administered along with card or whey in two ways—3/4 parts of the preparation is administered orally twice a day and 1/4 parts of the preparation is applied on the area showing inflammation or swelling due to any kind of poisoning	3	Cow, bullock, buffalo, goat, sheep
	*Piper nigrum* L.	—	—	Fruit			
192	*Calotropis procera* (Aiton) W.T. Aiton SKM79	Sodom apple	*Apocynaceae*	Bark	Body swelling due to cold: bark of *C. procera*, leaves of *J. curcas*, and fresh turmeric (*C. longa*) are made into a paste (2:2:1) and applied as a poultice on the affected area once a day for 5–7 days	3	Bullock
	*Jatropha curcas* L. SKM373	Physic nut	*Euphorbiaceae*	Leaf			
	*Curcuma longa* L.	—	—	Rhizome			
193	*Catunaregam spinosa* (Thunb.) Tirveng. SKM681	Mountain pomegranate	*Rubiaceae*	Root	Urinary tract infection with slight fever: 10 g of the root of *C. spinosa* is made into a paste and given with 9 grains of black pepper once a day for 7 days	2	Goat
	*Piper nigrum* L.	—	—	Fruit			
194	*Causonis trifolia* (L.) Mabb. and J.Wen	—	—	Whole plant	Swelling of the body due to cold (*Jol-sannipat*): whole plant of *C. trifolia* **,** *C. reflexa*, bark of *T. arjuna* (4:1:1) and sunned rice (*Atop-chal*) are made into a paste together, slightly heated, and then applied topically with the help of a brush made of palm-leaf base	9	Cow
	*Cuscuta reflexa* Roxb.	—	—	Whole plant			
	*Terminalia arjuna* (Roxb. ex DC.) Wight and Arn. SKM129	Arjun tree	*Combretaceae*	Bark			
195	*Celastrus paniculatus* Willd. SKM312	Black Oil Plant	*Celastraceae*	Seed	Rheumatoid arthritis: seed oil of *C. paniculatus* and *S. indicum* are mixed in equal proportion and applied externally throughout the affected leg twice a day for at least 15 days	3	Cow
	*Sesamum indicum* L.	—	—	Seed			
196	*Cissus quadrangularis* L.	—	—	Stem	Swelling wart: stem of *C. quadrangularis,* root bark of *F. religiosa* and bark of *T. arjuna* are taken in equal amounts and made into a paste and applied as a poultice on the affected area and tied tightly with cloth and left as it is for a minimum of 15 days	3	Buffalo
	*Ficus religiosa* L.	—	—	Root			
	*Terminalia arjuna* (Roxb. ex DC.) Wight and Arn.	—	—	Bark			
197	*Cissus quadrangularis* L.	—	—	Stem	Helminthosis: 100 g of *Cissus* stem is made into a paste along with 9 grains of black pepper and fed once in the morning for 5–7 days	2	Buffalo
	*Piper nigrum* L.	—	—	Fruit			
198	*Guilandina bonduc* L.	—	—	Root	Retention of placenta: decoction is prepared from 250 g root of *G. bonduc* and 21 grains of black pepper and given orally twice a day	4	Cow
	*Piper nigrum* L.	—	—	Fruit			
199	*Capparis zeylanica* L.	—	—	Leaf	Mastitis: leaf of *C. zeylanica* (50 g) and 21 grains of black pepper are made into a paste together and given orally twice a day till the cure	2	Milch cow
	*Piper nigrum* L.	—	—	Fruit			
200	*Chamaecrista mimosoides* (L.) Greene SKM347	Feather-leaved Cassia	*Fabaceae*	Root	Stomach pain: 5 g of root paste of *Chamaecrista* is given with nine grains of black pepper twice a day for 5–7 days	2	Goat
	*Piper nigrum* L	—	—	Fruit			
201	*Tragia involucrata* L. SKM75	Indian stinging nettle	*Euphorbiaceae*	Root	Fever: the root of *Tragia* (50 g), fruit of *B. anthelmintica* (10 g), and rock salt (5 g) are made into a paste and fed twice a day for 3 days	3	Cow
	*Baccharoides anthelmintica* (L.) Moench	—	—	Fruit			
202	*Chrysopogon zizanioides* (L.) Roberty SKM536	Vetiver grass	*Poaceae*	Root	Headache: mature root of *D. stramonium* is made into a paste along with the roots of *C. zizanioides* and *C. rotundus* (2:1:1), applied as poultice on head twice a day for 2-3 days. In severe cases, little amount of horn dust of spotted deer is added with this preparation and applied in the same way	2	Bullock
	*Cyperus rotundus* L. SKM121	Java grass	*Cyperaceae*	Root			
	*Datura stramonium* L.	—	—	Root			
203	*Cissampelos pareira* L.	—	—	Root	Dog bite: water emulsion is prepared with root (10 g) paste and 21 grains of black pepper and given orally thrice a day for 2 days	9	Goat, sheep
	*Piper nigrum* L.	—	—	Fruit			
204	*Cissampelos pareira* L.	—	—	Leaf	Fever: 4–5 leaves are given along with nine grains of black pepper twice a day for 3 days	2	Calf
	*Piper nigrum* L.	—	—	Fruit			
205	*Cissampelos pareira* L.	—	—	Whole plant	Poisoning (*Aasor*): a paste is prepared from the whole plant of *C. pareira* (250 g), leaf of *A. indica* (5 g), and fresh rhizome of *C. longa* (5 g) and applied topically all over the body	3	Milch cow
	*Azadirachta indica* A. Juss.	—	—	Leaf			
	*Curcuma longa* L.	—	—	Rhizome			
206	*Cocculus hirsutus* (L.) W.Theob.	—	—	Leaf	Urinary incontinence: the leaf of *C. hirsutus* (100 g) is made into a paste along with a little bit of *Zingiber* and rock salt, given once in the morning for 10–15 days	2	Cow
	*Zingiber officinale* Roscoe	—	—	Rhizome			
207	*Cocculus hirsutus* (L.) W.Theob.	—	—	Whole plant	Post-partum debility: an infusion is made from dried plants of *C. hirsutus* and dried bark of *S. febrifuga* (3:1) and given orally once a day for 15 days	2	Cow
	*Soymida febrifuga* (Roxb.) A. Juss.	—	—	Bark			
208	*Holarrhena pubescens* Wall. ex G.Don	—	—	Seed	Bloat (*Dhonrabai*): 10 g seeds of *Wrightia* (*Indrajob*), rhizome of *Curcuma* (250 g), molasses (500 g), and potassium nitrate (5 g) are crushed into a paste together and administered twice a day for a few days; simultaneously lukewarm water is drenched	2	Bullock, buffalo
	*Curcuma longa* L.	—	—	Rhizome			
209	*Swertia chirayita* (Roxb.) H.Karst.	—	—	Whole plant	Fever with ruminal atony: dried whole plant of *S. chirayita* (25–30 g), rhizome of *Curcuma* (50 g), roots of *Tragia* (5–10 g), black pepper (10 g), and potassium nitrate (5 g) are made into a paste together and administered twice a day till recovery	2	Buffalo
	*Curcuma longa* L.	—	—	Rhizome			
	*Tragia involucrata* L. ♥	—	—	Root			
	*Piper nigrum* L.	—	—	Fruit			
210	*Madhuca longifolia* (J.Koenig ex L.) J.F.Macbr. SKM187	Mahua tree	*Sapotaceae*	Bark	Body swelling due to poisoning (*Aasor*): bark of *M. longifolia* and rhizome of *C. longa* (8:1) is made into a paste and applied both topically and orally at the same time of a day twice for 3 days	7	Bullock
	*Curcuma longa* L.	—	—	Rhizome			
211	*Murraya koenigii* (L.) Spreng. ♥ SKM985	Curry leaf tree	*Rutaceae*	Leaf	Drowsiness of small ruminants (*Jhimuni*): leaves of *M. koenigii* and fresh curcuma (4:1) are made into a paste and applied topically on the whole body for consecutive 7 days	6	Sheep, goat
	*Curcuma longa* L.	—	—	Rhizome			
212	*Neolamarckia cadamba* (Roxb.) Bosser	—	—	Leaf	Constipation: leaves of *N. cadamba* (2 kg) and fresh rhizome of *C. longa* (100 g) are made into a paste, and it is fed with molasses (250 g) as a laxative twice a day for 2 days	7	Cow
	*Curcuma longa* L.	—	—	Rhizome			
213	*Neolamarckia cadamba* (Roxb.) Bosser SKM24	Burflower-tree	*Rubiaceae*	Leaf	Constipation: leaf of *N. cadamba* (1.5–2 kg), rhizome of *Curcuma* (250 g), seeds of *Plantago* (250 g), and mustard seed (10 g) are made into a paste and given once a day in case of constipation	4	Buffalo
	*Plantago ovata* Forssk.	Psyllium	*Plantaginaceae*	Seed			
	*Curcuma longa* L.	—	—	Rhizome			
	*Brassica nigra* (L.) K. Koch	—	—	Fruit			
214	*Curcuma aromatica* Salisb. ♥ SKM86	Wild turmeric	*Zingiberaceae*	Rhizome	Food poisoning: rhizome of *C. aromatica* (10–15 g) is made into a paste along with 9–21 grains of black pepper and fed thrice a day for consecutive 2 days	2	Goat
	*Piper nigrum* L.	—	—	Fruit			
215	*Rotheca serrata* (L.) Steane & Mabb. ♠ SKM315	Blue fountain bush	*Lamiaceae*	Root	Fever: one piece of root of *R. serrata* (5 cm of length), a little amount of ginger, and a teaspoon of ajwain are made into a paste and given orally twice a day for 3 days	2	Goat, sheep
	*Zingiber officinale* Roscoe	—	—	Rhizome			
	*Trachyspermum ammi* (L.) Sprague	—	—	Fruit			
216	*Clerodendrum indicum* (L.) Kuntze SKM88	Tubeflower	*Lamiaceae*	Stem	Snakebite: three pieces of the stem (5 cm of length) and 21 grains of black pepper are made into a paste and given orally as soon as possible	3	Cow
	*Piper nigrum* L.	—	—	Fruit			
217	*Volkameria inermis* L.	—	—	Leaf	Rheumatoid arthritis (*Shimola rog*): leaf of *V. inermis* (1 kg), 5 cloves of garlic, and 5 g of asafoetida (*Go-hing*) are made into a paste and applied on the affected area of the leg twice a day for 7–9 days	2	Cow
	*Allium sativum* L.	—	—	Bulb			
	*Ferula assa-foetida* L.	Asafoetida	*Apiaceae*	Gum			
218	*Clerodendrum infortunatum* L.	—	—	Root	General weakness: an infusion is prepared from dried powder of root of *C. infortunatum* and *Soymeda* bark (1:4) and given once a day for at least 15 days	2	Cow, buffalo
	*Soymida febrifuga* (Roxb.) A. Juss.	—	—	Bark			
219	*Colocasia esculenta* (L.) Schott	—	—	Corm	Stiffness of shoulder: bark of *Litsea* and corm of *C. esculenta* are made into a paste (1:1) and applied as a poultice on the affected area twice a day till the cure	2	Bullock, buffalo
	*Litsea glutinosa* (Lour.) C. B. Rob.	—	—	Bark			
220	*Cynodon dactylon* (L.) Pers. SKM122	Bermuda grass	*Poaceae*	Whole plant	Dyspepsia of the calf: a bunch of *C. dactylon* and a little amount of ginger are made into a paste and fed with old molasses once a day for 15 days	2	Calf
	*Zingiber officinale* Roscoe	—	—	Rhizome			
221	*Datura stramonium* L.	—	—	Root	Swelling of body due to poisoning (*Sapure*): root of *Datura* and black pepper (3:1) are made into a paste and administered along with card or whey in two ways; 3/4 parts of the preparation is administered orally twice a day and 1/4 parts of the preparation is applied on the area showing inflammation or swelling due to any kind of poisoning	3	Cow, goat, sheep
	*Piper nigrum* L.	—	—	Fruit			
222	*Pleurolobus gangeticus* (L.) J.St.-Hil. ex H.Ohashi & K.Ohashi ♥	—	—	Whole plant	Bloody dysentery: 100–150 g of the plant along with 21 grains of black pepper is made into a paste and given once a day for 3 days	11	Cow
	*Piper nigrum* L.	—	—	Fruit			
223	*Grona triflora* (L.) H.Ohashi & K.Ohashi SKM197	Three-flower tick-trefoil	*Fabaceae*	Root	Delayed onset of estrous cycle: the root of *G. triflora* is made into a paste and given with a tablespoon of concentrated juice of *S. bengalense* stem*.* This practice is repeated each morning for at least 15 days	2	Heifer
	*Tripidium bengalense* (Retz.) H.Scholz SKM209	Sweetcane	*Poaceae*	Stem			
224	*Dillenia pentagyna* Roxb.	—	—	Leaf	Ulcer in the intestine: leaves (5-6 pieces) are made into a paste along with 11 black pepper (*P. nigrum*) and fed once a day for 3 days	2	Heifer, goat, sheep
	*Piper nigrum* L.	—	—	Fruit			
225	*Wattakaka volubilis* (L.f.) Stapf.	—	—	Stem	Liver trouble: stem of *W. volubilis* (10 cm) made into a paste along with fresh turmeric (*C. longa*) and ajwain (*T. ammi*), given orally once a day for 5 days	3	Cow, bullock
	*Curcuma longa* L.	—	—	Rhizome			
	*Trachyspermum ammi* (L.) Sprague	—	—	Fruit			
226	*Wattakaka volubilis* (L.f.) Stapf.	—	—	Leaf	Mastitis: 4-5 pieces of leaves of *W. volubilis* are made into a paste along with little fresh turmeric (*C. longa*), applied as poultice once a day till cured	3	Milch buffalo
	*Curcuma longa* L.	—	—	Rhizome			
227	*Wattakaka volubilis* (L.f.) Stapf.	—	—	Stem	Haematuria: all the four parts, such as stem of *W. volubilis*, fresh rhizome of *C. longa*, fruits of *T. ammi*, and *C. sativum* seed (4:1:1:1), are made into a paste and given orally	2	Cattle
	*Curcuma longa* L.	—	—	Rhizome			
	*Trachyspermum ammi* (L.) Sprague	—	—	Fruit			
	*Coriandrum sativum* L.	—	—	Fruit			
228	*Drosera burmannii* Vahl ♥ SKM324	Tropical sundew	*Droseraceae*	Whole plant	Infectious diseases: one or two freshly collected *Drosera* plants are fed wrapping with bamboo leaves thrice a week during the onset of certain infectious diseases	3	Cow, buffalo
	*Bambusa bambos* (L.) Voss.	—	—	Leaf			
229	*Echinops echinatus* Roxb.	—	—	Roots	Stomachache: root of *E. echinatus* and whole plant of *S. dulcis* (2:1) is made into a paste, mixed with lukewarm water, and given orally once in the morning for 15 days	5	Buffalo
	*Scoparia dulcis* L.	—	—	Whole plant			
230	*Leucas cephalotes* (Roth) Spreng. SKM15	Spider wort	*Lamiaceae*	Shoot	Rhinorrhoea (*Sonra rog*): freshly collected branches of both the plant *Leucas* and *Eclipta* are taken in equal amount, made shade dry, and powdered, then mixed with mustard oil and vermilion. 2 spoonfuls of this preparation is administered in each nasal opening twice a day	4	Cow
	*Eclipta prostrata* (L.) L. SKM13	False daisy	*Asteraceae*	Shoot			
231	*Glochidion multiloculare* (Rottler ex Willd.) Voigt SKM1123		*Phyllanthaceae*	Bark	Stiffness of shoulder: barks of *G. multiloculare* and *M. longifolia* (2:1) are made into a paste and applied as a poultice on the affected shoulder thrice a day for 3-4 days	5	Bullock, buffalo
	*Madhuca longifolia* (J.Koenig ex L.) J.F.Macbr.	—	—	Bark			
232	*Hemidesmus indicus* (L.) R.Br. SKM827	Indian sarsaparilla	*Apocynaceae*	Root	Sore in the mouth: the root of *H. indicus* is made into a paste along with *C. longa* and smeared on the tongue and other affected areas of the oral cavity	3	Cow
	*Curcuma longa* L.	—	—	Rhizome			
233	*Leea asiatica* (L.) Ridsdale ♥ SKM561	Asiatic Leea	*Vitaceae*	Root	Food poisoning: 20–25 g of the root is made into a paste with 21 grains of black pepper and given orally once a day for 3 days	2	Goat
	*Piper nigrum* L.	—	—	Fruit			
234	*Vicia lens* (L.) Coss. & Germ.	Lentil	*Fabaceae*	Seed	Mastitis: seeds of *V. lens* are made into a paste along with *C. longa* and applied as a poultice on the painful udder thrice a day till cured	2	Milch cow
	*Curcuma longa* L.	—	—	Rhizome			
235	*Leonotis nepetifolia* (L.) R.Br.	—	—	Seed	Infertility: 5 g seeds of *L. nepetifolia* are fed, wrapping with bamboo leaves once a day for regularizing the estrous cycle	3	Buffalo
	*Bambusa bambos* (L.) Voss.	—	—	Leaf			
236	*Lygodium flexuosum* (L.) Sw. ♣ SKM180	Maidenhair creeper	*Schizaeaceae*	Root	Fever: little amount of roots is made into a paste along with 9 grains of black pepper and fed once a day for 3–5 days	2	Goat
	*Piper nigrum* L.	—	—	Fruit			
237	*Xenostegia tridentata* (L.) D.F.Austin & Staples SKM14	African morningvine	*Convolvulaceae*	Whole plant	Stomachache: entire *X. tridentata* plant (15–20 g) and 21 grains of black pepper are made into a paste and given orally once a day in the morning for 3 days	2	Goat
	*Piper nigrum* L.	—	—	Fruit			
238	*Nerium oleander* L.	—	—	Root	Swelling of the body due to poisonous bite (*Sapure*): the root of *Nerium* and black pepper (3:1) are made into a paste and administered, mixing with card or whey in two ways—3/4 parts of the preparation are administered orally twice a day and 1/4 parts of the preparation are applied on the area showing inflammation due to any kind of poisoning	3	Cow
	*Piper nigrum* L.	—	—	Fruit			
239	*Ochna obtusata* DC. SKM816	Ramdhan Champa	*Ochnaceae*	Root	High fever: root of *O. obtusata* (10–15 g) is made into a paste along with a little amount of ginger and fed twice a day till cured	5	Bullock
	*Zingiber officinale* Roscoe	—	—	Rhizome			
240	*Phyllodium pulchellum* (L.) Desv. ♠ SKM460	Showy Desmodium	*Fabaceae*	Bark	Post parturition bleeding: stem bark of *D. pulchellum* and little amount of *C. longa* are made into a paste and given orally twice a day	2	Cow
	*Curcuma longa* L.	—	—	Rhizome			
241	*Zingiber officinale* Roscoe	—	—	Rhizome	Post-partum weakness: Preparation 1: rhizome of *Z. zerumbet* and *Z. officinale* and fruit of *Piper longum* are taken in equal amounts, made into powder separately, and then mixed. Preparation 2: dried rhizomatous root of *P. cubeba* is also made into powder separately. A minimum of 5 g of powder from the preparation 1 and 5–8 g from preparation 2 are taken and mixed well, then administered orally twice a day for a few days	2	Cow
	*Zingiber zerumbet* (L.) Roscoe ex Sm	Pinecone ginger	*Zingiberaceae*	Rhizome			
	*Piper longum* L.	Long pepper	*Piperaceae*	Fruit			
	*Piper cubeba* L. f. ♠	—	—	Root			
242	*Plumbago zeylanica* L. SKM671	Ceylon leadwort	*Plumbaginaceae*	Root	Loose motion: root of *P. zeylanica* (8–10 g), very little amount of fresh rhizome of *C. longa*, and 9 grains of black pepper are made into a paste and given orally twice a day till cure	3	Goat, sheep
	*Piper nigrum* L.	—	—	Fruit			
	*Curcuma longa* L.	—	—	Rhizome			
243	*Premna herbacea* Roxb. SKM261	Stemless premna	*Lamiaceae*	Root	Bloat: 5 g of root and 21 grains of black pepper are made into a paste and given orally once in the evening for 3–5 days	3	Goat
	*Piper nigrum* L	—	—	Fruit			
244	*Polygala crotalarioides* Buch.-Ham. ex DC. SKM227	Indian Milkwort	*Polygalaceae*	Root	Dysentery: root of one plant is made into a paste along with 9 grains of black pepper; 1/3rd of it is given at a time thrice a day in an interval of one hour	5	Goat, sheep
	*Piper nigrum* L.	—	—	Fruit			
245	*Rauvolfia serpentina* (L.) Benth. ex Kurz SKM617	Indian snakeroot	*Apocynaceae*	Root	Poisonous bite: two pieces of roots (3–5 cm) and 21 grains of black pepper are made into a paste with holly water and fed instantly	3	Cow, buffalo
	*Piper nigrum* L.	—	—	Fruit			
246	*Ruellia tuberosa* L. SKM316	Meadow Weed	*Acanthaceae*	Root	Blood dysentery: 4-5 pieces of roots (5–7 cm in length) of *R. tuberosa* are made into a paste along with 21 grains of black pepper and given twice a day for 3 days	3	Cow
	*Piper nigrum* L.	—	—	Fruit			
247	*Ruellia prostrata* Poir. SKM417	Bell Weed	*Acanthaceae*	Whole plant	Swelling of the body due to poisonous effect: freshly collected whole plant (200–250 g) is chopped finely and fed with 21 grains of black pepper	2	Cow
	*Piper nigrum* L.	—	—	Fruit			
248	*Scoparia dulcis* L. ♥	—	—	Whole plant	Retention of placenta: two or three plants of *S. dulcis* are made into a paste along with one tender shoot of *Z. jujuba*, 5 g of ajwain (*T. ammi*), and 5 gm of turmeric (*C. longa*) and given orally once a day in the morning for 3 consecutive days	2	Cow, buffalo
	*Ziziphus jujuba* Mill. ♣ SKM444	Chinese jujube	*Rhamnaceae*	Shoot			
	*Trachyspermum ammi* (L.) Sprague	—	—	Fruit			
	*Curcuma longa* L.	—	—	Rhizome			
249	*Scoparia dulcis* L. ♥	—	—	Whole plant	Fever: a bunch of dried plants of *S. dulcis* and stem of *S. glaucophyllum* are taken in a 3:1 ratio and made into a paste with black pepper (3–21 grains). The whole preparation is fed at a time once a day for 3 consecutive days	3	Cow
	*Solanum glaucophyllum* Desf.	—	—	Stem			
	*Piper nigrum* L.	—	—	Fruit			
250	*Shorea robusta* Gaertn. SKM81	Sal tree	*Dipterocarpaceae*	Bark	Diarrhea: 250 g of the bark of *S. robusta* is made into a paste with 21 grains of black pepper and given orally once a day till cure	2	Cow
	*Piper nigrum* L.	—	—	Fruit			
251	*Sida cordifolia* L. ♥ SKM190	Heart-leaf Sida	*Malvaceae*	Stem	Loose motion: stem of *S. cordifolia* is made into a paste along with cumin seed (*C. cyminum*) soaked water and administered orally once a day for consecutive 3 mornings	7	Calf, goat, sheep
	*Cuminum cyminum* L.	Cumin	*Apiaceae*	Seed			
252	*Sida rhombifolia* L. SKM294	Arrowleaf sida	*Malvaceae*	Root	Fever: a paste is made with 10–15 g of *S. rhombifolia* root and 9 grains of black pepper and fed along with mustard cake	8	Calf, goat, sheep
	*Piper nigrum* L.	—	—	Fruit			
253	*Smilax ovalifolia* Roxb. ex D.Don	—	—	Root	Bloody dysentery: root paste (5–10 g) is administered with 9–21 pieces of black pepper (according to age) once a day for 3 days	5	Cow
	*Piper nigrum* L.	—	—	Fruit			
254	*Spondias pinnata* (L. f.) Kurz SKM307	Hog plum	*Anacardiaceae*	Bark	Fever: 100 g of bark of *S. pinnata* and 10–15 g of stem of *S. glaucophyllum* are ground along with black pepper (3–21) and given orally twice a day for 3 days	2	Bullock
	*Solanum glaucophyllum* Desf.	—	—	Stem			
	*Piper nigrum* L.	—	—	Fruit			
255	*Swertia chirayita* (Roxb.) H.Karst.	—	—	Whole plant	Fever: dried plant of *S. chirayita* (5–8 g), root of *T. involucrata* (5-6 pieces), fruit of black pepper (for goat, 5–15 pieces; for cow, 9–45 pieces; for buffalo, 15–61 pieces). Fixed amounts of all these three plant parts are made into a paste and given twice (morning and evening) a day till cure	4	Buffalo, goat
	*Tragia involucrata* L.	—	—	Root			
	*Piper nigrum* L.	—	—	Fruit			
256	*Tacca leontopetaloides* (L.) Kuntze ♠ SKM503	Indian Arrow Root	*Dioscoreaceae*	Tuber	Diarrhea: tuber (10–15) is made into a paste with 9–11 grains of black pepper and given orally once a day for 3 days	3	Buffalo
	*Piper nigrum* L.	—	—	Fruit			
257	*Terminalia chebula* Retz.	Myrobalan	*Combretaceae*	Fruit	Liver trouble with sore in intestine: 5-6 mature fruits of *T. chebula*, 5 g fresh rhizome of *C. longa*, and 9–21 grains of black pepper are made into a paste with holly water and fed once a day for 7 days	3	Bullock
	*Curcuma longa* L.	—	—	Rhizome			
	*Piper nigrum* L.	—	—	Fruit			
258	*Tinospora sinensis* (Lour.) Merr.	—	—	Stem	Fever with breathing trouble: stem of *T. sinensis* and rhizome of *Z. officinale* are made into a paste in a 5:1 ratio and given orally once a day for 9–11 days	3	Bullock
	*Zingiber officinale* Roscoe	—	—	Rhizome			
259	*Tragia involucrata* L.	—	—	Root	Fever: roots of *Tragia* and fruits of *Capsicum* or black pepper are crushed together to make a paste and given with cattle feed once or twice a day (as per cow's age) for 2-3 days. Dose: for adult cow, root of *Tragia* (30–35 cm), *Capsicum* (5-6 pieces), or *Piper* (10–12 pieces)	4	Cow, bullock, buffalo
	*Piper nigrum* L.	—	—	Fruit			
	*Capsicum annuum* L. SKM127	Chili	*Solanaceae*	Fruit			
260	*Ventilago denticulata* Willd. SKM106	Red creeper	*Rhamnaceae*	Shoot	Diarrhea: 3-4 tender shoots (where leaf number will be 5) are made into a paste along with black pepper and given orally once in the morning for 3 consecutive days	3	Bullock
	*Piper nigrum* L.	—	—	Fruit			
261	*Zingiber officinale* Roscoe	—	—	Rhizome	Bloat (*Dhonrabai*): rhizome of *Zingiber* (25 g), fruits of *Trachyspermum* (25 g), salt (25 g), and molasses (25 g) are ground into paste, and then administered twice a day for 2-3 days	3	Cow
	*Trachyspermum ammi* (L.) Sprague	—	—	Fruit			
262	*Zingiber officinale* Roscoe	—	—	Rhizome	Gut erosion, pulmonary congestion, foot rot, and any kind of poisoning of the cattle: rhizomes of *Z. officinale* and *Z. zerumbet* are made into a paste along with the fruits of *P. longum* in a 2:1:1 ratio and administered orally twice a day for 5–7 days	2	Cow, buffalo
	*Zingiber zerumbet* (L.) Roscoe ex Sm. ♠	—	—	Rhizome			
	*Piper nigrum* L.	—	—	Fruit			
263	*Ziziphus jujuba* Mill.	—	—	Shoot	Diarrhea: three pieces of tender shoots of *Z. jujuba* are made into a paste along with 9–21 grains of black pepper (*P. nigrum*) and mixed with water; oral: twice for 3 days	9	Cow
	*Piper nigrum* L.	—	—	Fruit			
**C**	**Magico-religious belief (*n* = 12)**	—	—	—	—	—	—
264	*Abutilon indicum* (L.) Sweet ♣ SKM01	Monkey bush	*Malvaceae*	Root	Watering of eyes: a small piece of root is touched on the affected eye thrice at a time, then that root piece is touched on the ground and is dipped in a certain site of a nearby pond situated corresponding to the side (left/right) of the affected eye. It is performed on three consecutive Sundays of a month. A regular compress of steam vapor is given simultaneously	4	Cow
265	*Abutilon indicum* (L.) Sweet	—	—	Stem	Unusual aggressiveness (during milking): a dried stem is tied and hung around the neck with a black thread	4	Milch cow
266	*Adiantum philippense* subsp. *philippense* ♠	—	—	Rhizome	Sudden decrease in milk production: a little bunch of dried rhizomatous root is tied around the neck with a red thread	2	Milch cow
267	*Dioscorea bulbifera* L.	—	—	Bulbil	Weakness: a bulbil is hung on the neck with a piece of black thread	2	Cow, buffalo
268	*Euphorbia antiquorum* L.	—	—	Latex	Infectious disease: a round mark is drawn with freshly collected latex on the right side of the head	2	Large bovine animal
269	*Martynia annua* L.	—	—	Fruit	Weakness: three dried fruits are hung with the black thread from the neck of cattle to combat evil forces	2	Gravid cow
270	*Musa paradisiaca* L.	—	—	Leaf	Dysentery: apical part of a tender leaf is wrapped with a piece of cloth smeared with fresh curcuma paste and tied on any of the back legs with black colored ribbon	2	Gravid cow
271	*Neptunia prostrata* (Lam.) Baill. SKM551	Water Mimosa	*Fabaceae*	Stem	Poisonous bite (snake): three dried stems of equal size are tied firmly near the site of the poisonous bite	2	Cow, bullock
272	*Streblus asper* Lour. SKM203	Siamese rough bush	*Moraceae*	Leaf	Opacity of cornea: 21 leaves are touched on the affected eye and then dipped in a certain site of a nearby pond	2	Cow
273	*Semecarpus anacardium* L.f.	—	—	Latex	Lameness in the hind leg (*Shir-taan*): latex come out of a freshly collected fruit is touched at the base of the hoof of the front legs, horns, and tail	5	Bullock
274	*Swietenia macrophylla* King. SKM624	Broad-leafed Mahogany	*Meliaceae*	Fruit	Miscarriage: for the safety of the fetus and to prevent miscarriage, dried fruit of *Swietenia* is tied around the neck of the pregnant cow with a red ribbon	2	Gravid buffalo
275	*Tragia involucrata* L.	—	—	Root	Maggot infested wounds (between hooves and in genital openings): a piece of root is tied with red colored thread and hung around the neck of affected cattle. This practice is done only on Sunday and Tuesday of the week	7	Cow

### 3.3 Quantitative Analysis of the Recorded Ethnoveterinary Medicinal Data

A total of 1,234 citations were made by the 132 participants. All the recorded 79 health issues are grouped into 20 disease categories based on the *emic* perception of the participants as consulted during focus group discussion.

Fic value was determined for all the 20 diseases categories, ranging from 0.4 to 0.83 (Table 2). Among the recorded disease categories, six categories such as skeletal disorders, helminthiasis, urinary disorders, poisonous effect, retention of milk, and enteric diseases showed very high Fic value (≥0.8); that is, the value is significantly closer to 1, which means there is a greater consensus among the participants. Eleven disease categories were found to have moderate Fic value (≥0.6 to ˂0.8). Moreover, three disease categories such as rheumatic disorder (0.57), ophthalmic disorder (0.53), and general health weakness (0.4), have scored low Fic value (<0.6).

**TABLE 2:** Factor for informant’s consensus (Fic) value of the 20 disease categories and the most reliable ethnoveterinary medicinal plants (EVMPs) recorded in each disease category.

Sl. No.	Disease category	Number of taxa (nt)	Number of use report (nur)	Fic value	EVMPs with fidelity level (FL%) and use-mention (UM) factor
1	Skeletal disorder	08	41	0.83	*Cissus quadrangularis* L. (90.48; UM = 19)
2	Helminthiasis	07	34	0.82	*Pueraria tuberosa* (Willd.) DC. (100; UM = 17)
3	Enteric diseases	29	151	0.81	*Abrus precatorius* L. (84.21; UM = 16), *Vachellia nilotica* subsp. *indica* (Benth.) Kyal. & Boatwr. (86.67; UM = 13)
4	Poisonous effect	23	119	0.81	*Aristolochia indica* L. (94.44; UM = 17), *Causonis trifolia* (L.) Mabb. & J.Wen (91.67; UM = 11)
5	Urinary disorders	11	54	0.81	*Wattakaka volubilis* (L.f.) Stapf. (81.82; UM = 09)
6	Disorders in milk production	14	65	0.8	*Amaranthus spinosus* L. (64.71; UM = 11)
7	Fever and related problems	21	94	0.78	*Achyranthes aspera* L. (95.65; UM = 22), *Tragia involucrata* L. (81.82; UM = 09)
8	Gastrointestinal disorders	46	199	0.78	*Curcuma longa* L. (91.67; UM = 22)
9	Ectoparasite	09	36	0.77	*Annona squamosa* L. (100; UM = 11)
10	Infectious disease	13	51	0.76	*Achyranthes aspera* L. (39.13; UM = 09)
11	Cold, cough, and related problems	17	62	0.74	*Luffa acutangula* (L.) Roxb. (100; UM = 11)
12	Cuts and wounds	18	67	0.74	*Allium sativum* L. (100; UM = 09)
13	Muscular disorders	04	12	0.73	*Agave americana* L. (55.56; UM = 05)
14	Magico-religious cure	11	37	0.72	*Tragia involucrata* L. (63.64; UM = 07)
15	Mastitis	13	36	0.66	*Nigella sativa* L. (100; UM = 07)
16	Reproductive health disorders	18	49	0.65	*Gloriosa superba* L. (100; UM = 07)
17	Dermatological disorder	22	53	0.6	*Agave americana* L. (55.56; UM = 0.5)
18	Rheumatic disorder	10	22	0.57	*Calotropis gigantea* (L.) W.T.Aiton (63.63; UM = 07)
19	Ophthalmic disorder	12	26	0.56	*Coccinia grandis* (L.) Voigt (83.33; UM = 05)
20	General weakness	16	26	0.4	*Pueraria tuberosa* (Wild.) DC. (41.18; UM = 17)

The fidelity level or FL values have been determined for all the recorded species used in those 20 diseases categories. Among the recorded plant species, only 23 EVM plants have been identified here as the most important species in the respective disease condition according to their FL value (Table 2).

Further, 68 EVMPs have been considered, which were cited by at least 5% of the participants (FC ≤ 7) for further ranking after comparing the numerical values of frequency of citation (FC), use reports (UR), and the number of uses (NU) and based on the score of CV index (Table 3). After careful comparison of all the values estimated for the 68 plant species, nine species have been considered as culturally most valuable (CV ≥ 0.0025 and frequency of citation ≥20) in the northern laterite region of West Bengal. Those nine plants are *Curcuma longa* L., *Achyranthes aspera* L., *Abrus precatorius* L., *Amaranthus spinosus* L., *Azadirachta indica* A.Juss., *Cissus quadrangularis* L., *Pueraria tuberosa* (Willd.) DC., *Andrographis paniculata* (Burm.f.) Nees, and *Wattakaka volubilis* (L.f.) Stapf.

**TABLE 3:** Ranking of the useful EVMPs (*n* = 68) on the basis of the cultural value (CV) index.

EVM plants	Basic values			Score of CV index	Ranking
	FC (frequency of citation)	UR (number of use reports)	NU (number of uses)		
*Curcuma longa* L.	44	24	13	0.013786	**1**
*Achyranthes aspera* L.	53	23	7	0.011344	**2**
*Abrus precatorius* L.	40	19	4	0.006969	**3**
*Amaranthus spinosus* L.	25	17	5	0.006095	**4**
*Azadirachta indica* A.Juss.	20	16	5	0.004598	**5**
*Cissus quadrangularis* L.	24	21	3	0.004340	**6**
*Pueraria tuberosa* (Willd.) DC.	27	17	4	0.003966	**7**
*Andrographis paniculata* (Burm.f.) Nees	22	13	4	0.003253	**8**
*Wattakaka volubilis* (L.f.) Stapf.	20	11	5	0.002523	**9**
*Vachellia nilotica* subsp. *indica* (Benth.) Kyal. & Boatwr.	19	15	3	0.002445	**10**
*Aristolochia indica* L.	20	18	2	0.002067	**11**
*Scoparia dulcis* L.	16	11	5	0.002025	**12**
*Tragia involucrata* L.	20	11	5	0.001693	**13**
*Pleurolobus gangeticus* (L.) J.St.-Hil. ex H.Ohashi & K.Ohashi	20	13	2	0.001479	**14**
*Causonis trifolia* (L.) Mabb. & J.Wen	20	12	2	0.001374	**15**
*Calotropis gigantea* (L.) W.T.Aiton	13	11	3	0.001232	**16**
*Datura stramonium* L.	15	9	3	0.001162	**17**
*Agave americana* L.	13	9	3	0.001009	**18**
*Musa paradisiaca* L.	14	8	3	0.000960	**19**
*Cissampelos pareira* L.	21	12	4	0.000891	**20**
*Soymida febrifuga* (Roxb.) A. Juss.	14	7	4	0.000858	**21**
*Artocarpus heterophyllus* Lam.	13	11	2	0.000813	**22**
*Litsea glutinosa* (Lour.) C. B. Rob.	12	6	5	0.00081	**23**
*Alstonia scholaris* (L.) R. Br.	10	9	3	0.000775	**24**
*Madhuca longifolia* (J.Koenig ex L.) J.F.Macbr.	12	11	2	0.000755	**25**
*Asparagus racemosus* Willd.	13	9	2	0.000666	**26**
*Alangium salviifolium* (L.f.) Wangerin	8	7	4	0.000636	**27**
*Cuscuta reflexa* Roxb.	12	9	2	0.000618	**28**
*Terminalia arjuna* (Roxb. ex DC.) Wight & Arn.	12	9	2	0.000618	**29**
*Smilax ovalifolia* Roxb. ex D.Don	10	7	3	0.000604	**30**
*Ziziphus jujuba* Mill.	11	9	2	0.000564	**31**
*Echinops echinatus* Roxb.	9	7	3	0.000540	**32**
*Zingiber officinale* Roscoe	7	5	6	0.000503	**33**
*Ficus benghalensis* L.	9	9	2	0.000462	**34**
*Swertia chirayita* (Roxb.) H.Karst.	12	8	4	0.000457	**35**
*Helicteres isora* L.	10	7	2	0.000402	**36**
*Semecarpus anacardium* L.f.	8	8	2	0.000372	**37**
*Abutilon indicum* (L.) Sweet	8	7	2	0.000360	**38**
*Clerodendrum infortunatum* L.	9	7	2	0.000360	**39**
*Gloriosa superba* L.	9	7	2	0.000360	**40**
*Annona squamosa* L.	11	11	1	0.000344	**41**
*Carica papaya* L.	11	11	1	0.000344	**42**
*Luffa acutangula* (L.) Roxb.	11	11	1	0.000344	**43**
*Casearia tomentosa* Roxb.	8	7	3	0.000318	**44**
*Sesamum indicum* L.	9	6	2	0.000306	**45**
*Neolamarckia cadamba* (Roxb.) Bosser	11	9	2	0.000282	**46**
*Barleria prionitis* L.	8	6	2	0.000274	**47**
*Coccinia grandis* (L.) Voigt	8	6	2	0.000274	**48**
*Tinospora sinensis* (Lour.) Merr.	8	6	2	0.000274	**49**
*Guilandina bonduc* L.	7	6	2	0.000238	**50**
*Allium sativum* L.	9	9	1	0.000231	**51**
*Bambusa bambos* (L.) Voss.	9	9	1	0.000231	**52**
*Ziziphus nummularia* (Burm.f.) Wight & Arn.	9	9	1	0.000231	**53**
*Coriandrum sativum* L.	7	5	2	0.000201	**54**
*Kalanchoe pinnata* (Lam.) Pers.	8	8	2	0.000183	**55**
*Chenopodium album* L.	8	8	1	0.000183	**56**
*Sida rhombifolia* L.	8	8	1	0.000183	**57**
*Senegalia catechu* (L.f.) P.J.H.Hurter & Mabb.	9	7	2	0.000140	**58**
*Cajanus scarabaeoides* (L.) Thouars	7	7	1	0.000140	**59**
*Enydra fluctuans* DC.	7	7	1	0.000140	**60**
*Strobilanthes hirta* (Vahl) Blume	7	7	1	0.000140	**61**
*Linum usitatissimum* L.	7	7	1	0.000140	**62**
*Luffa aegyptiaca* Mill.	7	7	1	0.000140	**63**
*Mimosa pudica* L.	7	7	1	0.000140	**64**
*Nigella sativa* L.	7	7	1	0.000140	**65**
*Sida cordifolia* L.	7	7	1	0.000140	**66**
*Solanum virginianum* L.	9	5	2	0.000129	**67**
*Solanum glaucophyllum* Desf.	8	5	3	0.000072	**68**

The Spearman rank-order Correlation analysis has been performed taking the cultural value (CV) index and three basic values of frequency of citation (FC), use report (UR), and use diversity (NU) as variables to check the dependency of one upon another (Table 4). The result expressed very significant correlations among all the variables at *p* < 0.05. The analysis highlighted that the CV index is highly dependent on the value of FC and UR (correlation coefficient > 0.9, which is near to 1). Therefore, the versatile uses of a plant species and its familiarity among the participants of a particular area significantly influence the CV index, which reflects the overall importance of the plant species in the culture.

**TABLE 4:** Spearman rank-order correlations among the variables.

	FC	UR	NU	CV
FC	—	0.98	0.631	0.967
UR	—	—	0.731	0.982
NU	—	—	—	0.75
CV	—	—	—	—

Analysis of the descriptive statistics has revealed that the values of mean (M) and standard deviation are very low in the CV index (M = 0.00044, SD = 0.00147), which indicates the accuracy of estimating the overall cultural importance of a species by this index.

Ten species out of 232 recorded plant species have frequently been used as minor ingredients in 110 different polyherbal preparations along with their respective principal ingredients. Those 10 species are *Piper nigrum* L., *Curcuma longa* L., *Zingiber officinale* Roscoe, *Piper longum* L., *Nigella sativa* L., *Trachyspermum ammi* (L.) Sprague, *Cuminum cyminum* L., *Piper cubeba* L.f., *Ferula assa-foetida* L., and *Allium sativum* L. Ranking of preferred species has been made on the basis of scores given to the species considering the use-preference of the ten key participants. The list of most preferred herbal ingredients is presented in Table 5. *Piper nigrum* L. was ranked in the first position with the highest score of 86 out of 100, which revealed that the fruit of this plant is the most preferred ingredient used in polyherbal preparations by the participants of the studied area. The popularity of this plant is assumed easily by observing the value of frequency of citation (FC = 62) and use value (UV = 0.462), which are the highest among the values of these two parameters for all the recorded medicinal species in the studied area.

**TABLE 5:** Preference ranking exercise with 10 plant species used as additional ingredients in the polyherbal ethnoveterinary medicines. Here 10 key participants labeled from A to J (average age = 65 ± 10 years).

Plants used as additional ingredient	No. of citation/use mention	Use value (UV)	Preference ranking score given by the 10 key participants											Rank order
			A	B	C	D	E	F	G	H	I	J	Total score	
*Piper nigrum* L.	61	0.462	9	10	7	9	9	8	8	9	7	10	86	1
*Curcuma longa* L.	42	0.318	7	8	5	6	7	6	3	7	5	7	61	2
*Zingiber officinale* Roscoe	16	0.121	5	3	3	2	3	4	2	5	3	5	35	3
*Piper longum* L.	4	0.03	2	0	1	2	2	2	3	2	3	3	20	4
*Nigella sativa* L.	21	0.159	2	2	1	0	0	4	2	3	2	2	18	5
*Trachyspermum ammi* (L.) Sprague	21	0.159	0	3	0	0	0	4	3	2	2	2	16	6
*Cuminum cyminum* L.	7	0.053	0	2	0	0	0	3	2	3	2	2	14	7
*Piper cubeba* L. f.	2	0.015	0	0	0	0	0	3	2	4	2	2	13	8
*Ferula assa-foetida* L.	2	0.015	0	2	0	0	0	2	1	2	1	1	9	9
*Allium sativum* L.	2	0.015	0	0	2	0	0	3	0	1	2	0	8	10

A total of 68 participants from the Birbhum district and 64 from the Burdwan district participated in the present study. The knowledge similarity between the participants of these two adjacent districts of the studied area has been analyzed employing the Jaccard index (JI). Among the recorded 232 EVMPs, uses of 139 plants were known to the participants from the Burdwan district, and uses of 202 plants were recorded from the participants of the Birbhum district. It has also been observed that uses of 108 EVMPs are common for both districts. The result of JI revealed that, across the 232 plant species, knowledge similarity reaches up to 46.35%, which is quite high as expected because both the districts are adjoining to each other and share similar environmental conditions, ethnic compositions, cultural values, and forest types. Another possible cause for such a high percentage of knowledge similarity is the cross-cultural exchange of EVM knowledge among the inhabitants of these two neighboring districts.

## 4. Discussion

### 4.1 Is Ethnoveterinary Medicinal Knowledge Depends on the Informant’s Age, Gender, Education, and Knowledge Gathering Pattern?

The involvement of participants indicates gender biases in the present study. However, most of the earlier workers have also experienced a similar type of a male dominating informant composition in their ethnomedicinal explorations carried out in different parts of the world (Hassan et al., 2017; Aziz et al., 2018). In the studied area, the role of women was found restricted mainly in assisting livestock rearing and dairying. Male participants of the study area have much more knowledge about traditional livestock healthcare than the female participants.

The expertise in folk therapy does not depend on the formal education and literacy of the participants but on their keen observation, deep interest, and analytical attitude toward traditional knowledge. Dissemination and gathering of the knowledge occur verbally, and the proficiency of an informant depends on their perception of knowledge and accuracy of practicing the same. In many cases, it has been noticed that the persons with no formal education are much more knowledgeable about folk therapy than the literate ones (Hayta et al., 2014; Umair et al., 2017).

Ninety-one participants interviewed were above 50 years with a minimum of 25 years of experience in livestock healthcare management. They have contributed the maximum number of information (281 formulations, 87%) about the uses of ethnoveterinary medicinal plants. The experience is increased by acquiring knowledge from different sources with an increase in age (Ayantunde et al., 2008). A similar type of scenario has been noticed in many of the ethnobotanical studies carried out earlier by other workers (Piluzza et al., 2015; Bullitta et al., 2018). It is a serious concern that the younger generation of the indigenous community of the northern laterite region of West Bengal is less interested in their age-old therapeutic practices and the traditional knowledge. The gradual inclination toward modern lifestyle, growing faith in allopathic medicine, modern education, and several other cultural changes within the traditional community profoundly influence the younger generation to throw away their forefathers’ culture, which put the age-old knowledge system on the verge of extinction (Cox, 2000).

In the present investigation, the rate of vertical transmission of knowledge is quite higher than the horizontal and oblique ones. In traditional societies, there is a common belief that the religious trust in medicine and its secrecy maintains the purity and supremacy of folk remedies (Giday et al., 2009). Such a kind of social belief restricts the knowledge transmission mainly within the family members. It is identified as one of the main causes of the highest percentage of vertical transmission of ethnomedicinal knowledge in the study area.

### 4.2 Ethnoveterinary Medicinal Plant Resource and Knowledge Richness

Recorded 232 EVMPs extend the previously documented EVMPs list to 315 in the state of West Bengal, which indicates the richness of ethnoveterinary medicinal plants and its associated traditional veterinary knowledge among the local people in the study area (Jain and Dey, 1966; Pal, 1980; Pal, 1981; Mukherjee and Namahata, 1988; Pal, 1992; Ghosh, 1999; Ghosh, 2002; Ghosh, 2003; Bandyopadhyay and Mukherjee, 2005; Mitra and Mukherjee, 2007; Ghosh, 2008; Das and Tripathi, 2009; Rahaman et al., 2009; Dey and De, 2010; Pandit, 2010; Saha et al., 2010; Ghosh, 2011; Saha et al., 2014). Besides West Bengal, a total number of EVMPs has been estimated from the other parts of India. From the state of Gujarat, 265 plant species of veterinary importance were documented a few years back (Katewa et al., 2010). A total of 294 EVMPs have been reported from the states of North-East India (Sharma, 2012). In a recent review, about 449 EVMPs have been reported from the Indo-Gangetic region (Sikarwar, 2017). It is interesting to mention that, among the total number of veterinary plant taxa recorded from the Indo-Gangetic part of India, 129 EVMPs were common in the present study.

In many ethnobotanical studies, the family *Fabaceae* has been identified as the richest in medicinal flora among the plant families (Leonti et al., 2003; Molares and Ladio, 2009). A variety of biologically active phyto-constituents from different biochemical groups, such as tannins, flavonoids, alkaloids, and terpenes, have so far been reported from different medicinal species of this family, which largely influence their effective use in global folk medicine (Leonti et al., 2002). In the present study, the medicinal use of the highest number of leguminous species also conforms to the diversified therapeutic potentialities of this plant family *Fabaceae*.

Among the recorded plant taxa, most species are of herbaceous type (43%). The use of the herbs in the highest number in remedy preparation is their abundant growth and easy availability in the locality. It is a fact that humans would prefer to search for food and medicinal plants, which are most abundant, easy to access, and available all around the year (Albuquerque et al., 2005). For these reasons, herbaceous plants have occupied a considerable percentage among the medicinal plants used in almost all the traditional systems of medicine including ethnomedicine in the world (Disler et al., 2014; Eshetu et al., 2015; Parthiban et al., 2016).

Folk medicinal practices are based mainly on wild-growing plants, and this tradition of using local wild plants is still enduring in most ethnic cultures. The present investigation has also witnessed that most of the EVM species (86.15% of the total recorded species) were collected from wild sources, confirming the local people’s dependence on mostly the plants growing in the wild.

Herein, the recorded main plant parts used for remedy preparation are the underground parts. Most of the ethnobotanical explorations carried out in different parts of the world exhibited that roots and underground parts of the plants are used as medicine in the highest or considerable percentage (Mall, 2009; Agrawal, 2013). Roots and other underground parts have been identified as the major sites where many of the bioactive compounds are synthesized and accumulated, further highlighting the scientific basis of these folk herbal practices of using underground parts of the plants (Flores and Flores, 1997; Bais et al., 2001). Most of the time, it has been informed that the collection of underground parts destroys the plant. Such an unsustainable collection practice resulted in the reduction or depletion of the local phytodiversity (Kimondo et al., 2015). Therefore, excessive collection of underground plant parts can cause a threatful impact on local biodiversity.

The present study witnessed the use of a significant percentage (36% of total remedies recorded) of polyherbal recipes. It is justified because active principles present in different herbal ingredients of a polyherbal recipe exert better therapeutic thrust through their synergistic effect (Amodu et al., 2013; Malik et al., 2017). Therefore, the uses of such polyherbal remedies involving diversified medicinal plants for cattle health indicate the depth and width of the knowledge regarding traditional healthcare for the veterinary animals in the area.

In case of external or topical application, the paste is mainly used as poultice because it is comparatively convenient to apply to the exterior of the affected body part of the veterinary animals. Besides, it is effortless to administer the paste with the animals’ feed orally. It has been noticed that the administration of paste for the treatment of various health disorders of both humans and livestock is a common practice in traditional medicines throughout the world (Giday et al., 2010; Vijayakumar et al., 2016). The probable cause for applying paste in a higher percentage is that paste is prepared easily within a short time by a simple method using convenient and small tools.

Like the present study, most of the cases of oral administrations of folk preparation have been encountered in several earlier studies (Jorim et al., 2012; Aziz et al., 2018). Side by side topical application of folk remedies remains an important mode of drug administration to treat diseases, such as skin disorders, wounds, rheumatic pain, and body pain (Sargin et al., 2013; Tariq et al., 2016). Specifically, topical use of poultice increases blood circulation in the affected portions of the body. It also protects the infected wounds or sores from microbial infection again by providing a protective cover to the affected parts in the form of a medicated layer of drug substances. Moreover, many healing substances of the medicinal herbs present in a poultice (such as antiseptic essential oils, phenolics, and tannins) infiltrate through animal tissues, helping it fight against infection and reduce inflammation. Finally, healing of the wound is promoted.

### 4.3 Magico-Religious Healing

Superstition and magico-religious belief are very much integrated with the life, culture, and health of the ethnic people worldwide (Ahirwar, 2015; Pangging et al., 2018). Like other tribal or ethnic communities, indigenous people of the present study area have a strong faith in magico-religious practices performed to cure and diagnose certain diseases of their domesticated animals. They believe that certain diseases in veterinary animals are caused by the bad influences of evil spirits or some supernatural powers. They treat those sick animals by holy chanting, performing special rituals, and offering prayers and sacrifices to appease the suspected evil power by which, according to their belief, certain disease conditions are developed.

### 4.4 New Uses

After thoroughly checking the relevant books and research articles on ethnoveterinary medicine published from India, 68 EVMPs are found new in several aspects of the existing inventory of Indian ethnoveterinary medicine (Table 1 and Supplementary Table S4). The ethnoveterinary uses of 24 plant species documented in this investigation are exclusively new for India as they have not been reported in the standard literature consulted (Pal and Jain, 1998; Jain, 1999; Ghosh, 2003; Rahaman et al., 2009; Katewa et al., 2010; Jain, 2012; Kumar et al., 2012; Saha et al., 2014; Jain and Jain, 2016; Sikarwar, 2017). The 24 EVM plants identified as new for their uses are *Abutilon hirtum* (Lam.) Sweet, *Aerva javanica* (Burm.f.) Juss. ex Schult., *Albizia procera* (Roxb.) Benth., *Coleus strobilifer* (Roxb.) A.J.Paton, *Cajanus goensis* Dalzell, *Breynia vitis-idaea* (Burm.f.) C.E.C.Fisch., *Caladium bicolor* (Aiton) Vent., *Centipeda minima* (L.) A.Braun & Asch., *Cotula anthemoides* Lour., *Croton persimilis* Müll.Arg., *Eulophia explanata* Lindl., *Hydrolea zeylanica* (L.) Vahl, *Ipomoea cairica* (L.) Sweet, *Ipomoea obscura* (L.) Ker Gawl., *Jatropha nana* Dalzell & A.Gibson, *Phoenix acaulis* Roxb., *Phyllodium pulchellum* (L.) Desv., *Piper cubeba* L.f., *Rotheca serrata* (L.) Steane & Mabb., *Seseli diffusum* (Roxb. ex Sm.) Santapau & Wagh, *Tacca leontopetaloides* (L.) Kuntze, *Uraria lagopoides* (L.) DC., *Zingiber zerumbet* (L.) Roscoe ex Sm., and *Ludwigia adscendens* (L.) H.Hara.

It has been noticed that 31 recorded taxa reported earlier as EVMPs are found new in respect of the diseases cured by them. The plant *Alangium salviifolium* (L.f.) Wangerin was reported earlier for the treatment of cattle suffering from cough, liver trouble, and poisonous bite (Jain, 1999; Galav et al., 2013), but the same plant was recorded here for the use in curing general weakness.

Five investigated taxa of the present work differ in respect of remedy preparation modes with the earlier reports made by different workers, although those five plants are used for curing similar types of diseases such as fever, wound, mastitis, arthritis, and dysentery. For example, leaf of *Nicotiana rustica* L. is solely used as a germicide to heal cattle wounds (Jain, 1999). However, the leaf of it is administered here topically as paste along with mustard oil and “Sankhachurna” (a rich source of calcium carbonate) for the same purpose. Again, four EVMPs have been found new regarding their parts used. For example, leaves of *Abutilon indicum* (L.) Sweet have been reported earlier for eye problems (Jain, 1999), but here, for the same purpose, using the root of the same plant is exclusively a new report. Apart from the new uses of angiospermic taxa, ethnoveterinary medicinal uses of Pteridophytes such as *Adiantum philippense* subsp. *philippense* is reported first time here as EVMPs from India. On the contrary, the use of *Lygodium flexuosum* (L.) Sw. root is very much new in respect of treating livestock diseases such as fever of goat (Jain, 1999; Katewa et al., 2010; Jain, 2012; Sikarwar, 2017).

Three fungal species such as *Amanita vaginata* var. *alba* Gillet, *Lycoperdon perlatum* Pers., and *Termitomyces heimii* Natarajan have been recorded as ethnoveterinary medicine used by the local traditional healers in the northern laterite region of West Bengal. Among these three fungal species, medicinal uses of *Amanita vaginata* var. *alba* Gillet and *Termitomyces heimii* Natarajan for curing veterinary diseases are exclusively the new addition to the existing database on ethnoveterinary medicine of India (Jain, 1999; Katewa et al., 2010; Jain, 2012; Jain and Jain, 2016).

The present study contributes 68 new medicinal claims, which is substantial and certainly enriches the existing inventories on ethnoveterinary medicine of India. Thus, the present investigation unveils the knowledge diversity of veterinary medicine in the study area and gives a clear indication regarding further studies exploring more novel information from the area after interacting with the traditional specialist healers of livestock diseases. All the new claims of EVMPs recorded here should scientifically be validated to develop bioactive compounds, and effective veterinary drugs have to be standardized after their toxicity assessment.

### 4.5 Informant Consensus and Cultural Value of Ethnoveterinary Medicinal Plants

Fic value (above 0.7) suggest a high consensus among the participants regarding the uses of large numbers of EVMPs in disease categories such as gastrointestinal disorders, poisonous effect, enteric diseases, fever, and related problems. All these livestock health problems are prevalent in the studied area, and local peoples’ understanding and perception of these health issues make them experts in disease diagnosis and prescribing effective folk remedies.

Fic is assigned to measure the consensus of participants regarding plant uses in a particular disease category, whereas the determination of the fidelity level (FL) helps to identify the most effective plant species cited for that particular disease category. Though sometimes, the FL value misleads in data interpretation when attaining a maximum score with few citations for one or two purposes. On the contrary, a species with multiple uses may show a lower FL value with more citations for a particular purpose. Therefore, it does not indicate that a plant with a higher fidelity percentage may have a maximal citation number. For this reason, in the present study, along with the FL value, the number of use mentions for a plant species made by all participants has been considered to recognize the most reliable species used in a disease category (Andrade-Cetto and Heinrich, 2011). A total of 23 species have been identified here as the most important medicinal plants whose FL value and citation number are higher than the other recorded plant species.

Among the frequently cited 68 EVM plant species, nine plants have been identified as the most valuable ones in the culture of the studied area, which indicate that the knowledge about uses of those nine plants is well distributed among the people of the area because of their higher frequency of citations as well as multipurpose uses, which are the basic components of the CV index.

The resulting value of the CV index is extracted from the cumulative effort of all the factors such as total number of use reports, total number of use categories, citation frequency, and the total number of participants interviewed. Therefore, the use of the CV index for assessing the cultural importance of a species is much more accurate than the individual application of indices such as UV, FL, or RFC, which are not independent of each other and function more or less similarly (Dudney et al., 2015). Thus, the scientific community should consider the CV index as an effective tool for assessing the overall cultural value of a species.

### 4.6 Conservation Facets

Quantitative analysis of ethnobotanical data not only helps identify the most important plant species but also provides information about those most frequently exploited plant species in a particular area, which will help frame a strategy for the conservation of those exploited plants. Among the important plants, some species that have been cited in a very high frequency are naturally facing a high collection pressure because of their use in more significant amounts than the other important species with lower citation frequency. Such species with the high use demand identified here are *Andrographis paniculata* (Burm.f.) Nees, *Aristolochia indica* L., *Soymida febrifuga* (Roxb.) A. Juss., *Madhuca longifolia* (J.Koenig ex L.) J.F.Macbr., *Asparagus racemosus* Willd., *Smilax ovalifolia* Roxb. ex D.Don, *Semecarpus anacardium* L.f., *Casearia tomentosa* Roxb., *Barleria prionitis* L., and *Acacia catechu* (L.f.) Willd. It is assumed that these species might face certain degrees of population decline shortly due to their excessive collection from the wild. Many other factors, such as unsustainable harvest of the bark (ring barking), underground part (uprooting of the whole plant), seed or fruit (indiscriminate collection), and habitat destruction, are also found responsible for the population decline of those most exploited plant species in the area. This fact has already been reflected in some phyto-sociological studies carried out in different forest areas of the northern laterite region of West Bengal, where very low populations of many of those above-mentioned plants were encountered (Joshi, 2012; Bauri et al., 2013; Bhattacharya and Mukherjee, 2013; Bouri et al., 2014; Pradhan and Rahaman, 2015; Ganguli et al., 2016). All those plants frequently used in the study area should get priority for their immediate conservation. In doing this, a separate research program has to be undertaken to identify the most prioritized species in the northern laterite part of West Bengal employing the well-devised dedicated statistical index like conservation priority index (CPI) or local conservation priority index (LCPI) (Oliveira et al., 2007; Lucena et al., 2013). In the present investigation, only the indication has been made toward population decline and collection pressure of the most frequently used medicinal plants in this region so that the researchers in the future can pursue their research activity in this direction.

### 4.7 Ethnopharmacological Rationalization of Most Important Ethnoveterinary Medicinal Plants

The detailed phytochemical and pharmacological screenings of the identified nine most valuable EVMPs should be prioritized for developing new bio-active constituents. Many of those nine culturally important plants have been screened earlier for their phytochemical and pharmacological properties. In many cases, pharmacological evidence of the earlier works validates the ethnomedicinal claims associated with those culturally important plants. *Amaranthus spinosus* L. is prescribed for treatment of delay in parturition, body ache, fever, hemorrhagic septicemia, and retention of milk and validated by earlier pharmacological studies for its antispasmodic (Chaudhary et al., 2012), antimicrobial (Sheeba et al., 2013), antioxidant, and antipyretic (Kumar B. S. A. et al., 2010) properties. This plant has not yet been examined for its galactagogue activity, which needs a thorough investigation to justify its traditional use as an enhancer of milk secretion in cows.

Pharmacological investigations on the anti-osteoporotic and anti-inflammatory activities of *Cissus quadrangularis* L. substantiate the scientific basis of using this plant to treat fractured bone and swelling wart (Kumar M. et al., 2010; Nalini et al., 2011; Stohs and Ray, 2013). Nevertheless, no scientific validation has been made for its anthelmintic property recorded in the present investigation.

The uses of *Wattakaka volubilis* (L.f.) Stapf. in liver trouble and unusual urination of the cattle recorded here need detailed phytochemical and pharmacological studies for its validation of hepatoprotective and diuretic properties as this medicinal plant has not been screened in such directions before (Natarajan and Dhas, 2013; Chaudhuri and Chakraborty, 2017).

In case of *Pueraria tuberose* (Willd.) DC., the tuber of it is used by the local people to treat helminthiasis and poor lactation. Many biological activities of *Pueraria* tuber have already been examined by different groups of scientists from various parts of the world, but no pharmacological investigations are made on its veterinary anthelmintic property (Hinsch et al., 2000; Saha et al., 2012; Chauhan et al., 2013).

Therefore, from this discussion, it is understood that these nine plants are culturally important and provide some important clues, enabling the scientists to undertake a scientific investigation for evaluating their phytochemical and pharmacological profiles.

### 4.8 Scientific Justification of Using Preferred Additional Ingredients

Preference ranking exercise of the additional ingredients used in polyherbal preparations revealed that ingredients such as fruits of *Piper nigrum* L. and *Piper longum* L., rhizomes of *Curcuma longa* L. and *Zingiber officinale* Roscoe, and seeds of *Nigella sativa* L. are the most popular among the participants of the studied area. There is a long tradition of using peppers (both black and long peppers) and ginger in many folk remedies. Scientific attempts have been made to justify the reason for using these herbal ingredients in traditional medicine. Through experiments, it has been established that the fruits of *Piper nigrum* L. and *Piper longum* L. contain piperine alkaloid, which increases the bioavailability of active principles present in a drug preparation (Patil et al., 2011). Scientists have suggested two possible mechanisms in this regard. Piperine may promote rapid absorption of drugs and nutrients through the intestine, and it also inhibits the activities of enzymes involved in the enzymatic breakdown of drugs (Ajazuddin et al., 2014). In the present investigation, *Piper longum* L. is used as an additional ingredient of a polyherbal formulation prepared with *Zingiber zerumbet* (L.) Roscoe ex Sm., *Zingiber officinale* Roscoe, and *Piper cubeba* L.f. to treat post-partum weakness. The use of *Piper longum* L. as a bioenhancer has already been established in a scientific study where it showed that the antiasthmatic effect of vasaka (*Adhatoda vasica* Nees) is increased when long pepper is added to it (Randhawa et al., 2011). Likewise, the traditional use of *Curcuma longa* L. in ethnomedicine has scientifically been justified through several studies where curcumin, its bioactive compound, has been established as a potent natural bioenhancer (Zhang et al., 2007; Pavithra et al., 2009; Yan et al., 2010). Besides its wide range of pharmacological efficacy, ginger (*Zingiber officinale* Roscoe) acts as an effective bioenhancer in promoting absorption of active phyto-constituents of the drug through the intestine (Qazi et al., 2003). Luteolin present in *Cuminum cyminum* L. exhibits its bioenhancing activity by inhibiting the activity of permeability glycoprotein (P-gp) present in the intestinal epithelium (Boumendjel et al., 2002). In a recent finding, it has been observed that active constituents of *Nigella sativa* L. interact with the co-administered drugs and enhance intestinal availability of the compounds present in the drugs (Ali et al., 2012). Therefore, the uses of all those herbal ingredients in polyherbal recipes as additional ingredients indicate that folk healers of the studied area have more excellent knowledge about different combinations of herbal ingredients in a traditional recipe, which helps enhance the effectiveness of the folk preparations.

### 4.9 Local Peoples’ Perception of Ethnoveterinary Medicinal Knowledge System and Its Resilience

EVM system is as old as the history of animal domestication, which is continuously being shaped and reshaped by trials and errors of using local biodiversity to maintain resilience. The resilience of the local EVM knowledge system depends on the utilization pattern of phytoresources and the knowledge transmission character (Santoro et al., 2015).

The present study witnessed some of the features of local EVM knowledge system which can play a crucial role in maintaining resilience such as men are much knowledgeable in practicing and applying EVM, most knowledgeable aged participants still possess substantial information of this therapeutic system, apart from the traditional expert practitioners of livestock diseases, a large number of participants have adequate EVM knowledge, and vertical transmission of knowledge is predominant. Local people of the laterite region of West Bengal depend mainly on cultivation and livestock rearing for their livelihood and by any sort of default the livestock population may decrease which creates a great bearing on their economy. For this reason people in the study area have been concerned to their livestock health care since ancient time and have been developed a well organized system of veterinary animal health care through regular incorporation of more and more newly innovated healing options for so many of ailments and diseases. A good number of EVM species have been used for single purpose (for example, 46 species of plants are used for gastro-intestinal problems of livestock) which is a good indicator of resilience of knowledge in the studied area because EVM system here does have a wide range of options for treatment of a particular disease, without being hampered due to unavailability of one or few drug plant resources.

## 5 Conclusion

The present study embodies a quite large extent of documented knowledge about 306 folk veterinary remedies which are worthy for its inclusion in the inventories on folk veterinary medicine and ethnomedicinal resources of the state and national level (Figure 7).

The uses of 68 EVMPs are new to the existing Indian ethnoveterinary pharmacopeia, which highlights the knowledge diversity and unknown knowledge on veterinary medicine in the surveyed area. Such new information create a golden opportunity in the field of bioprospecting research by providing the ethno-guided clues to the scientists for scientific validation, standardization, and safety evaluation of those plant species before their recommendation as ethnoveterinary medicine (EVM). Moreover, nine EVMPs have been identified as the most important species, which can also be considered statistically justified good candidates for their ethno-guided bioprospection in the future. The collaborative efforts of traditional and modern knowledge are needed here to develop new efficacious drugs for livestock diseases with minimum or zero side effects.

The present investigation highlights some basic concern about conservation status and collection pressure of those important ethno-species used most frequently in the area. To prioritize the most exploited species for conservation in the area, along with collection pressure faced by each species, other factors like, degree of access and population dynamics of each of the important species are to be considered.

The strength of the EVM system identified in the region is its knowledge diversity (recorded remedies 306) and diversity of associated drug resources including phytoresources (plant species recorded 232). The system is practiced among the local people in the area very actively with a good number of optional drug species assigned to the healing purposes of many common diseases. These characteristics of the EV knowledge articulate its vitality and also the flexibility of its many of the knowledge spheres. Knowledge transmission is operated here predominantly through vertical route that is also an indication of resilience of the EVM system. Besides, the greater part of this vast knowledge trove is confined to the aged people domain, not to the younger generation in the society of this area. This is a very alarming concern identified in the context of sustainability of EVM knowledge system in the northern laterite region of West Bengal, India.

## Data Availability

The original contributions presented in the study are included in the article/Supplementary Material. Further inquiries can be directed to the corresponding authors.

## Footnotes

1
http://www.worldfloraonline.org retrieved on 10.02.2022.

2
http://www.theplantlist.org/retrieved on 05.01.2022.

3
http://www.tropicos.org/retrieved on 26.08.2021.

4
http://www.ars-grin.gov retrieved on 18.09.2021.
