# Supplementary material for: Inventorization and Consensus Analysis of Ethnoveterinary Medicinal Knowledge Among the Local People in Eastern India: Perception, Cultural Significance, and Resilience
Source: Front Pharmacol. 2022 Apr 29;13:861577. doi: 10.3389/fphar.2022.861577 (PMC9099233; doi:10.3389/fphar.2022.861577)
Supplement: Supplementary file 1 [file Table4.pdf]

Supplementary Table S4. Enumeration of EVMPs recorded from the study area found new after perusal of the previous studies from India

**Category of new uses**	**Scientific name of the plant species**	**Used for**	**Comparison with previous study from India**
Exclusively new	*Abutilon hirtum* (Lam.) Sweet	Suppurating wound	Reported EVMPs are exclusively new for India as they have not been reported in the standard literature consulted (Pal and Jain, 1998; Jain, 1999; Ghosh, 2003; Rahaman et al., 2009; Katewa et al., 2010; Jain, 2012; Kumar et al., 2012; Saha et al., 2014; Jain and Jain, 2016; Sikarwar, 2017)
	Aerva javanica (Burm. f.) Juss. ex Schult.	Bone fracture	
	Albizia procera (Roxb.) Benth.	Diarrhoea	
	Coleus strobilifer (Roxb.) A.J.Paton	Cough	
	Cajanus goensis Dalzell	Fever	
	Breynia vitis-idaea (Burm.f.) C.E.C.Fisch.	Body swelling due to cold	
	Caladium bicolor (Aiton) Vent.	Swelling wart	
	Centipeda minima (L.) A.Braun & Asch.	Rhinorrhoea	
	Croton persimilis Müll. Arg.	Poor lactation	
	Cotula anthemoides L.	Watering of eyes	
	Eulophia explanata Lindl.	Lameness of the hind leg	
	Hydrolea zeylanica (L.) Vahl	Wound	
	Ipomoea cairica (L.) Sweet	Helminthiasis (with stomach ache)	
	Ipomoea obscura (L.) Ker.-Gowl.	Broken horn	
	Jatropha nana Dalzell & A.Gibson	Retention of milk	
	Ludwigia adscendens (L.) H.Hara	Infertility	
	Phoenix acaulis Roxb.	Retention of milk (post-parturition)	
		Dystocia (difficulty in parturition)	
	Phyllodium pulchellum (L.) Desv.	Post parturition bleeding	
	Piper cubeba L. f.	Post-partum weakness	
	Rotheca serrata (L.) Steane & Mabb.	Fever	
	Seseli diffusum (Roxb. ex Sm.) Santapau &Wagh	Urinary incontinence	
	Tacca leontopetaloides (L.) Kuntze	Diarrhoea	
	Uraria lagopodiodes (L.) DC.	Diarrhoea	
	Zingiber zerumbet (L.) Roscoe ex Sm.	Gut erosion, pulmonary congestion, foot rot and any kind of poisoning of the cattle	

New in respect of the diseases cured	*Ouret lanata* (L.) Kuntze	Foot and mouth diseases	Root- poisonous bite (Jain, 1999; Katewa et al., 2010), poor lactation (Jain, 1999), constipation (Katewa et al., 2010); Leaf- hemorrhagic gastroenteritis, dysentery and poisonous bite (Bhuvaneswari et al., 2015)
	Agave americana L.	Inflammatory swelling in shoulder	Leaf- broken horn (Katewa et al., 2010), wound (Pragada and Rao, 2012), bone fracture (Rajkumari et al., 2014)
	Alangium salviifolium (L.f.) Wangerin	Weakness/ poor health	Stem bark- cough and poisonous bite (Jain, 1999); root- dog bite, leaf- malarial fever and enlargement of spleen (Katewa et al., 2010)
	Alstonia scholaris (L.) R. Br.	Inflammatory swelling in shoulder	Root- anorexia, intestinal worm (Saha et al., 2014); bark- fever (Jain, 1999; Bharati and Sharma, 2010), gastric problems (Pragada and Rao, 2012), intestinal worm (Jain, 1999); seed- weakness, latex- dysentery; leaf- wound and sore (Jain, 1999)
	Biophytum sensitivum (L.) DC.	Stop mastication	Leaf- cut wounds (Jain, 1999; Katewa et al., 2010), whole plant- poor lactation (Sadangi and Sahu, 2004)
	Careya arborea Roxb.	Opacity of cornea	Stem bark- diarrhea and dysentery, wound (Jain, 1999); leaf- wound (Sikarwar and Kumar, 2005); leaf- dislocated joints (Harsha et al., 2005)
	Casearia tomentosa Roxb.	Cattle diarrhoea	Stem bark- urinary trouble, wound, ulcer (Jain, 1999), dysentery (Murthy et al., 2007); whole plant- Snake bite (Jain, 1999); leaf- body pain and swelling due to contusion (Punjani and Pandey, 2015)
	Curcuma aromatica Salisb.	Food poisoning	Rhizome- scabies (Jain, 1999; Sharma and Sapcota, 2003), internal worms (Jagadeeswary et al., 2014), mastitis (Dhanam and Elayaraj, 2014; Manoranjotham and Kamaraj, 2016)
	Cuscuta reflexa Roxb.	Food poisoning	Whole plant- poor lactation (Bandyopadhyay and Mukherjee, 2005; Pragada and Rao, 2012), bone fracture, lice (Jain, 1999), wound due to worm bite (Yadav et al., 2014)
	Pleurolobus gangeticus (L.) J.St.-Hil. ex H.Ohashi & K.Ohashi	Hemorrhagic septicemia, bloody dysentery	Root- prevent death of foetous (Jain, 1999)
	Drosera burmanni Vahl	Infectious diseases	Root- wound (Jain, 1999)
	Euphorbia antiquorum L.	Opacity of cornea	Latex and stem- wound, stem- anthrax and leg swelling (Jain, 1999); latex- fractured bone (Lakshminarayana and Rao, 2013); latex- lameness (Kannan et

			al., 2016)
	Euphorbia fusiformis Buch.-Ham. ex D. Don	Retention of milk	Tuberous root- ephemeral fever (Reddy et al., 2006), dysentery and fever (Jain, 1999; Sikarwar and Kumar, 2005)
	Mesosphaerum suaveolens (L.) Kuntze	Fresh cuts and wounds	Leaf- conjunctivitis (Jain, 1999; Satapathy, 2010)
	Justicia gendarussa Burm.f.	Helminthiasis	Leaf- fracture of bone (Jain, 1999), dysentery (Reang et al., 2016; Mehmud, 2017); indigestion and constipation (Reang et al., 2016)
	Leea asiatica (L.) Ridsdale	Food poisoning	Root- wound (Jain, 1999), eye disease (Pande et al., 2007), fracture (Nair et al., 2017)
	Litsea glutinosa (Lour.) C. B. Rob.	Loose motion	Bark- bone fracture (Jain, 1999; Bharali et al., 2015), wound (Jain, 1999), urinary trouble (Bharali et al., 2015); leaf- stomach problems (Jain, 1999); root- bone fracture (Saha et al., 2014)
	Murraya koenigii (L.) Spreng.	Drowsiness of small ruminants	Leaf- diarrhoea (Pragada and Rao, 2012), rinderpest, infertility (Jain, 1999)
	Musa paradisiaca L.	Haematuria	Poor lactation, gastropathies, cholera (Jain, 1999), leaf and fruit- diarrhoea, fruit- ulcer, stem- haemorrhage (Jain, 1999); leaf- retention of placenta, fruit- swelling of udder and mastitis (Katewa et al., 2010)
	Nicotiana rustica L.	Maggot infested wound	Leaf and seed- expel leech (Jain, 1999)
	Papaver somniferum L.	Bloody dysentery	Leaf- urinary disorders, latex- poisoning, sprain (Jain, 1999), seed- nervous system disorders (Pragada and Rao, 2012), gastroenteritis (Bhuvaneswari et al., 2015)
	Polygala arvensis Willd.	Listeriosis	Leaf- poisonous bite (Reddy et al., 2016; Nair et al., 2017)
	Pueraria tuberosa (Willd.) DC.	Helminthiasis	Tuber- reproductive disorder, intestinal disorder, weakness (Jain, 1999) poor lactation (Jain, 1999; Sikarwar and Kumar, 2005; Jain, 2012), post-partum weakness (Katewa et al., 2010), neck swelling (Panda and Dhal, 2014)
	Schoenoplectiella articulata (L.) Lye	Wound	Stem- poor lactation (Panda et al., 2017)

	Scoparia dulcis L.	Fever, retention of urine, retention of placenta, stomachache	Whole plant- stop mastication, dysentery (Jain, 1999), gout (Jain, 1999; Dey and De, 2010)
	Sida cordifolia L.	Loose motion	Whole plant- shivering disease (Jain, 1999); leaf- rheumatism (Reddy et al., 2016)
	Solanum torvum Sw.	Bloat	Fruit- diarrhoea, respiratory trouble (Jain, 1999); whole plant- bone fracture (Pushpangadan et al., 2016)
	Tragia involucrata L.	Fever	Root- haemorrhage (Jain, 1999), flies problems (Saha et al., 2014); leaf- dysentery (Jain, 1999)
	Tribulus terrestris L.	Bloat	Seed- retention of placenta (Jain, 1999); whole plant- diarrhoea (Yadav et al., 2014), leaf- cough (Verma, 2014); fruit- diarrhoea, shoot- intestinal parasites, root- external parasites (Katewa et al., 2010)
	Typhonium trilobatum (L.) Schott	Swelling of wart	Root- inflammation (Jain, 1999), maggot infested wounds (Mehmud, 2017)
	Ziziphus nummularia (Burm.f.) Wight & Arn.	Rhinorrhoea	Root- delivery related problems, injury (Jain, 1999); thorn- retention of placenta, roo- labour pain, whole plant- poor lactation (Katewa et al., 2010)
New in respect of remedy preparation modes	*Achyranthes aspera* L.	Cattle fever	Previously, for the treatment of cattle fever juice of roots of *Achyranthes aspera* and *Leonurus japonicas*; oral (Saha et al., 2014). But here paste prepared from root of Achyranthes aspera and seeds of *Nigella sativa*, and administered orally mixed with mucilaginous extract of *Aloe vera* leaf (locally known as "*Musabbar*").
	Nicotiana rustica L.	Wound	Leaf juice applied topically to treat wound (Jain, 1999) but here a mixture of *Nicotiana* leaf paste, mustard oil and 'Sankhachurna' is applied as poultice on the wound.
	Leonotis nepetifolia (L.) R.Br.	Mastitis	Root paste of only *L. nepetifolia* is used as poultice to treat mastitis (Jain, 1999; Narayana and Rao, 2013). In the present study, root of *L. nepetifolia* is made into paste along with the root of *Abrus precatorius* (2:1) and applied as poultice on the mammary gland.
	Calotropis gigantea (L.) W.T.Aiton	Arthritis	For the treatment of cattle arthritis, use of only the leaf paste of *Calotropis gigantea* is reported earlier (Sudarsanam et al., 1995).<br>Here, 14-15 pieces of mature *Calotropis* leaves are made into paste and mixed with 5 gm powder of Ammonium

			chloride ("Nishadal"), fecal matter of a heifer (500 gm) and required amount of soil from the mouth of crab hole. All the ingredients are taken into an earthen pot, heated for few minutes and applied all over the paralyzed leg twice a day till the cure.
	Abrus precatorius L.	Dysentery	Whole plant is given orally to treat dysentery (Bhuvaneswari et al., 2015). In the present study, 4-5 pieces of Abrus seeds are made into paste along with 100 gm bamboo leaves and little amount of feather of Indian roller, ("Nilkantha" bird), and applied orally.
New in respect of the parts used	*Abutilon indicum* (L.) Sweet	Watering of eyes (Root)	Leaf - lice, eye disease (Jain, 1999); dysentery (Jain, 1999; Selvaraju et al., 2011; Bhuvaneswari et al., 2015) and anorexia (Bhuvaneswari et al., 2015)
	Echinops echinatus Roxb.	Sore on shoulder (Whole plant)	Root- lice (Jain, 1999); wound (Sikarwar and Kumar, 2005); and poor lactation (Chouhan and Ray, 2015)
	Oroxylum indicum (L.) Kurz	Mastitis (Flower)	Bark- sore, bone fracture (Jain, 1999), crack in nipple (Saha et al. 2014), wound (Sharma and Sapcota, 2003); leaf –dysentery (Jain, 1999); fruit and bark- wound (Bharali et al., 2015); seed – wound or crack in nipple (Jain, 1999; Satapathy, 2010; Panda et al., 2017)
	Ziziphus jujuba Mill	Retention of placenta (Tender shoot)	Bark and leaf- diarrhea and miscarriage (Jain, 1999); leaf- skin burn (Nigam and Sharma, 2010; Verma, 2014); leaf- dysentery, fruit- constipation (Kumar and Nagayya, 2017)
