# Supplementary material for: Inventorization and Consensus Analysis of Ethnoveterinary Medicinal Knowledge Among the Local People in Eastern India: Perception, Cultural Significance, and Resilience
Source: Front Pharmacol. 2022 Apr 29;13:861577. doi: 10.3389/fphar.2022.861577 (PMC9099233; doi:10.3389/fphar.2022.861577)
Supplement: Supplementary file 2 [file Table1.pdf]

Supplementary Table S1. Socio-demographic profile of the participants (n = 132)

**Variables**	**Categories**	**Numbers**	**Percentage (%)**
Gender	Male	109	82.58
	Female	23	17.42
Social category	Schedule tribe	79	59.85
	Schedule cast	32	24.24
	Other Backward Class	03	2.27
	General	18	13.64
Age group (years)	< 30	9	6.82
	30-49	32	24.24
	50-69	63	47.73
	> 70	28	21.21
Education	Without formal education	71	53.79
	Primary level	34	25.76
	Secondary level	17	12.88
	Higher education (University/college)	11	8.33
Experience (years)	5 – 10	12	9.09
	11 – 25	39	29.55
	26 – 50	55	41.67
	> 50	26	19.7
Residence	Rural	82	62.12
	Semi-urban	34	25.76
	Urban	16	12.12
Principal Knowledge acquiring source	Family member	74	56.06
	Outside the family (knowledgeable person or a traditional practitioner)	56	42.42
	Keen observation	02	1.52
