# Supplementary material for: Inventorization and Consensus Analysis of Ethnoveterinary Medicinal Knowledge Among the Local People in Eastern India: Perception, Cultural Significance, and Resilience
Source: Front Pharmacol. 2022 Apr 29;13:861577. doi: 10.3389/fphar.2022.861577 (PMC9099233; doi:10.3389/fphar.2022.861577)
Supplement: Supplementary file 3 [file Table2.pdf]

Supplementary Table S2. List of the recorded plant species used as ethnoveterinary medicine in the northern laterite region of West Bengal, India (n = 232)

Scientific name of the EVM Plant with voucher specimen number	Local name and common name	Family	Habit
**1.** *Abelmoschus moschatus* Medik. SKM12	বন-ভেন্ডি<br>Musk okra	*Malvaceae*	Herb
**2.** *Abrus precatorius* L. SKM53	কুঁচ<br>Rosary pea	*Fabaceae*	Climber
**3.** *Abutilon hirtum* (Lam.) Sweet SKM34	জোখা<br>Indian mallow	*Malvaceae*	Shrub
**4.** *Abutilon indicum* (L.) Sweet SKM01	টেপুর<br>Monkey bush	*Malvaceae*	Shrub
**5.** *Achyranthes aspera* L. SKM85	চিড়চিড়ি<br>Prickly chaff flower	*Amaranthaceae*	Herb
**6.** *Acorus calamus* L. SKM819	বচ<br>Sweet flag	*Acoraceae*	Herb
**7.** *Adiantum philippense* subsp. *philippense*<br>Syn.- *Adiantum lunulatum* Burm. f. SKM90	কালীঝাঁপ<br>Maidenhair fern	*Pteridaceae*	Herb
**8.** *Adina cordifolia* (Roxb.) Brandis<br>Syn.- *Haldina cordifolia* (Roxb.) Ridsdale SKM912	কেলি-কদম<br>Heart-leaf adina	*Rubiaceae*	Tree
**9.** *Aegle marmelos* (L.) Corrêa SKM101	বেল<br>Stone apple	*Rutaceae*	Tree
**10.** *Aerva javanica* (Burm.f.) Juss. ex Schult. SKM21	লাল-পাতা, বশল্যকরনী<br>Pillow-weed	*Amaranthaceae*	Herb
**11.** *Agave americana* L. SKM23	পাত-কোঙা<br>Century plant	*Asparagaceae*	Shrub
**12.** *Alangium salviifolium* (L.f.) Wangerin SKM145	আঁকড়, ভাদুভিলি<br>Sage-leaved alangium	*Cornaceae*	Tree
**13.** *Albizia procera* (Roxb.) Benth. SKM512	সাদা শিরীষ<br>White Siris	*Fabaceae*	Tree
**14.** *Allium sativum* L. SKM17	রসুন<br>Garlic	*Amaryllidaceae*	Herb
**15.** *Aloe vera* (L.) Burm.f. SKM40	ঘৃতকুমারী<br>Aloe	*Asphodelaceae*	Herb
**16.** *Alstonia scholaris* (L.) R. Br. SKM918	ছাতিম<br>Devil's tree	*Apocynaceae*	Tree
**17.** *Amanita vaginata* var. *alba* Gillet SKM520	বুধি-ওতে, শাল ছাতু<br>Grisette amanita	*Amanitaceae*	Fruit body
**18.** *Amaranthus spinosus* L. SKM43	কাঁটানটে<br>Spiny amaranth	*Amaranthaceae*	Herb
**19.** *Ampelocissus latifolia* (Roxb.) Planch. SKM213	ইজের, আঙুর-লতা<br>Wild Grape	*Vitaceae*	Climber
**20.** *Ananas comosus* (L.) Merr. SKM323	আনারস<br>Pineapple	*Bromeliaceae*	Herb
**21.** *Andrographis paniculata* (Burm.f.) Nees SKM25	ভুঁই-নিম, কালমেঘ<br>Creat	*Acanthaceae*	Herb

**22.** *Annona squamosa* L. SKM64	আতা<br>Custard Apple	*Annonaceae*	Tree
**23.** *Argemone mexicana* L. SKM28	শিয়াল-কাঁটা<br>Mexican prickly poppy	*Papaveraceae*	Herb
**24.** *Argyreia nervosa* (Burm. f.) Bojer SKM832	মারাঙ-হারু, ভিরিদক-আরা<br>Elephant Climber	*Convolvulaceae*	Climber
**25.** *Aristolochia indica* L. SKM63	ঈশ্বরমূল, গঁদ<br>Indian Birthwort	*Aristolochiaceae*	Climber
**26.** *Artocarpus heterophyllus* Lam. SKM103	কাঁঠাল<br>Jackfruit	*Moraceae*	Tree
**27.** *Asparagus racemosus* Willd. SKM134	শতমূল<br>Shatavari	*Asparagaceae*	Climber
**28.** *Azadirachta indica* A. Juss. SKM32	নিম<br>Neem	*Meliaceae*	Tree
**29.** *Azanza lampas* (Cav.) Alef. SKM435	বন কাপাস<br>Common mallow	*Malvaceae*	Shrub
**30.** *Baccharoides anthelmintica* (L.) Moench SKM36<br>Syn.- *Centratherum anthelminticum* (L.) Gamble	সোমরাজ<br>Ironweed	*Asteraceae*	Herb
**31.** *Bambusa bambos* (L.) Voss. SKM09<br>Syn.- *B. aurandinacea* Willd.	বাঁশ<br>Indian thorny bamboo	*Poaceae*	Tree
**32.** *Barleria prionitis* L. SKM418	দাঁতরেশে<br>Dog bush	*Acanthaceae*	Shrub
**33.** *Bauhinia acuminata* L. SKM739	শ্বেত-কাঞ্চন<br>White Bauhinia	*Fabaceae*	Tree
**34.** *Biophytum sensitivum* (L.) DC. SKM640	ঝালে<br>Little tree plant	*Oxalidaceae*	Herb
**35.** *Blumea lacera* (Burm.f.) DC. SKM141	কুকশিমে<br>Blumea	*Asteraceae*	Herb
**36.** *Boerhavia diffusa* L. SKM165	কিচু-আরা<br>Red Spiderling	*Nyctaginaceae*	Herb
**37.** *Bombax ceiba* L. SKM44	শ্বেত-শিমুল<br>Silk cotton tree	*Malvaceae*	Tree
**38.** *Brassica nigra* (L.) W.D.J.Koch SKM26	সরষে<br>Black mustard	*Brassicaceae*	Herb
**39.** *Breynia vitis-idaea* (Burm.f.) C.E.C.Fisch. SKM27	পান-শাওরা<br>Indian snowberry	*Phyllanthaceae*	Shrub
**40.** *Cajanus goensis* Dalzell SKM924<br>Syn.- *Atylosia goensis* (Dalzell) Dalzell	বন-বিচিতি	*Fabaceae*	Climber
**41.** *Cajanus scarabaeoides* (L.) Thouars SKM584<br>Syn.- *Atylosia scarabaeoides* (L.) Benth.	বিরহোরে, ওলাঙ-ওচে-আরা<br>Showy pigeonpea	*Fabaceae*	Climber
**42.** *Caladium bicolor* (Aiton) Vent. SKM94	নির্বিষ<br>Heart of Jesus	*Araceae*	Herb

**43.** *Calotropis gigantea* (L.) W.T.Aiton SKM73	শ্বেত-আকন্দ<br>Giant Milkweed	*Apocynaceae*	Shrub
**44.** *Calotropis procera* (Aiton) W.T. Aiton SKM79	আকন্দ<br>Sodom apple	*Apocynaceae*	Shrub
**45.** *Capparis sepiaria* L. SKM215	কাঁঠাড়ি<br>Hedge caper	*Capparaceae*	Climber
**46.** *Capparis zeylanica* L. SKM39	কালিকোঁরা<br>Ceylon Caper	*Capparaceae*	Climber
**47.** *Capsicum annuum* L. SKM127<br>Syn.- *Capsicum frutescens* L.	লঙ্কা<br>Chili	*Solanaceae*	Shrub
**48.** *Cardiospermum halicacabum* L. SKM456	লাইপুটকি<br>Balloon Vine	*Sapindaceae*	Climber
**49.** *Careya arborea* Roxb. SKM656	কুম্ভি<br>Wild guava	*Lecythidaceae*	Tree
**50.** *Carica papaya* L. SKM19	পেঁপে<br>Papaya	*Caricaceae*	Tree
**51.** *Carissa spinarum* L. SKM873	বন করমচা<br>Bush plum	*Apocynaceae*	Shrub
**52.** *Carum carvi* L.	সা-জিরে<br>Caraway	*Apiaceae*	Herb
**53.** *Casearia tomentosa* Roxb. SKM693<br>Syn.- *Casearia elliptica* Willd.	চরচি-দারি<br>Toothed Leaf Chilla	*Salicaceae*	Tree
**54.** *Catunaregam spinosa* (Thunb.) Tirveng. SKM681<br>Syn.- *Randia spinosa* (Thunb.) Poir.	লোতো<br>Mountain pomegranate	*Rubiaceae*	Shrub
**55.** *Causonis trifolia* (L.) Mabb. & J.Wen<br>*Syn.- Cayratia trifolia* (L.) Domin SKM95	গোয়ালে-লতা, গোর-লতা<br>Three-Leaved Wild Vine	*Vitaceae*	Climber
**56.** *Celastrus paniculatus* Willd. SKM312	কুজরী<br>Black Oil Plant	*Celastraceae*	Climber
**57.** *Centipeda minima* (L.) A.Braun & Asch. SKM236	মেচেতা<br>Spreading Sneeze Weed	*Asteraceae*	Herb
**58.** *Chamaecrista mimosoides* (L.) Greene SKM347<br>Syn.- *Cassia mimosoides* L.	ওত-কোন্দরো<br>Feather-leaved Cassia	*Fabaceae*	Herb
**59.** *Chenopodium album* L. SKM477	বেথো-আরা<br>Goose foot	*Amaranthaceae*	Herb
**60.** *Chrysopogon zizanioides* (L.) Roberty SKM536<br>Syn.- *Vetiveria zizanioides* (L.) Nash	বেনা-ঘাস<br>Vetiver grass	*Poaceae*	Herb
**61.** *Cissampelos pareira* L. SKM54	একনাভি<br>Velvet Leaf	*Menispermaceae*	Climber
**62.** *Cissus quadrangularis* L. SKM37	হাড়জোড়া<br>Devil's backbone	*Vitaceae*	Climber

**63.** *Cleome gynandra* L. SKM991	হুরহুরে<br>Cat's whiskers	*Cleomaceae*	Herb
**64.** *Clerodendrum indicum* (L.) Kuntze SKM88	বামনহাটী<br>Tube flower	*Lamiaceae*	Shrub
**65.** *Clerodendrum infortunatum* L. SKM08<br>Syn.- *Clerodendrum viscosum* Vent.	ঘেঁটু, ভাঁট<br>Hill glory bower	*Lamiaceae*	Shrub
**66.** *Coccinia grandis* (L.) Voigt SKM191	কিদুরি, তেলাকুচি<br>Ivy gourd	*Cucurbitaceae*	Climber
**67.** *Cocculus hirsutus* (L.) W.Theob. SKM458	আরাক-আন-আরা<br>Broom creeper	*Menispermaceae*	Climber
**68.** *Coix lacryma-jobi* L. SKM163	গর-গন্ডা<br>Job's tears	*Poaceae*	Herb
**69.** *Coleus strobilifer* (Roxb.) A.J.Paton SKM826<br>*Syn.- Anisochilus carnosus* (L.f.) Wall. ex Benth.	পানজিরি পাতা<br>Thick-Leaf Lavender	*Lamiaceae*	Herb
**70.** *Colocasia esculenta* (L.) Schott SKM76<br>*Syn.- Colocasia antiquorum* Schott	জলের কচু, আঁজা<br>Taro	*Araceae*	Herb
**71.** *Coriandrum sativum* L. SKM78	ধনিয়া, ধনে<br>Coriander	*Apiaceae*	Herb
**72.** *Cotula anthemoides* L. SKM266	বারাঙ্গো<br>Button weed	*Asteraceae*	Herb
**73.** *Crinum asiaticum* L. SKM179	বন পিঁয়াজ<br>Sea shore Lily	*Amaryllidaceae*	Herb
**74.** *Crotalaria quinquefolia* L. SKM353<br>(Syn.- *Crotalaria heterophylla* L.f.)	বন ঝুনকা<br>Five Leaf Rattlepod	*Fabaceae*	Herb
**75.** *Croton persimilis* Müll. Arg. SKM189<br>Syn.- *Croton oblongifolius* Roxb.	টস কাটি<br>Croton Tree	*Euphorbiaceae*	Tree
**76.** *Cuminum cyminum* L.	মৌরী<br>Cumin	*Apiaceae*	
**77.** *Curculigo orchioides* Gaertn. SKM62	তারমুলি, তালমূল<br>Golden eye-grass	*Hypoxidaceae*	Herb
**78.** *Curcuma aromatica* Salisb. SKM86	বন হলুদ<br>Wild turmeric	*Zingiberaceae*	Herb
**79.** *Curcuma longa* L. SKM20	হলুদ<br>Turmeric	*Zingiberaceae*	Herb
**80.** *Cuscuta reflexa* Roxb. SKM919	হাঁড়ু-মালা, স্বর্নলতা<br>Giant dodder	*Convolvulaceae*	Climber
**81.** *Cyanotis tuberosa* (Roxb.) Schult. & Schult.f. SKM11	কাশমূলি<br>Sahyadri Dew-Grass	*Commelinaceae*	Herb
**82.** *Cynodon dactylon* (L.) Pers. SKM122	দূর্বা-ঘাস<br>Bermuda grass	*Poaceae*	Herb
**83.** *Cyperus rotundus* L. SKM121	মুথো-ঘাস<br>Java grass	*Cyperaceae*	Herb
**84.** *Datura stramonium* L. SKM114	ধুতরো	*Solanaceae*	Shrub

	Thorn apple		
**85.** *Dendrolobium triangulare* (Retz.) Schindl. SKM678<br>Syn.- *Desmodium triangulare* (Retz.) Merr.	চপাতে-আরা<br>Triangular Horse Bush	*Fabaceae*	Shrub
**86.** *Dendrophthoe falcata* (L.f.) Ettingsh. SKM92	সম-আরা<br>Long-leaved Mistletoe	*Loranthaceae*	Herb
**87.** *Dillenia pentagyna* Roxb. SKM265	বন চালতা, করকট<br>Dog Teak	*Dilleniaceae*	Tree
**88.** *Dioscorea bulbifera* L. SKM268	বন আলু, পিসকা<br>Air yam	*Dioscoreaceae*	Climber
**89.** *Dracaena angolensis* (Welw. ex Carrière) Byng & Christenh. SKM110<br>Syn.- *Sansevieria cylindrica* Bojer ex Hook.	মহাদেব-জটা<br>Spear Sansevieria	*Asparagaceae*	Herb
**90.** *Drosera burmanni* Vahl SKM324	সূর্যশিশির, চাঁদলৈ<br>Tropical sundew	*Droseraceae*	Herb
**91.** *Echinops echinatus* Roxb. SKM421	টান্ডি-জেনুম<br>Indian globe thistle	*Asteraceae*	Herb
**92.** *Eclipta prostrata* (L.) L. SKM13	কেসুতে<br>False daisy	*Asteraceae*	Herb
**93.** *Enydra fluctuans* Lour. SKM422	হঞ্চে<br>Buffalo Spinach	*Asteraceae*	Herb
**94.** *Eulophia explanata* Lindl. SKM200	গাইহামলা<br>Flattened Eulophia	*Orchidaceae*	Herb
**95.** *Euphorbia antiquorum* L. SKM302	তেশিরা, এতকে<br>Triangular Spurge	*Euphorbiaceae*	Tree
**96.** *Euphorbia fusiformis* Buch.-Ham. ex D. Don SKM93	দুধমূলো<br>Pillpod spurge	*Euphorbiaceae*	Herb
**97.** *Euphorbia hirta* L. SKM147	পুশিতোয়া<br>Hairy Spurge	*Euphorbiaceae*	Herb
**98.** *Euphorbia neriifolia* L. SKM229	মনসা-সিজ<br>Indian Spurge Tree	*Euphorbiaceae*	Tree
**99.** *Ferula assa-foetida* L.	হিং<br>Asafoetida	*Apiaceae*	Herb
**100.** *Ficus benghalensis* L. SKM06	বট<br>Indian banyan	*Moraceae*	Tree
**101.** *Ficus racemosa* L. SKM109<br>Syn.- *Ficus glomerata* Roxb.	যজ্ঞ ডুমুর<br>Cluster fig	*Moraceae*	Tree
**102.** *Ficus religiosa* L. SKM82	পাকুর<br>Sacred Fig Tree	*Moraceae*	Tree
**103.** *Gardenia latifolia* Aiton SKM1113	পাপরো<br>Indian Boxwood	*Rubiaceae*	Tree

**104.***Glochidion multiloculare* (Rottler ex Willd.) Voigt SKM1123	রাটিন	*Phyllanthaceae*	Shrub
**105.***Gloriosa superba* L. SKM908	অগ্নিশিখা, সিমিঙ-সাম-আরা<br>Flame lily	*Colchicaceae*	Climber
**106.***Grona triflora* (L.) H.Ohashi & K.Ohashi<br>Syn.- *Desmodium triflorum* (L.) DC. SKM197	কোদালে-কুরালে<br>Three-flower tick-trefoil	*Fabaceae*	Herb
**107.***Guilandina bonduc* L.<br>*Syn.- Caesalpinia bonduc* (L.) Roxb. SKM174	ঝগরেটে ফল<br>Grey nicker	*Fabaceae*	Shrub
**108.***Helicteres isora* L. SKM817	আতমরা<br>Indian screw tree	*Malvaceae*	Shrub
**109.***Hellenia speciosa* (J.Koenig) S.R.Dutta SKM547<br>*Syn.- Cheilocostus speciosus* (J.Koenig) C.D. Specht<br>Syn.- *Costus speciosus* (J.Koenig) Sm.	টিয়া<br>Crepe Ginger	*Costaceae*	Herb
**110.***Hemidesmus indicus* (L.) R.Br. SKM827	অনন্তমূল<br>Indian sarsaparilla	*Apocynaceae*	Climber
**111.***Hibiscus cannabinus* L. SKM1133	মেচা, বামবোরা<br>Deccan hemp	*Malvaceae*	Herb
**112.***Holarrhena pubescens* Wall. ex G.Don SKM03<br>Syn.- *H. antidysenterica* (L.) Wall. ex A. DC.	কুর্চি, ইন্দ্রযব<br>Bitter Oleander	*Apocynaceae*	Tree
**113.***Hydrolea zeylanica* (L.) Vahl SKM549	একলাঙ্গুলে, লঙ্গুল<br>Ceylon Hydrolea	*Hydroleaceae*	Herb
**114.***Ipomoea cairica* (L.) Sweet SKM239	লঙ্গুলী লতা<br>Messina Creeper	*Convolvulaceae*	Climber
**115.***Ipomoea carnea* Jacq. SKM137	বেড়া কলমী<br>Bush Morning Glory	*Convolvulaceae*	Shrub
**116.***Ipomoea obscura* (L.) Ker Gowl. SKM457	ভাচা-আরা<br>Obscure Morning Glory	*Convolvulaceae*	Climber
**117.***Jatropha curcas* L. SKM373	শ্বেত-ভেরেন্ডা<br>Physic nut	*Euphorbiaceae*	Shrub
**118.***Jatropha gossypiifolia* L. SKM192	লাল ভেরেন্ডা, জামালকোটা<br>Bellyache bush	*Euphorbiaceae*	Shrub
**119.***Jatropha nana* Dalzell & A.Gibson SKM151	বীর-এরাডম<br>Dwarf Jatropha	*Euphorbiaceae*	Herb
**120.***Justicia adhatoda* L. SKM184<br>Syn.- *Adhatoda vasica* Nees	বাসক<br>Malabar nut	*Acanthaceae*	Shrub
**121.***Justicia gendarussa* Burm.f. SKM339	কালো চাঁদোয়া<br>Willow-leaved Justicia	*Acanthaceae*	Shrub
**122.***Kalanchoe pinnata* (Lam.) Pers. SKM55<br>Syn.- *Bryophyllum pinnatum* (Lam.) Oken	পাথরকুচী, চৌডল<br>Cathedral bells	*Crassulaceae*	Herb

**123.***Lawsonia inermis* L. SKM657	মেহেন্দী, হেনা<br>Henna tree	*Lythraceae*	Tree
**124.***Leea asiatica* (L.) Ridsdale SKM561	সাহার-দারি<br>Asiatic Leea	*Vitaceae*	Shrub
**125.***Leonotis nepetifolia* (L.) R.Br. SKM638	ভুতভৈরব, দারে-ধুম্পো<br>Christmas candlestick	*Lamiaceae*	Herb
**126.***Leucas cephalotes* (Roth) Spreng. SKM15	গলঘসে<br>Spider wort	*Lamiaceae*	Herb
**127.***Linum usitatissimum* L.	মসনে<br>Linseed	*Linaceae*	Herb
**128.***Lippia javanica* (Burm.f.) Spreng. SKM676	লাল-পাতা, বশল্যকরনী<br>Pillow-weed	*Verbenaceae*	Herb
**129.***Litsea glutinosa* (Lour.) C. B. Rob. SKM722	বাঘলাল, পোঁজো<br>Bolly beech	*Lauraceae*	Tree
**130.***Ludwigia adscendens* (L.) H.Hara SKM834	কেঁচুনে, মুড়িগাছ<br>Water Primrose	*Onagraceae*	Herb
**131.***Luffa acutangula* (L.) Roxb.	ঝঙে<br>Ridged Gourd	*Cucurbitaceae*	Climber
**132.***Luffa aegyptiaca* Mill. SKM161	ধুঁদুল<br>Sponge gourd	*Cucurbitaceae*	Climber
**133.***Lycoperdon perlatum* Pers. SKM91	কুরকু<br>Puffball	*Agaricaceae*	Fruit body
**134.***Lygodium flexuosum* (L.) Sw. SKM180	ভুতরাজ<br>Maidenhair creeper	*Schizaeaceae*	Climber
**135.***Lysimachia arvensis* (L.) U.Manns & Anderb.<br>SKM721<br>Syn.- *Anagallis arvensis* L.	নীল<br>Scarlet pimpernel	*Primulaceae*	Herb
**136.***Madhuca longifolia* (J.Koenig ex L.) J.F.Macbr.<br>SKM187	মহুয়া<br>Mahua tree	*Sapotaceae*	Tree
**137.***Manilkara hexandra* (Roxb.) Dubard SKM341	ক্ষীরকুল<br>Ceylon wood	*Sapotaceae*	Tree
**138.***Martynia annua* L. SKM441	বাঘনখ<br>Tiger's claw	*Martyniaceae*	Shrub
**139.***Mesosphaerum suaveolens* (L.) Kuntze<br>Syn.- *Hyptis suaveolens* (L.) Poit. SKM128	বন তুলসী<br>Mint Bush	*Lamiaceae*	Herb
**140.***Mimosa pudica* L. SKM641	লজ্জাবতী<br>Touch-me-not	*Fabaceae*	Herb
**141.***Mitragyna parvifolia* (Roxb.) Korth. SKM761	গুলি কদম<br>Kaim	*Rubiaceae*	Tree
**142.***Moringa oleifera* Lam. SKM846	সজনে<br>Drumstick tree	*Moringaceae*	Tree
**143.***Murraya koenigii* (L.) Spreng. SKM985	কারিপাতা, বারাসাঙ্গা<br>Curry leaf tree	*Rutaceae*	Shrub

**144.***Musa paradisiaca* L. SKM31	কলা<br>Banana	*Musaceae*	Tree
**145.***Myristica fragrans* Houtt.	জাইফল<br>Nutmeg	*Myristicaceae*	Tree
**146.***Nelumbo nucifera* Gaertn. SKM152	পদ্ম<br>Indian lotus	*Nelumbonaceae*	Herb
**147.***Neolamarckia cadamba* (Roxb.) Bosser SKM24<br>Syn.- *Anthocephalus cadamba* (Roxb.) Miq.	কদম<br>Burflower-tree	*Rubiaceae*	Tree
**148.***Neptunia prostrata* (Lam.) Baill.<br>Syn.- *Neptunia oleracea* Lour. SKM551	জলের লজ্জাবতী<br>Water Mimosa	*Fabaceae*	Herb
**149.***Nerium oleander* L. SKM67<br>Syn.- *Nerium indicum* Mill.	করবী, রাজফুল<br>Oleander rose-bay	*Apocynaceae*	Shrub
**150.***Nicotiana rustica* L.	মতিহার তামাক<br>Aztec tobacco	*Solanaceae*	Herb
**151.***Nigella sativa* L.	কালো জিরে<br>Black cumin	*Ranunculaceae*	Herb
**152.***Nymphaea nouchali* Burm.f. SKM752	লাল শালুক<br>Red water lily	*Nymphaeaceae*	Herb
**153.***Ochna obtusata* DC. SKM816	চম্পা-বাহা<br>Ramdhan	*Ochnaceae*	Shrub
**154.***Oroxylum indicum* (L.) Kurz SKM519	বানাহাটা<br>Indian trumpet tree	*Bignoniaceae*	Tree
**155.***Ouret lanata* (L.) Kuntze<br>*Syn.- Aerva lanata* (L.) Juss. ex Schult. SKM123	লুপানি-আরা<br>Aerva	*Amaranthaceae*	Herb
**156.***Papaver somniferum* L.	পোস্তো<br>Opium poppy	*Papaveraceae*	Herb
**157.***Phoenix acaulis* Roxb. SKM205	বীর খেজুড়<br>Dwarf date palm	*Arecaceae*	Herb
**158.***Phyla nodiflora* (L.) Greene SKM362	ভুঁই-ওকরা<br>Frog fruit	*Verbenaceae*	Herb
**159.***Phyllodium pulchellum* (L.) Desv. SKM460<br>Syn.- *Desmodium pulchellum* (L.) Benth.	লোদাম<br>Showy Desmodium	*Fabaceae*	Shrub
**160.***Piper cubeba* L.f.	কাবাব চিনি<br>Java pepper	*Piperaceae*	Climber
**161.***Piper longum* L.	পিপুল, রাল্লী<br>Long pepper	*Piperaceae*	Climber
**162.***Piper nigrum* L.	গোলমরিচ<br>Black pepper	*Piperaceae*	Climber
**163.***Plantago ovata* Forssk.	ইসবগুল<br>Psyllium	*Plantaginaceae*	Herb
**164.***Pleurolobus gangeticus* (L.) J.St.-Hil. ex H.Ohashi &<br>K.Ohashi SKM171	শালপানী<br>Sal Leaved<br>Desmodium	*Fabaceae*	Herb

Syn.- *Desmodium gangeticum* (L.) DC.			
**165.***Plumbago zeylanica* L. SKM671	শ্বেত-চিতা<br>Ceylon leadwort	*Plumbaginaceae*	Shrub
**166.***Polygala arvensis* Willd. SKM108	গাইঘুরো<br>Field Milkwort	*Polygalaceae*	Herb
**167.***Polygala crotalarioides* Buch.-Ham. ex DC. SKM227	নীল-কাঠী<br>Indian Milkwort	*Polygalaceae*	Herb
**168.***Portulaca oleracea* L. SKM720	নুনি-আরা<br>Common purslane	*Portulacaceae*	Herb
**169.***Premna herbacea* Roxb. SKM261	ভুঁই-কম্বল<br>Stemless premna	*Lamiaceae*	Herb
**170.***Psidium guajava* L. SKM370	পিয়ারা<br>Guava	*Myrtaceae*	Tree
**171.***Pueraria tuberosa* (Roxb. ex Willd.) DC. SKM188	ভুঁই-কুমড়ো<br>Indian kudzu	*Fabaceae*	Climber
**172.***Rauvolfia serpentina* (L.) Benth. ex Kurz SKM617	সর্পগন্ধা<br>Indian snakeroot	*Apocynaceae*	Herb
**173.***Rivea hypocrateriformis* Choisy SKM77	বন পুঁই<br>Common Night Glory	*Convolvulaceae*	Climber
**174.***Rotheca serrata* (L.) Steane & Mabb. SKM315<br>Syn.- *Clerodendrum serratum* (L.) Moon	বারাঙ্গী, সরম-লিতুর<br>Blue fountain bush	*Lamiaceae*	Shrub
**175.***Ruellia prostrata* Poir. SKM417	চটপটী<br>Bell Weed	*Acanthaceae*	Herb
**176.***Ruellia tuberosa* L. SKM316	রানু-রান্ড<br>Meadow Weed	*Acanthaceae*	Herb
**177.***Santalum album* L. SKM311	শ্বেত চন্দন<br>Sandalwood	*Santalaceae*	Tree
**178.***Schoenoplectiella articulata* (L.) Lye SKM313<br>Syn.- *Scirpus articulatus* L.	পাতি-পটপটি<br>Jointed Sedge	*Cyperaceae*	Herb
**179.***Scoparia dulcis* L. SKM10	মিদরি<br>Sweet-broom	*Plantaginaceae*	Herb
**180.***Semecarpus anacardium* L.f. SKM513	ভেলা<br>Marking nut	*Anacardiaceae*	Tree
**181.***Senegalia catechu* (L.f.) P.J.H.Hurter & Mabb. SKM89<br>*Syn.- Acacia catechu* (L.f.) Willd.	খয়ের<br>Black Catechu	*Fabaceae*	Tree
**182.***Senna occidentalis* (L.) Link SKM105<br>Syn.-*Cassia occidentalis* L.	সোনা পাতা, ভেড়া-দিড়িঙ<br>Ant bush	*Fabaceae*	Shrub
**183.***Sesamum indicum* L.	সাদা তিল<br>Sesame	*Pedaliaceae*	Herb
**184.***Seseli diffusum* (Roxb. ex Sm.) Santapau &Wagh SKM788	বন জোয়ান<br>Indian celery	*Apiaceae*	Herb

**185.***Shorea robusta* Gaertn. SKM81	শাল<br>Sal tree	*Dipterocarpaceae*	Tree
**186.***Sida cordifolia* L. SKM190	বেড়েলা<br>Heart-Leaf Sida	*Malvaceae*	Herb
**187.***Sida rhombifolia* L. SKM294	শ্বেত-বেড়েলা<br>Arrow-leaf Sida	*Malvaceae*	Herb
**188.***Smilax ovalifolia* Roxb. ex D.Don SKM201	রাম-পান, রাজ-পান<br>Kumarika	*Smilacaceae*	Climber
**189.***Solanum glaucophyllum* Desf. SKM193<br>Syn.- *Solanum glaucum* Dunal	বিশল্যকরবী<br>Waxy leaf<br>Nightshade	*Solanaceae*	Shrub
**190.***Solanum sisymbriifolium* Lam. SKM29	শ্বেত কন্টিকারী<br>Sticky nightshade	*Solanaceae*	Shrub
**191.***Solanum torvum* Sw. SKM601	গোঠবেগুন<br>Turkey Berry	*Solanaceae*	Shrub
**192.***Solanum violaceum* Ortega<br>*Syn.- Solanum indicum* L. SKM119	বৃহতি<br>Indian Nightshade	*Solanaceae*	Shrub
**193.***Solanum virginianum* L.<br>Syn.- *Solanum surattense* Burm. f. SKM115	কন্টিকারী<br>Surattense<br>Nightshade	*Solanaceae*	Herb
**194.***Soymida febrifuga* (Roxb.) A. Juss. SKM99	রহিন, রক্ত রহিম<br>Indian Redwood	*Meliaceae*	Tree
**195.***Sphaeranthus indicus* L. SKM901	ভুঁইকদম<br>East Indian Globe<br>Thistle	*Asteraceae*	Herb
**196.***Spondias pinnata* (L. f.) Kurz SKM307	আমড়া<br>Hog plum	*Anacardiaceae*	Tree
**197.***Streblus asper* Lour. SKM203	শ্যাওরা<br>Siamese rough bush	*Moraceae*	Tree
**198.***Strobilanthes hirta* (Vahl) Blume SKM791<br>Syn.- *Hemigraphis hirta* (Vahl) T.Anderson	বান-টুরটুর-আরা, মুশাকানি<br>Red Ivy	*Acanthaceae*	Herb
**199.***Strychnos nux-vomica* L. SKM402	কুচিলা<br>Nux vomica	*Loganiaceae*	Tree
**200.***Swertia chirayita* (Roxb.) H.Karst.	চিরতা<br>Chirayata	*Gentianaceae*	Herb
**201.***Swietenia macrophylla* King. SKM624	মেহোগনী<br>Broad-leafed<br>Mahogany	*Meliaceae*	Tree
**202.***Syzygium aromaticum* (L.) Merr. & L.M.Perry	লবঙ্গ<br>Clove	*Myrtaceae*	Tree
**203.***Tacca leontopetaloides* (L.) Kuntze SKM503	ধণ্ড<br>Indian Arrow Root	*Dioscoreaceae*	Herb

**204.** *Tamarindus indica* L. SKM211	তেঁতুল, জোজো<br>Tamarind	*Fabaceae*	Tree
**205.** *Tamilnadia uliginosa* (Retz.) Tirveng. & Sastre SKM1138<br>Syn.- *Catunaregam uliginosa* (Retz.) Sivar.	পিরার-দারি<br>Divine Jasmine	*Rubiaceae*	Tree
**206.** *Terminalia arjuna* (Roxb. ex DC.) Wight & Arn. SKM129	অর্জুন<br>Arjun tree	*Combretaceae*	Tree
**207.** *Terminalia chebula* Retz.	হরিতকী<br>Myrobalan	*Combretaceae*	Tree
**208.** *Termitomyces heimii* Natarajan SKM124	শিব ছাতু<br>Termite mound mushroom	*Lyophyllaceae*	Fruit body
**209.** *Tinospora sinensis* (Lour.) Merr. SKM251<br>Syn.- *Tinospora cordifolia* (Willd.) Miers	পদ্ম গুলঞ্চ<br>Heart-leaved moonseed	*Menispermaceae*	Climber
**210.** *Trachyspermum ammi* (L.) Sprague	জোয়ান<br>Caraway	*Apiaceae*	Herb
**211.** *Tragia involucrata* L. SKM75	সেঙ্গেল-সেঙ, বিচুতি<br>Indian stinging nettle	*Euphorbiaceae*	Climber
**212.** *Trapa natans* L. SKM306	দেশী পানিফল<br>Water nut	*Lythraceae*	Herb
**213.** *Trianthema portulacastrum* L. SKM909	কুল্পী আরা<br>Desert horse purslane	*Aizoaceae*	Herb
**214.** *Tribulus terrestris* L. SKM710	গোখুর<br>Puncture Vine	*Zygophyllaceae*	Herb
**215.** *Tripidium bengalense* (Retz.) H.Scholz<br>Syn.- *Saccharum bengalense* Retz. SKM209	সর-ঘাস<br>Munj sweet cane	*Poaceae*	Shrub
**216.** *Typhonium trilobatum* (L.) Schott SKM811	খারকন<br>Bengal Arum	*Araceae*	Herb
**217.** *Uraria lagopodiodes* (L.) DC. SKM612	চাকুলে<br>Hare Foot Uraria	*Fabaceae*	Herb
**218.** *Urena lobata* L. SKM304	লাঠা<br>Caesar weed	*Malvaceae*	Shrub
**219.** *Vachellia nilotica* subsp. *indica (*Benth.) Kyal. & Boatwr. SKM02<br>*Syn.- Acacia nilotica* subsp. *indica* (Benth.) Brenan	বাবলা<br>Prickly acacia	*Fabaceae*	Tree
**220.** *Vanda tessellata* (Roxb.) Hook. ex G.Don SKM451	বাঙ্কি<br>Grey orchid	*Orchidaceae*	Herb
**221.** *Ventilago denticulata* Willd. SKM106	বোঙা-সার-জোম<br>Red creeper	*Rhamnaceae*	Climber
**222.** *Vicia lens* (L.) Coss. & Germ.<br>Syn.- *Lens culinaris* Medik.	মশুর<br>Lentil	*Fabaceae*	Herb
**223.** *Vitex negundo* L. SKM208	নিসিন্দা, সিন্দুয়ারী	*Lamiaceae*	Shrub

	Chinese chaste tree		
**224.** *Volkameria inermis* L. SKM564<br>Syn.- *Clerodendrum inerme* (L.) Gaertn.	বন-জাই<br>The glory bower	*Lamiaceae*	Shrub
**225.** *Wattakaka volubilis* (L.f.) Stapf. SKM182<br>Syn.- *Dregea volubilis* (L.f.) Benth. ex Hook.f.	পানশুঁট, মারাঙকোঙ্গোট<br>Green Milkweed Climber	*Apocynaceae*	Climber
**226.** *Xanthium strumarium* L. SKM301	ওকরা<br>Rough cocklebur	*Asteraceae*	Shrub
**227.** *Xenostegia tridentata* (L.) D.F.Austin & Staples SKM14<br>Syn.- *Merremia tridentata* (L.) Hallier f.	জামজুরি-আরা<br>African morning vine	*Convolvulaceae*	Climber
**228.** *Zingiber officinale* Roscoe	আদা<br>Ginger	*Zingiberaceae*	Herb
**229.** *Zingiber zerumbet* (L.) Roscoe ex Sm.	মহাবীর বচ<br>Pinecone ginger	*Zingiberaceae*	Herb
**230.** *Ziziphus jujuba* Mill. SKM444	কুল, জেনুম-দারি<br>Chinese jujube	*Rhamnaceae*	Tree
**231.** *Ziziphus nummularia* (Burm.f.) Wight & Arn. SKM48	ভুবাদারি<br>Wild jujube	*Rhamnaceae*	Shrub
**232.** *Ziziphus oenoplia* (L.) Mill. SKM57	শিয়াকুল<br>Jackal jujube	*Rhamnaceae*	Shrub
