# Supplementary material for: Inventorization and Consensus Analysis of Ethnoveterinary Medicinal Knowledge Among the Local People in Eastern India: Perception, Cultural Significance, and Resilience
Source: Front Pharmacol. 2022 Apr 29;13:861577. doi: 10.3389/fphar.2022.861577 (PMC9099233; doi:10.3389/fphar.2022.861577)
Supplement: Supplementary file 4 [file Table3.pdf]

Supplementary Table S3. List of health conditions of the livestock recorded from the study area with their diagnostic symptoms

Major illness and their local names	Diagnostic symptoms
1. Arthritis/ rheumatism<br>*Shimola rog*	• Lameness with numbness to the affected joint can be observed<br>• Inflammation in the joints<br>• Restricted movement
2. Bloat<br>*Dhonrabai*	• Colic/ abdominal pain<br>• Animal try to kick the belly with its leg and stands with its back legs stretching wide apart<br>• Become aggressive
3. Bloody dysentery<br>*Rakta amashay*	• Watery faeces appear reddish black in colour due to the presence of blood in it.
4. Body ache (muscle pain)<br>*Gaa-vara*	• Unable to move and rise<br>• Sometimes stiffness of muscle can be sensed upon touching<br>• Unusual vocalizations
5. Body swelling due to cold<br>*Jol-sannipat*	• Fever (102-104°F)<br>• Nasal discharge<br>• Swelling throughout the body<br>• Loss of appetite
6. Bone fracture (or dislocation of joints)<br>*Har-vanga*	• Injured animals don't move<br>• Sometimes swelling appears in the affected part<br>• Affected leg keeps pulled up during movement
7. Broken horn<br>*Sing-vanga*	• Crack is observed at the base of the horn or sometimes hard exoskeletal covering is totally exfoliated
8. Diarrhoea	• Animals pass watery faeces many times a day<br>• The droppings are loose, runny with bad smell and dark green or dark brown colour<br>• Loss of appetite
9. Cold, cough and runny nose<br>*Sannipat*	• Fever (102-104°F)<br>• Nasal discharge<br>• Coughing and sneezing<br>• Drooping down of head
10. Constipation<br>*Kostho/ Kater-ech*	• Continuous shaking of the tail<br>• Very often animal try to hold the tail in upraised posture<br>• Uneasiness<br>• Painful and stiff defecation
11. Poor lactation<br>*Dudh-namla*	• Sudden and abnormal decrease in production of milk
12. Delay in parturition	• Exceed of the normal gestation period of domesticated cow (average 280 days) by 20-30 days<br>• Continuous whitish sticky discharge from the vagina of a gravid cow
13. Delay in onset of estrus cycle	• Mature heifer becomes restless and aggressive
14. Drowsiness<br>*Jhimuni*	• Lameness<br>• Shivering and rapid breathing<br>• Loss of appetite
15. Dysentery<br>*Amashay*	• Frequent release of watery and /or slimy fecal matter<br>• Animal looks very weak with sunken eyes
16. Dyspepsia<br>*Daka-bang-renget*	• Animal remain separate from the grazing herd<br>• Irregular rumination<br>• Stiff defecation<br>• Reluctant to eat
17. Ectoparasite (lice and tick)	• Animals lick or bite their body very aggressively<br>• Frequently rub their body against wall or tree trunk<br>• Sometimes large ticks are witnessed throughout the body surface

	• Skin may become rough<br>• Hair loss<br>• Wounds and bruises found in severe cases
18. Fever (fever with stomach problems and pulmonary congestion, high fever) *Jwar/ Rhuo*	• Short term raise of body temperature<br>• Shivering<br>• Lameness and muscular stiffness<br>• Loss of appetite
19. Foot and mouth disease (FM); *Pankui*	• Fever with 104-105°F of body temperature<br>• Profuse salivation<br>• Vesicles appear in mouth and between the hooves<br>• Lameness observed
20. Foot rot *Pankui*	• Necrotic wounds between the hooves or around the top of the hoof<br>• Severe lameness; restricted movement
21. Gastro-intestinal discomfort	• Rapid and uneasy movement of leg<br>• Slight rise of body temperature<br>• Reluctant to eat
22. Haematuria *Rakta-prasrab*	• Blood in urine<br>• Drowsiness<br>• Loss of appetite<br>• Rise of body temperature
23. Headache *Matha batha*	• Aggressiveness<br>• Frequent shaking of head and sometimes rubbing of it against a wall<br>• Restless condition
24. General weakness *Hormo-langa*	• Decreased milk secretion<br>• Dullness and freezing of leg<br>• Dyspepsia<br>• Aggressiveness towards new born
25. Helminthiasis *Krimi rog*	• Anorexia<br>• Pot-belly appearance of the abdomen<br>• Constipation<br>• Rough coat on the skin
26. Hemorrhagic septicemia (HS)	• High fever (104°–106°F) lasts up to 3 -5 days<br>• Restlessness and reluctance to move<br>• Excessive salivation<br>• Lacrimation of eyes and nasal discharge<br>• Subcutaneous swelling appears in the pharyngeal region
27. Indigestion	• The abdomen became large on the one side.<br>• Loss of appetite<br>• Protrusion of the tongue and<br>• Animal keep the shoulder stretched throughout the day.
28. Infectious disease *Dushit-rog*	• Rise in body temperature<br>• Increase in pulse rate and respiration<br>• At a time many animals will show same symptom
29. Infertility	• Repeated failure in conceiving after successful mating with a potent bull or artificial insemination (AI)
30. Listeriosis *Gai-ghuro*	• Stiffness of neck and jaw<br>• Unusual movement of limbs and tendency to move in circles
31. Liver trouble *Dangra-piley*	• Loss of appetite<br>• Slight rise of the body temperature<br>• Sometimes fluid develop under the skin and itching occurs
32. Loose motion *Chherani/ Leher-ech*	• Unusual growling or rumbling of stomach<br>• Watery stool<br>• Slow breathing<br>• Loss of appetite
33. Loosened teeth *Kalasashru*	• Loosening of permanent teeth
34. Mastitis	• Inflammation of the mammary gland and painful tits

Thunko/ Dudh-thunko	• Cracked nipples<br>• Sometimes blood and puss come out with milk<br>• High body temperature
35. Miscarriage	• Loss of the fetus between the age of 42 - 260 days followed by high fever<br>• Restlessness
36. Mouth sore<br><br>*Mukhe gha*	• Blisters appears in and outside of the mouth<br>• Weight loss<br>• Unable to eat<br>• Lameness<br>• Trembling lips and foaming of mouth
37. Opacity of cornea (Kerato-conjunctivitis)<br>*Go-Chhani*	• Whitening of eye ball<br>• Watering of eyes<br>• Blurred vision<br>• Animal avoid direct sun light
38. Poisonous bite (bite of snake, dog, insect)<br>*Sapure*	• Localized swelling and irritation, sometimes bleeding from bite wounds<br>• Vomiting<br>• Foaming mouth with trembling lips<br>• Dilated pupils<br>• Rapid breathing and/or panting<br>• Freezing legs (initiated in the hind legs and succeeding towards the head)
39. Post-partum bleeding	• Drop wise sticky bloody discharge occurs through the vagina up to 5-7 days after calving
40. Prolapsed uterus<br>*Bhanral nama*	• Within 24 hrs of calving, uterus is pushed out of the animal's vulva like a bladder and hangs down
41. Pulmonary congestion (Tonsillitis, throat sore, swelling of throat, dewlap)	• Animal is dull and off feed<br>• Coughing, sneezing, redness of eye and nasal discharges<br>• Body temperature may rise<br>• Difficulty in breathing
42. Retention of milk<br>*Dudh-thunko*	• Very low milk production after parturition up to 10-15 days<br>• Loss of appetite
43. Retention of placenta<br>*Ful na pora*	• Delay in expulsion of placenta after parturition up to 72 hours<br>• Bloody discharges through vagina<br>• Uneasiness
44. Unusual urination	• Discontinuous urination with prolonged interval<br>• Sometimes drop wise urination occurs throughout the day
45. Rhinorrhoea<br>*Sonra rog*	• Continuous discharge of slimy matter through nasal openings<br>• Trouble in breathing
46. Shoulder inflammation/ Sore on the shoulder	• Hard and stiff subcutaneous areas on the shoulder<br>• Inflammation of the shoulder<br>• Crack on the skin surface
47. Stomach pain<br>*Pet-mochor/ Lai-haso*	• Restlessness<br>• Reluctant to eat<br>• Unwilling to rise up
48. Stop mastication<br>*Jabar na kata*	• Irregular rumen movement<br>• Stop eating
49. Swelling of body due to food poisoning<br>*Aasor*	During grazing time, intake of insecticide or some other poisonous substances like spider web, exoskeleton of snake, etc. by the animal creates the following symptoms -<br><br>• Excessive salivation<br>• Open mouth and the tongue hangs out<br>• Diarrhoea and vomiting<br>• Slow breathing

	• Irregular swellings throughout the body
50. Wart / Tumor<br>*Aab*	• Hard lump of tissue
51. Wounds (fresh cuts, ulcerated wound, septic wound, maggot infested wound, suppurating wound)<br>*Kata Gha / Khato*	• Injury can be observed mainly around the ear, sternum and fore and hind legs.<br>• Open wound become septic and suppurating due to infection.<br>• Flies make the wound infected and maggoty
